# Structure of the antenna complex expressed during far-red light photoacclimation in *Synechococcus* sp. PCC 7335

**DOI:** 10.1016/j.jbc.2023.105590

**Published:** 2023-12-22

**Authors:** Christopher J. Gisriel, Gaozhong Shen, Gary W. Brudvig, Donald A. Bryant

**Affiliations:** 1 Department of Chemistry, Yale University, New Haven, Connecticut, USA; 2 Department of Biochemistry and Molecular Biology, The Pennsylvania State University, University Park, Pennsylvania, USA; 3 Department of Molecular Biophysics and Biochemistry, Yale University, New Haven, Connecticut, USA

**Keywords:** photosynthesis, cyanobacteria, allophycocyanin, phycocyanobilin, far-red light photoacclimation, FaRLiP, energy transfer, light harvesting, cryo-EM, phycobilisome

## Abstract

Far-red light photoacclimation, or FaRLiP, is a facultative response exhibited by some cyanobacteria that allows them to absorb and utilize lower energy light (700–800 nm) than the wavelengths typically used for oxygenic photosynthesis (400–700 nm). During this process, three essential components of the photosynthetic apparatus are altered: photosystem I, photosystem II, and the phycobilisome. In all three cases, at least some of the chromophores found in these pigment–protein complexes are replaced by chromophores that have red-shifted absorbance relative to the analogous complexes produced in visible light. Recent structural and spectroscopic studies have elucidated important features of the two photosystems when altered to absorb and utilize far-red light, but much less is understood about the modified phycobiliproteins made during FaRLiP. We used single-particle, cryo-EM to determine the molecular structure of a phycobiliprotein core complex comprising allophycocyanin variants that absorb far-red light during FaRLiP in the marine cyanobacterium *Synechococcus* sp. PCC 7335. The structure reveals the arrangement of the numerous red-shifted allophycocyanin variants and the probable locations of the chromophores that serve as the terminal emitters in this complex. It also suggests how energy is transferred to the photosystem II complexes produced during FaRLiP. The structure additionally allows comparisons with other previously studied allophycocyanins to gain insights into how phycocyanobilin chromophores can be tuned to absorb far-red light. These studies provide new insights into how far-red light is harvested and utilized during FaRLiP, a widespread cyanobacterial photoacclimation mechanism.

Cyanobacteria are photosynthetic microorganisms that exhibit profound diversity. They are found in almost every imaginable habitat and thus account for a large fraction of global oxygenic photosynthesis (1, 2). Phycobiliproteins (PBPs), which are vibrantly colored, water-soluble proteins that bind linear tetrapyrrole chromophores called bilins, are the major light-harvesting proteins in most cyanobacteria (3, 4, 5, 6). Light energy absorbed by PBPs is principally transferred to photosystem II (PSII), which contains relatively fewer chlorophyll (Chl) chromophores than photosystem I (PSI) (7, 8). Energy transfer from PBPs to PSI also occurs and can be important under some growth conditions (9, 10).

The PBP superfamily descends from a single ancestral protein (3, 11). This ancestral antenna protein evolved into the current, highly diversified superfamily through many rounds of gene duplication and divergence. Major PBP subfamilies include phycoerythrins (λ_max_ = 560 nm), phycoerythrocyanins (λ_max_ = 590 nm), phycocyanins (PC; λ_max_ = 620 nm), and allophycocyanins (AP; λ_max_ = 650–710 nm) (3, 5, 11). Some subfamilies, especially the AP family, include numerous variants that have specialized functions for the assembly, light-harvesting, and/or energy transfer functions of these proteins (5, 11, 12). The fundamental structural unit of all PBPs is an (αβ) heterodimer, which is typically referred to as a “monomer” but is more correctly denoted a protomer. Each subunit carries at least one and up to three bilins, which are usually but not always covalently bound to the protein through thioether linkages to Cys sidechains (3, 5, 13). Protomers oligomerize to form toroid-shaped trimers, (αβ)_3_, or hexamers, (αβ)_6_ (5), although an AP variant was recently described that forms helical nanotubes (14). Linker proteins containing so-called REP domains (REPeat domains, pfam00427) thread through the center of these toroids and stabilize the binding of two trimers to form a hexamer (3, 5, 15). These interactions further allow PBPs to form cylindrical stacks that can then assemble into diverse supramolecular structures known collectively as phycobilisomes (PBS) (16, 17).

Several structural classes of PBS occur, including those that are described as bundle-shaped, paddle-shaped, hemidiscoidal, hemiellipsoidal, block-shaped, and rod-shaped (5, 17, 18, 19, 20, 21, 22, 23, 24, 25, 26, 27). Hemidiscoidal PBS are most common in cyanobacteria; they exhibit a core substructure comprising two, three, or five cylinders of AP and some minor AP variants (5). Six to eight peripheral rods, which are stacks of hexameric phycocyanin and phycoerythrin or phycoerythrocyanin, if present, radiate from two sides of the core (5, 17). Rod-shaped PBS, which are about 750 kDa with ∼54 bilin chromophores (26), are much smaller than average hemidiscoidal PBS, which range in size from about 4.5 MDa to 7 MDa with 300 to 750 bilin chromophores (22, 23, 24). The block-shaped and hemiellipsoidal PBS of red algae can reach sizes up to 17 MDa and carry 1600 to 2000 bilin chromophores (20, 21). The diversity of PBPs and the PBS classes observed in nature reflect the evolution of organisms driven by differences in their light niches.

Most cyanobacteria are obligate phototrophs and are therefore highly dependent on the light energy they harvest for photosynthesis. Consequently, cyanobacteria have evolved many mechanisms to maximize their photosynthetic performance under diverse light intensities and/or wavelength conditions (28, 29). A prime example of this is called far-red light (FRL) photoacclimation or FaRLiP (11, 30, 31, 32). During FaRLiP, three key components of the photosynthetic apparatus are altered: PSI, PSII, and the PBS. In the photosystems, many subunits expressed in visible light (VL) are replaced with FRL-specific isoforms. In these FRL-specific subunits, some Chl-binding sites that typically bind Chl *a* in the VL-homologs are modified to instead bind FRL-absorbing Chls *d* or *f* molecules. Structural investigations have revealed the differences between the photosystem subunits expressed during growth in VL and FRL and have identified the specific binding locations of the Chls *d* and *f* molecules present in the latter (33, 34, 35, 36, 37, 38). Less is understood about how PBPs are altered during FaRLiP. When cyanobacteria are grown in VL, most produce hemidiscoidal PBS as noted above (5). However, smaller core substructures are produced during FaRLiP that contain AP subfamily members that absorb FRL (39, 40). When cells are grown in FRL, the FaRLiP-capable *Synechococcus* sp. PCC 7335 (hereafter *Synechococcus* 7335) produces bicylindrical cores that absorb maximally at ∼710 nm and specifically comprise seven subunits ApcB2, ApcD2, ApcD3, ApcD5, ApcE2, ApcF, and ApcC (39, 40, 41). Although ApcF and ApcC are also found in the hemidiscoidal PBS present during growth in VL, the other five proteins are only produced during FaRLiP (30, 39, 40). Mutants lacking any one of these five FRL-specific subunits lack the entire bicylindrical core complex and do not accumulate any other FRL-absorbing AP-type subunits. Furthermore, such mutants do not accumulate WT levels of Chl *d* and are unable to grow in FRL (41). Determining the subunit composition, arrangement, and chromophore properties of the PBPs expressed during FaRLiP is vital for completing a molecular understanding of this fascinating and widespread cyanobacterial acclimation mechanism.

To address these issues, we have isolated a FRL-absorbing PBP complex from cells of the marine cyanobacterium *Synechococcus* 7335 grown in FRL and solved its structure to a global resolution of 2.78 Å by single-particle, cryo-EM. The structure reveals the locations of all variant, FRL-absorbing, AP subunits, and thus the structure allows a comprehensive comparison of chromophore structures and protein environments compared to other AP variants. This structure also complements the molecular structures of the FRL-absorbing PSI and PSII (FRL-PSII) complexes and provides a nearly complete picture of how the photosynthetic apparatus is altered during FaRLiP. The fraction containing the FaRLiP-AP core complexes also contained ribulose 1,5-bisphosphate carboxylase oxygenase (Rubisco), and the structure of this enzyme was solved to a global resolution of 2.35 Å.

## Results

### Characterization and imaging

FaRLiP-AP core complexes were isolated by using two rounds of centrifugation on linear sucrose gradients as described in the Experimental procedures. After the first sucrose gradient step, the isolated complexes had absorbance maxima at 648 and 710 nm with a single fluorescence emission maximum of 730 nm at 77 K (Fig. S1), values which are similar to previous results (39, 40), but this sample was heavily contaminated with other proteins as judged by SDS-PAGE and proteomic analysis by mass spectroscopy, making it unsuitable for cryo-EM. After a second round of centrifugation on sucrose gradients, the band appeared better defined, but the absorbance spectrum of the complex had changed subtly, with maxima at 650 and 710 nm (Fig. S1). However, the fluorescence emission spectrum at 77 K had changed dramatically. The emission spectrum had maxima at 640 nm and 716 nm and a shoulder at 730 nm (Fig. S1). This result strongly suggested that although contaminating proteins were removed by the second sucrose gradient centrifugation step, some dissociation of the FaRLiP-AP core complexes had occurred. Interestingly, compared to the helical FRL-AP produced by *Thermostichus* sp. (14), the complexes isolated from *Synechococcus* 7335 have significantly broader and enhanced absorbance around 650 nm and notably increased absorbance from 710 to 725 nm (Fig. S2). SDS-PAGE analysis and chymotryptic peptide fingerprinting by MS/MS spectrometry showed that the complexes isolated from *Synechococcus* 7335 contained the same seven protein subunits as previously described (39) (Fig. S3 and Table S1). These analyses also still indicated the presence of several contaminating proteins (Fig. S3). To determine whether the protein sample was sufficiently pure for single-particle cryo-EM, the complexes were negatively stained and subjected to transmission electron microscopy (Fig. S4). Micrographs revealed a heterogeneous particle distribution; therefore, the FaRLiP-AP core complexes were further purified by size-exclusion chromatography (Fig. S4). Following this chromatography, a negatively stained sample showed individual cores rather than bicylindrical cores, but these appeared to be much more monodisperse. Thus, the sample was deemed suitable for structure determination by single-particle cryo-EM.

### Cryo-EM and subunit arrangement

The protein sample was plunge frozen for cryo-EM as described in the Experimental procedures. The sample was initially imaged and screened using a 200 kV Glacios cryo-transmission electron microscope (Fig. S5) and subsequently was used for high-resolution data collection on a 300 kV Titan Krios transmission electron microscope. Processing of the latter dataset revealed two types of particles: asymmetric classes that derived from the cylindrical FaRLiP-AP core complex and classes with strong bilateral symmetry, which derived from form I Rubisco. The latter contained eight large RbcL and eight small RbcS subunits, and its structure was determined to a global resolution of 2.35 Å using D4 symmetry (Figs. S6, S7, and Table S2). The derived structure is very similar to numerous other cyanobacterial Rubisco structures. A brief discussion and comparison to the Rubisco structure determined previously from *Synechococcus* sp. PCC 6301 (PDB 1RBL) (42) is provided in Text S1, Figs. S8, and S9.

**Figure 1:**
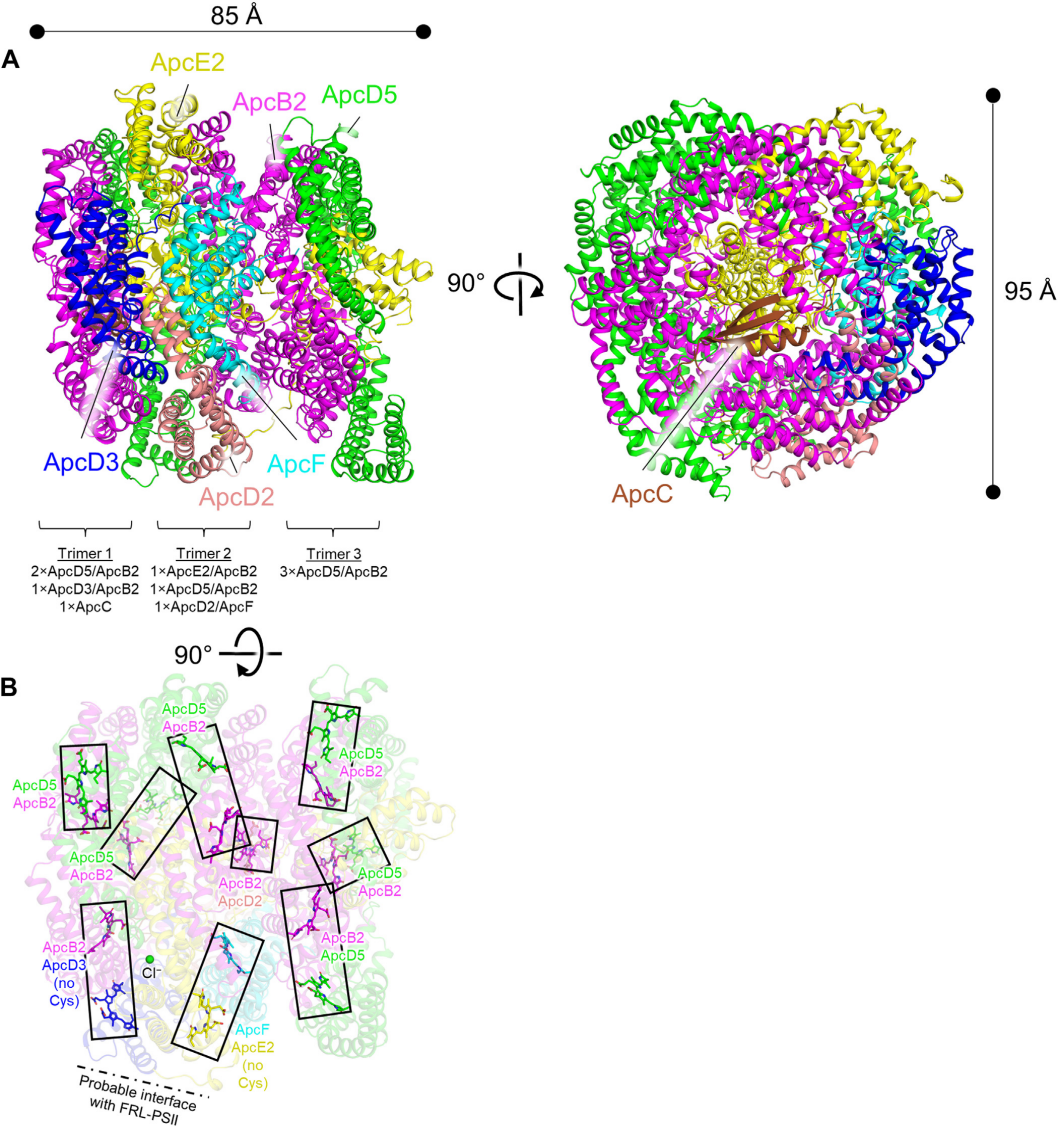
**Subunit and chromophore organization of the FaRLiP-AP core structure.***A*, dimensions of the FaRLiP-AP core structure are shown and subunits are colored and labeled individually. Only one ApcB2 and one ApcD5 subunit are labeled, although there are eight and five of these subunits, respectively. The composition of the protomers that make up each trimer are shown below the view on the *left*. *B*, chromophore composition of the FaRLiP-AP core. Each pair of chromophores is boxed. Note that the chromophores of ApcD3 and ApcE2 are not covalently linked and should therefore exhibit the most *red*-shifted absorbance. FaRLiP, far-red light photoacclimation; PBS, phycobilisome.

**Figure 2:**
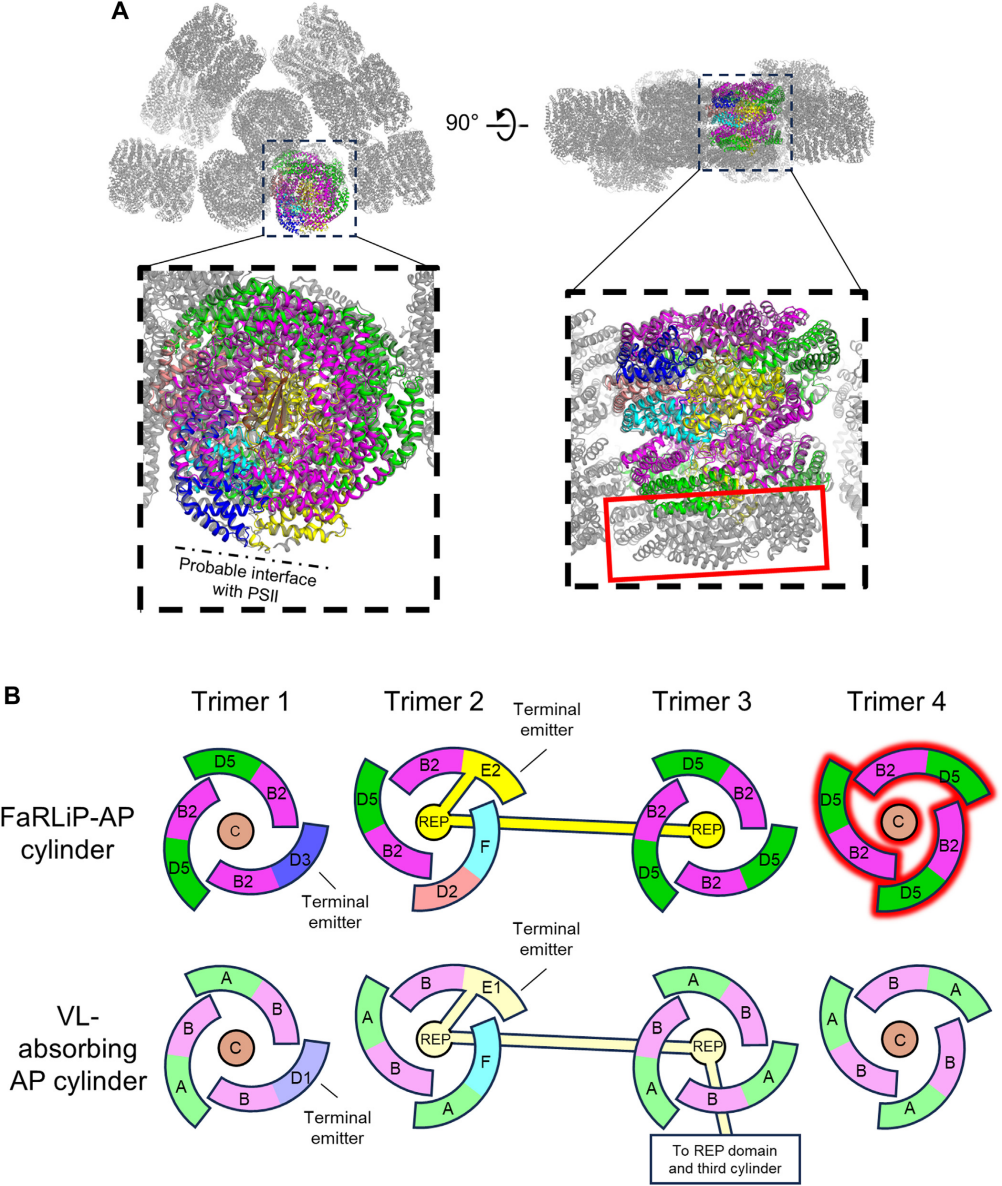
**Comparison of a FaRLiP-AP core to a standard AP core from a hemidiscoidal phycobilisome.***A*, superposition of the FaRLiP-AP core with an AP core cylinder from a hemidiscoidal PBS. The slightly transparent *gray* structure is the hemidiscoidal PBS from *Synechococcus* 7002 (PDB 7EXT). Relative to the bottom cylinder of a typical AP core substructure, the FaRLiP-AP core cylinder is missing one trimeric toroid (*red box*). *B*, cartoon diagram of trimer compositions in a core. The labels correspond to the AP type of each subunit, for example, ApcD5 = “D5” and ApcA = “A”. ApcC (labeled “C”) and the REP domains of ApcE2 and ApcE1 are linkers. Subunits with a *red* glow are those presumed to be missing in the FaRLiP-AP core structure. ApcF and ApcC are shared between the FRL and VL structures and are thus shown in *paler colors*. FaRLiP, far-red light photoacclimation; FRL, far-red light; PBS, phycobilisome.

The FaRLiP-AP core structure was solved to a global resolution of 2.78 Å based on the map-to-map Fourier Shell Correlation (Figs. S10, S11, and Table S2), although some map regions were challenging to model due to preferential orientation (see Experimental procedures). It is generally shaped like a cylinder, ∼85 Å in length and ∼95 Å in diameter (Fig. 1). Nineteen subunits were identified: eight ApcB2 (β-type subunit), six ApcD5 (α-type subunit), one ApcE2 (α-type subunit with two REP domains (Pfam00427)), one ApcD3 (α-type subunit), one ApcD2 (α-type subunit), one ApcF (β-type subunit), and one ApcC (core linker) (Figs. 1 and 2). Other than the ApcC subunit, which is a core linker protein that assembles into the opening of a trimeric AP toroid, the subunits are arranged as (αβ) heterodimers that further assemble into three (αβ)_3_ toroids (Figs. 1 and 2). Trimer 1 and trimer 2 are closely associated, forming a face-to-face hexamer, and trimer 3 is more separated in a tail-to-tail assembly with the trimer 1-trimer 2 toroid (Fig. 1*A*). Two of the three protomers in trimer 1 are ApcD5/ApcB2, and one is ApcD3/ApcB2. Trimer 1 also includes the single copy of ApcC in the structure. Each protomer in trimer 2 is unique, including one ApcE2/ApcB2 protomer, one ApcD5/ApcB2 protomer, and one ApcD2/ApcF protomer. All three protomers in trimer 3 are identical, ApcD5/ApcB2.

One anion was identified and tentatively modeled as chloride (Figs. 1*B* and S12), and each subunit except ApcC binds a single phycocyanobilin chromophore. As previously observed in oligomeric structures of AP, AP-B, and helical FRL-AP (14, 43, 44), the phycocyanobilin chromophores form pairs (Fig. 1*B*), in which two bilins in a pair are ∼20 Å apart (center-to-center). All phycocyanobilin chromophores are covalently bound to their respective subunits except those bound to ApcD3 and ApcE2. As previously suggested (39, 45, 46), these chromophores should contribute the most red-shifted absorbance due to the absence of a thioether bond from a Cys sidechain to the vinylidene moiety on pyrrole ring A, which therefore extends the conjugation system in the chromophore. The chromophores of ApcD3 and ApcE2 therefore likely serve as the terminal emitters in the core complex, linking the other phycocyanobilins in the FaRLiP-AP core complex to the Chl molecules in FRL-PSII. This is consistent with their positions proximal to PSII when superimposed onto an AP core from a typical hemidiscoidal PBS (Fig. 2*A*).

### Expected subunit composition *in vivo*

FaRLiP-AP cores have been suggested to associate into a bicylindrical configuration (39, 40), which is supported by the presence of only two linking REP domains that should each form a scaffold for a single core cylinder. Additionally, the FaRLiP-AP core was expected to contain four (αβ)_3_ toroids and two ApcC subunits rather than three (αβ)_3_ toroids and one ApcC subunit. This can be visualized by superimposing the FaRLiP-AP core structure onto one of the bottom AP core cylinders from a typical hemidiscoidal PBS like that from *Synechococcus* sp. PCC 7002 (hereafter *Synechococcus* 7002) (22) (Fig. 2*A*). It is possible that the FaRLiP-AP core only contains three (αβ)_3_ toroids *in vivo*, which would be analogous to some AP core structures of red algal PBS (20, 21, 25), but four observations strongly support the hypothesis that an (αβ)_3_ toroid and its closely interacting ApcC subunit were lost from the FaRLiP-AP core during the extended purification used in this study. First, the fluorescence emission spectrum was altered following the second sucrose density gradient relative to the first, suggesting a disconnection of some pigments from the terminal emitters (Fig. S1). Second, Ho *et al*. estimated the ApcE2:ApcD3:ApcF:ApcD2+ApcD5:ApcB2 ratio of the subunits found in a bicylindrical FaRLiP-AP core isolated after only one sucrose gradient to be 2.0: 3.4: 4.2: 16.6: 21.4 by densitometric analysis of Coomassie-stained SDS-PAGE gels (39). Therefore, a single core cylinder should have the ratios 1.0: 1.7: 2.1: 8.3: 10.7. Based on the structure, if one were to assume only three (αβ)_3_ toroids in the complete FaRLiP-AP core, this ratio should be 1: 1: 1: 7: 8. If, however, one assumes that an (αβ)_3_ toroid was lost that comprises, *e.g.*, three ApcD5/ApcB2 protomers and an ApcC subunit, the ratio becomes 1: 1: 1: 10: 11 which is much closer to the initial estimation based on densitometry. Third, there is an ApcE2 loop that could not be modeled in the FaRLiP-AP core structure (residues 516–538), presumably due to its flexibility (Fig. S13). The analogous residues in ApcE1 of the AP core from PBS structures interact with the fourth (αβ)_3_ toroid and an additional ApcC molecule (22, 23); therefore, the unmodeled region of ApcE2 is probably flexible due to the loss of the fourth (αβ)_3_ toroid and ApcC. Finally, there would be complete correspondence between the FaRLiP-AP core cylinder and those of hemidiscoidal PBS if the former is missing one trimeric AP toroid (Fig. 2). Based on all these considerations, we conclude that the FaRLiP-AP core structure determined here is missing one (αβ)_3_ toroid with an accompanying ApcC subunit. The resulting complex would be structurally and compositionally analogous to the bottom core cylinders of hemidiscoidal PBS except that all of the subunits except ApcF and ApcC would be replaced by variants absorbing FRL (Fig. 2*B*).

The subunit composition of the missing (αβ)_3_ toroid is unknown but can be inferred by process of elimination from the information above. Based on the densitometric analysis reported previously (39), the following combinations could reasonably satisfy the ratio estimate: [ApcD5/ApcB2]_3_, [ApcD5/ApcB2]_2_+[ApcD5/ApcF], [ApcD5/ApcB2]_2_+[ApcD3/ApcB2], or [ApcD5/ApcB2] + [ApcD3/ApcB2] + [ApcD5/ApcF]. We think the latter two are unlikely because they would place ApcD3, which contains a red-shifted chromophore, further away from the probable interface with FRL-PSII and would therefore create a deleterious energy trap. They would also place ApcD3 in two different binding environments, which seems very unlikely. Likewise, placing ApcF anywhere in the structure except adjacent to ApcE2 in trimer 2 would place it in two structural contexts and would be unprecedented. The ApcC subunit that is maintained interacts only with ApcB2 and ApcE2. Therefore, we suggest that the missing fourth toroid should be [ApcD5/ApcB2]_3_-ApcC (Fig. 2*B*). This would be analogous to other PBS core substructures, in which the fourth (αβ)_3_ toroid that is furthest from the PSII interface with the terminal emitters comprises the bulk α- and β-subunits of AP, which in the case of the FaRLiP-AP cores are analogously ApcD5 and ApcB2, respectively.

**Figure 3:**
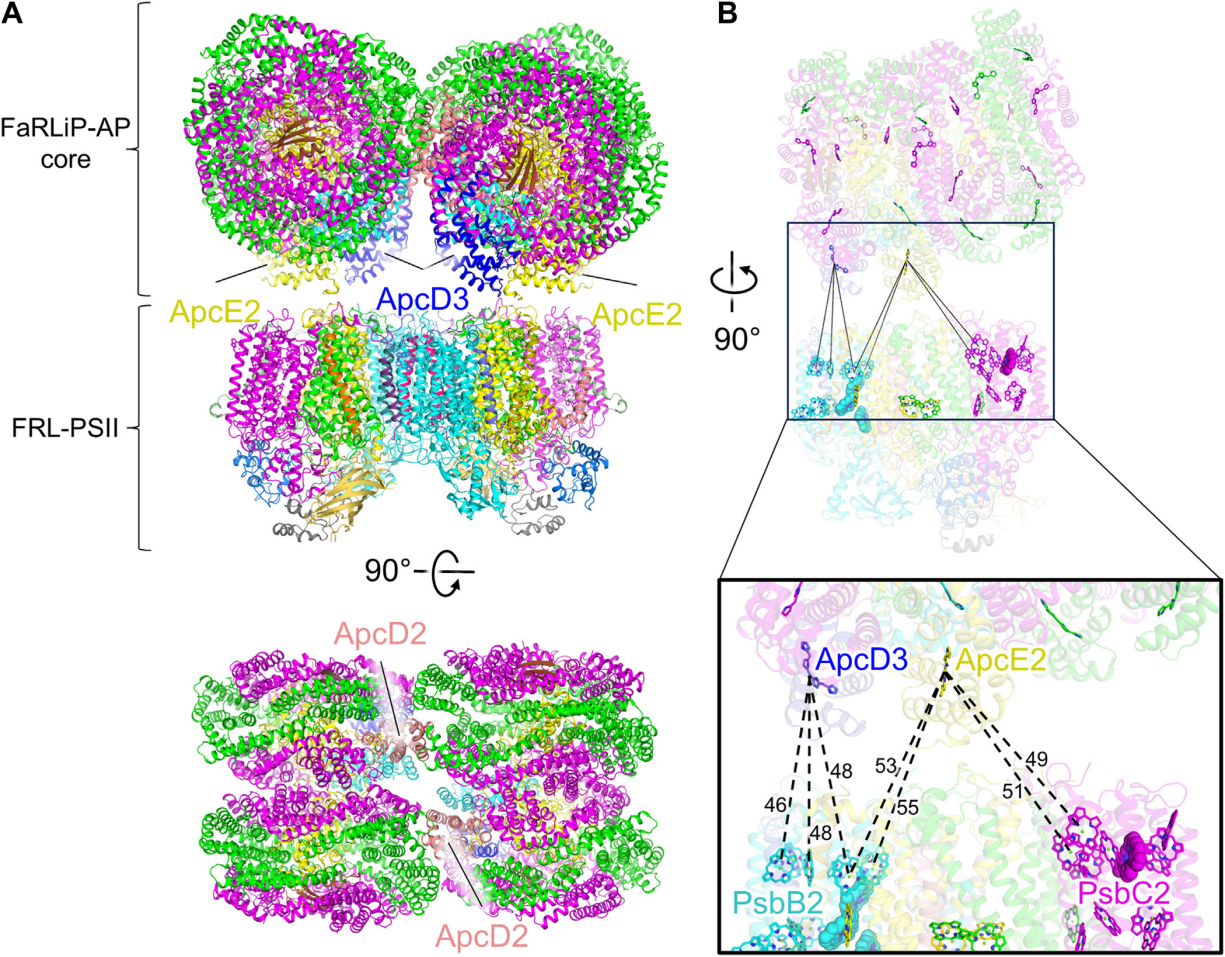
**Model of how a bicylindrical FaRLiP-AP core associates with FRL-absorbing PSII and distances of bilin pigments between complexes.***A*, two views of the bicylindrical FaRLiP-AP core in complex with FRL-absorbing PSII (FRL-PSII). Subunits with chromophores most likely to be *red* shifted are labeled. Subunits in the bicylindrical core are color coded identically to that shown in Figure 1. The FRL-PSII structure corresponds to PDB 8EQM. *B*, corresponding pigment arrangement. Tetrapyrrole substituents are hidden from pigments for clarity. Chl *f* molecules are shown in *sphere* representation. In the inset, several measurements are shown in units of Å to depict the approximate center-to-center distances between terminal emitters on the FaRLiP-AP core and Chl molecules in FRL-absorbing PSII. FaRLiP, far-red light photoacclimation; FRL, far-red light.

Based on all these considerations, we suggest that a bicylindrical FaRLiP-AP core with four (αβ)_3_ toroids orients onto FRL-PSII in the organization shown in Figure 3. This model was created by aligning the FaRLiP-AP core to one of the bottom two cylinders of the core substructure from the *Synechococcus* 7002 PBS (22) as shown in Figure 2*A*, copying the ApcD5, ApcB2, and ApcC subunits onto the subunits from *Synechococcus* 7002 corresponding to the ones missing in the FaRLiP-AP core structure. That complete FaRLiP-AP core was then copied onto the second bottom AP core cylinder from the *Synechococcus* 7002 PBS structure to make a bicylindrical core. Finally, the complete bicylindrical core model and a FRL-PSII dimer model (PDB 8EQM (38)) were fitted onto the low resolution PSII-PBS (hemidiscoidal) map generated from the species *Anabaena* sp. PCC 7120 (19).

Based on this model, the chromophores bound in ApcD3 and ApcE2 are the closest chromophores in the FaRLiP-AP core to the Chl molecules in FRL-PSII, the distances of which are all ∼50 Å (center-to-center); therefore, it is unclear based on distance measurements alone which energy transfer pathway might be dominant (if any). Likewise, it is unclear which of the FRL-PSII core antenna subunits, PsbB2 or PsbC2 or both, accepts the energy transferred from the FaRLiP-AP core. It is important to consider, however, that the low-resolution map used to fit the core and FRL-absorbing PSII likely contains a large amount of error, especially in the region of the PSII dimer that is poorly resolved (19). Future energy transfer studies might help to resolve energy transfer dynamics further, but recent spectroscopic studies suggested that energy transfer occurs through the Chl *f* molecule bound to the PsbC2 subunit (47). The model also suggests a possible functional role for ApcD2 beyond its role in absorbing FRL. ApcD2 forms important binding interactions between the two core cylinders in the bicylindrical core assembly (Fig. 3*A*). Lastly, it is noteworthy that ApcD3 is in the same position as ApcD in a typical hemidiscoidal PBS complex (Fig. 2*B*). ApcD has been shown to deliver energy to PSI (10, 48), which could also be the case for ApcD3 during FaRLiP.

### α-subunits and their chromophores

None of the subunit structures present within the FaRLiP-AP core have been determined previously except ApcC and ApcF. This provides an opportunity to examine the protein subunits and their chromophores, and to compare them to better studied examples of AP family variants. To make an initial overall comparison of α-subunits (excluding the N-terminal PBP domain of ApcE), we created a multiple sequence alignment with a diverse collection of α-subunit sequences (Fig. S14*A*), a multiple sequence alignment consisting solely of representative sequences for which structures are available (Fig. S15), and tables of sequence identity and root-mean square deviation (RMSD) of C_α_ atoms of those sequences and their corresponding structures (Tables S3 and S4). The α-subunit sequences exhibit relatively low sequence identity, from ∼35% to 50% (Table S3). ApcD3 is the least similar to the other AP family subunits in terms of both sequence identity (Table S3) and RMSD of C_α_ atoms (Table S4). Notably, ApcD3 is the only α-subunit in these comparisons with a noncovalently bound phycocyanobilin chromophore (12, 14, 40). AP family α-subunits found in FRL-absorbing complexes, which include ApcD2, ApcD3, ApcD4, and ApcD5, all contain a shorter BE loop compared to ApcA1 and ApcD1. The BE loop defines the pyrrole ring A protein environment of the phycocyanobilin chromophore (Figs. S14 and S15).

Because the phycocyanobilin-binding domain of ApcE is similar to a highly divergent α-subunit, we also generated a multiple sequence alignment of diverse ApcE sequences (Fig. S14*B*), and a sequence alignment containing a VL sequence (ApcE1) and the FRL sequence (ApcE2) for which structures are available (23) (Fig. S16). In both the ApcE1 structure (23) and the ApcE2 structure, a looping region could not be modeled; the corresponding sequences are highlighted gray in the sequence alignment shown in Fig. S16. This looping region is expected to interface with PSII, so we suggest that it is intrinsically unstructured and only becomes structured when bound to PSII. Unlike ApcE1, ApcE2 lacks the Cys residue that provides the thioether linkage to the phycocyanobilin chromophore (Figs. S14*B* and S16). The extended conjugated system in the chromophore should result in a red-shift of its absorbance (45, 46, 49).

**Figure 4:**
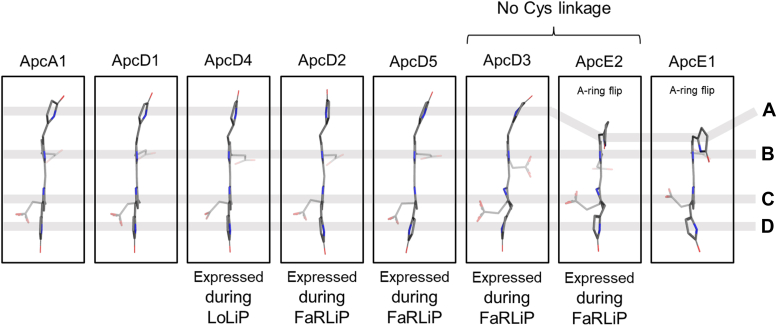
**Structures of selected α-chromophores.** The chromophore structures from *left* to *right* correspond to the following coordinates: ApcA1, PDB 4RMP (α subunit of AP); ApcD1, PDB 4PO5 (α-subunit of AP-B); ApcD4, PDB 8DDY (α-subunit of helical FRL-AP); ApcD2, reported herein; ApcD5, reported herein; ApcD3, reported herein; ApcE2, reported herein; ApcE1, PDB 7SC9. Note that the ApcD3 and ApcE2 chromophores are not covalently bound. From *top* to *bottom*, the pyrrole rings are A → B → C → D. FRL, far-red light.

**Figure 5:**
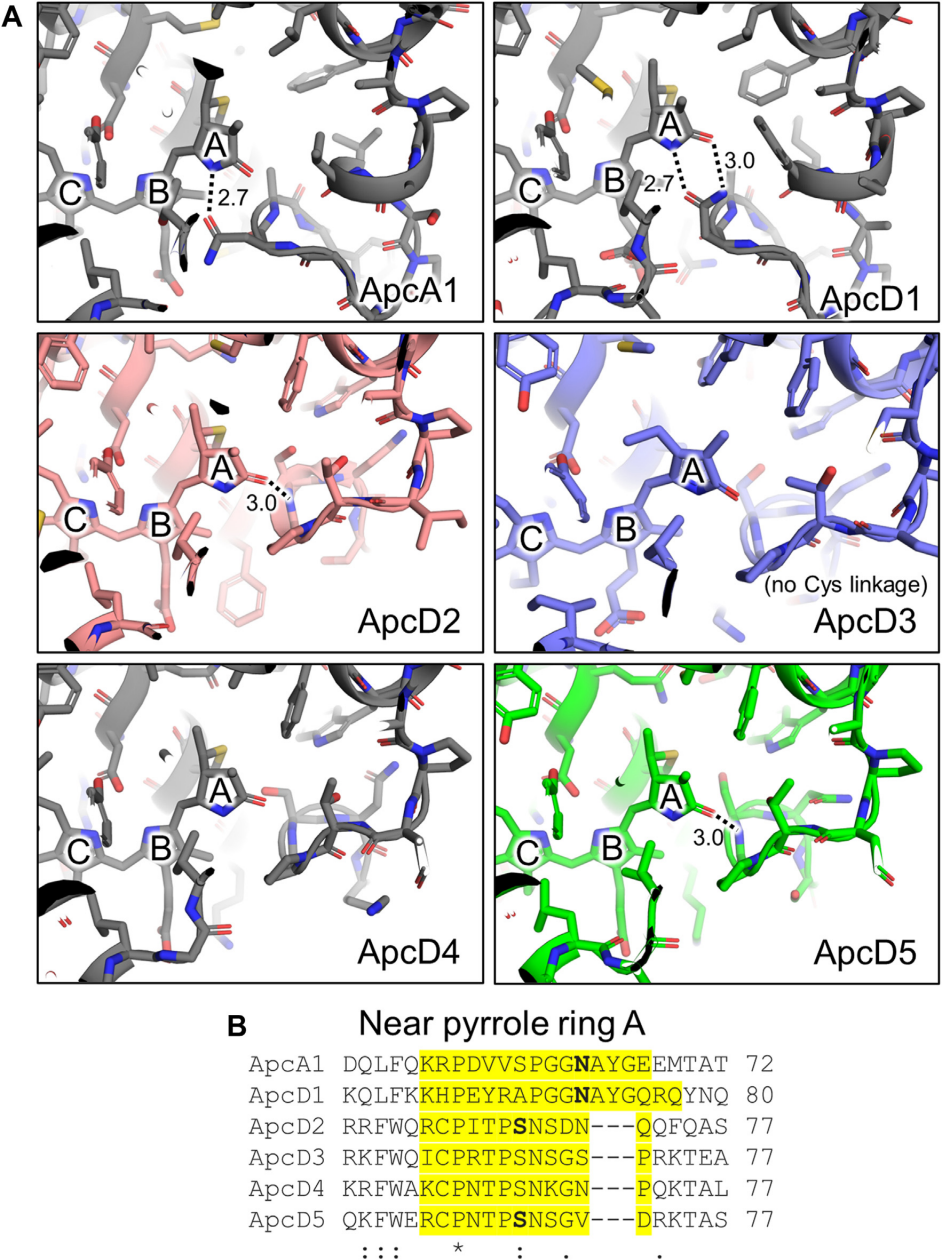
**Structures of selected α-chromophores within their protein environments.***A*, structures of AP family α-subunits. Those shown in colors were determined herein (ApcD2, ApcD3, and ApcD5). The structure of ApcA1 was taken from PDB 4RMP (AP). The structure of ApcD1 was taken from PDB 4PO5 (AP-B). The structure from ApcD4 was taken from PDB 8DDY (helical FRL-AP). For each panel, H-bonding interactions with pyrrole ring A of the chromophore are shown if they are present. *B*, partial sequence alignment showing the residues corresponding to the BE loop (highlighted). The residues involved in H-bonding interactions with the chromophore are in *bold*. Clustal Omega sequence conservation identifiers are shown below each position in the alignment. FRL, far-red light.

When the structures of AP-B and a red-shifted, helical FRL-AP were determined (14, 43, 50), it was observed that the pyrrole rings of the phycocyanobilins bound to the α-subunits (ApcD1 and ApcD4, respectively) were especially planar, which probably contributes to their red-shifted absorbance relative to the phycocyanobilin chromophore of ApcA1 in AP (14). Because the FaRLiP-AP core structure presented here includes the structures of four new FRL-absorbing, α-subunit chromophores (those bound to ApcD2, ApcD3, ApcD5, and the bilin-binding domain of ApcE2), we compared the coplanarity of the pyrrole rings in these bilins (Fig. 4), and we compared their protein environments with other available structures (Fig. 5). Note that although there are multiple ApcD5 subunits in the structure that occur in different structural contexts, we chose only to analyze the one exhibiting the highest local resolution. This seems appropriate because all ApcD5 chromophores exhibit identical local protein environments, which comprise residues from ApcD5 near pyrrole rings A-C and residues from ApcB2 near pyrrole ring D.

Pyrrole rings B and C of the α-subunit chromophores only interact with the Asp sidechain that is H-bonded to their amide nitrogen atoms, and this Asp is conserved in all PBPs. It makes pyrrole rings B and C coplanar such that they are structured essentially identically between different α-subunit bilins (Fig. 4). Pyrrole ring D interacts with the adjacent β-subunit of a second protomer but is especially challenging to compare between structures. In part, this is because, at least in ApcD1 and ApcD4, pyrrole ring D interacts with a water molecule which is not resolved in some structures, likely due to resolution limitations. It should be noted, however, that the β-subunit sequences in this region are identical (vertical lines in Fig. S14*C*), except that there is a Pro residue in ApcB3 that replaces Thr in ApcB1 and ApcB2. Even in the case of ApcD4, which pairs with ApcB3 in the helical FRL-AP structure, the Pro residue did not appear to influence substantially the interactions of pyrrole ring D compared to ApcB1 (50). Because the regions of ApcB1 and ApcB2 are identical in sequence, it seems unlikely that pyrrole ring D of α-subunit phycocyanobilins in their oligomeric states are much different from one another or contribute much to different absorbance properties. This is consistent with pyrrole ring D being nearly coplanar with rings B and C to a similar extent in the comparison of ApcA1, ApcD1, ApcD2, ApcD3, ApcD4, and ApcD5 (Fig. 4). An exception is the Trp sidechain that interacts with pyrrole ring D of all the α-subunit chromophores except ApcA1. This Trp was shown to red-shift ApcD1 compared to ApcA1 in which it is Tyr instead (43).

The differences in the pyrrole ring A positions and their protein environments are more striking. This is consistent with the structural interpretations of AP-B and the helical FRL-AP (14, 43, 50), where it was suggested that red-shifting of the absorbance was determined by the extent to which pyrrole ring A is coplanar with the rest of the chromophore (*i.e.*, more coplanar = a more red-shifted spectrum) (14, 43, 50). The phycocyanobilin bound to ApcD5 is the least coplanar of the α-subunit chromophores found in the FaRLiP-AP core structure (Fig. 4), being more similar to the chromophores bound to ApcA1 or ApcD1. This is surprising because ApcD5 contains the shorter BE loop that is conserved in all FaRLiP-AP sequences (ApcD5, ApcD4, ApcD3, and ApcD2) (Figs. 5*B*, S14*A*, and S15). Like ApcA1 and ApcD1, the carbonyl moiety of pyrrole ring A of the phycocyanobilin bound to ApcD5 accepts an H-bond from the protein environment. Whereas an Asn sidechain provides this interaction in ApcA1 and ApcD1, it is instead provided by the backbone amide nitrogen atom of a Ser residue conserved in the red-shifted AP variants that contain the shorter BE loop (Fig. 5). Despite the shorter BE loop, these observations suggest that the chromophores bound to ApcD5 subunits probably contribute the most blue-shifted character to the overall absorbance of the FaRLiP-AP core structure (Fig. S1). It should be noted, however, that the interpretations of AP-B and helical FRL-AP structures suggested that the shorter BE loop might sterically constrain the phycocyanobilin of the α-subunit, thereby influencing its absorbance properties (14, 51). This may also be the case for the phycocyanobilin bound to ApcD5.

Like the phycocyanobilin bound to ApcD4 determined from the helical FRL-AP structure (14, 50), pyrrole ring A of the phycocyanobilin bound to ApcD2 in the FaRLiP-AP core structure exhibits high coplanarity with the other rings in the chromophore (Fig. 4), probably causing it to contribute a similar extent of red-shifting to the overall absorbance profile of the FaRLiP-AP core (Fig. S1). Unlike the chromophore bound to ApcD4, however, the carbonyl oxygen atom of the A ring of the ApcD2 phycocyanobilin accepts an H-bond from the protein environment: the backbone amide nitrogen of the conserved Ser residue to which pyrrole ring A of the ApcD5 bilin is also H-bonded. This might red-shift the absorbance of ApcD2. Considering the position of ApcD2 in our model (Fig. 3*A*), the ApcD2 chromophore may be involved in energy transfer between the two halves of the bicylindrical core. Distances between chromophores near the interface of the two cylinders based on the inferred model are shown in Fig. S17.

Based on our model, the two subunits whose phycocyanobilin chromophores are probably the closest to the Chls in FRL-PSII *in vivo* are ApcD3 and ApcE2 (Fig. 3). This arrangement makes sense because the phycocyanobilins of these subunits lack Cys thioether linkages to the protein, which red-shifts their absorbance due to the extension of the conjugation system in these chromophores. Surprisingly, compared to other α-subunit bilins, the pyrrole rings of the ApcD3 phycocyanobilin are not highly coplanar (Fig. 4). It is also noteworthy that this phycocyanobilin does not exhibit H-bonding interactions to its pyrrole ring A, which is similar only to the chromophore bound to ApcD4 in the helical FRL-AP structure (Fig. 5*A*). ApcE2, like ApcE1, exhibits a flipped pyrrole ring A (Figs. 4 and S18). Aside from the lack of the thioether linkage of the phycocyanobilin bound to ApcE2, the protein environments for the phycocyanobilins bound to ApcE1 and ApcE2 are similar. However, in ApcE2 the carbonyl oxygen atom of pyrrole ring A is within H-bonding distance of a Phe sidechain (Phe172, Fig. S18), which creates a weak CH-O type H-bonding interaction that might influence the spectrum of the chromophore. While this residue is also conserved in ApcE1, it is not within H-bonding distance of the A-ring keto oxygen. In addition to this H-bonding interaction difference, the phycocyanobilin of ApcE2 appears to be more planar than that for ApcE1 (Fig. 4), which should influence its absorbance properties. Finally, the chloride ion identified in the FaRLiP-AP core structure (Fig. 1) is closest to the chromophore of ApcE2, ∼28 Å, which might also alter its role in energy transfer.

### β-subunits and their chromophores

To characterize the differences in β-subunits, we initially created a multiple sequence alignment from diverse β-subunit sequences (Fig. S14*C*), a multiple sequence alignment consisting only of representative sequences for which structures are available (Fig. S19), and tables of sequence identity and root-mean square deviation (RMSD) of C_α_ atoms of those sequences and their corresponding structures (Tables S5 and S6). These included ApcB1, ApcB2, ApcB3, and ApcF. Comparing representative sequences, the sequence identity is generally higher than that for the α-subunits, ∼45 to 70% (Table S5). ApcF is least similar to the other β-subunit sequences, with only ∼45 to 50% identities. Structurally, the β-subunits are generally more similar to one another than was the case for α-subunits, with the exception of ApcF, which is about as dissimilar to other β-subunits as ApcD3 is to other α-subunits. This is reasonable because both ApcF and ApcD3 have specialized structural functions.

**Figure 6:**
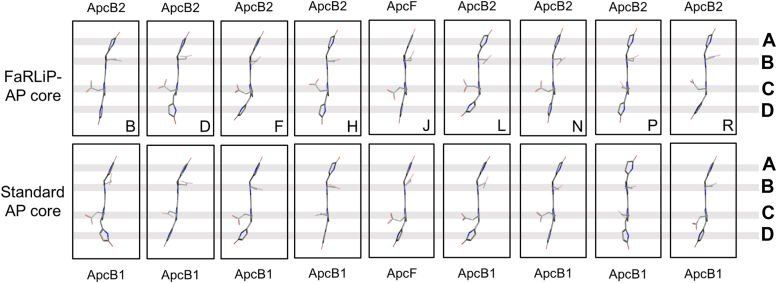
**Structures of β-chromophores in the FaRLiP-AP core compared to a VL-absorbing AP core from *Synechocystis* 6803.** Comparison of β-subunit bilins within the FaRLiP-AP core structure (*top row*) and to the corresponding bilins within the structure of an AP core from *Synechocystis* 6803 that does not absorb FRL (PDB 7SC9). The letter below each bilin in the FaRLiP-AP core structure is the chain letter in the deposited coordinates. From *top* to *bottom*, the pyrrole rings are A → B → C → D. FaRLiP, far-red light photoacclimation; FRL, far-red light.

Although there are eight ApcB2 subunits in the FaRLiP-AP core structure, each chromophore is in a unique structural environment, which is unlike the case for ApcD5 subunits, the chromophores of which each occur in identical environments. Whereas the protein environments of pyrrole rings A, B, and C of all the β-subunit chromophores (including the β-subunit ApcF) comprise residues from the same β-subunit to which it is covalently bound, pyrrole ring D is in a different environment in every case, typically interacting with different parts of ApcE2 (Table S7). This is nicely visualized in a comparison of β-subunit chromophores (Fig. 6) where pyrrole rings A-C are similar to one another, but there is much more variation in the conformation of pyrrole ring D.

To determine whether β-subunits contribute to the red-shifted spectrum of FaRLiP-AP cores, we compared the chromophore structures of the FaRLiP-AP core β-subunits to the analogous chromophores in the VL-absorbing PBS structure from *Synechocystis* sp. PCC 6803 (hereafter *Synechocystis* 6803) (23) (Fig. 6). The comparison shows that nearly all the chromophores are structured similarly between those found in the FaRLiP-AP core and the corresponding phycocyanobilin found in a typical, VL-absorbing AP core from a hemidiscoidal PBS. Two exceptions are those found in chains B and D of the FaRLiP-AP core structure, for which the D rings appear to be oriented differently between ApcB2 and ApcB1. These are found in the first trimer of the FaRLiP-AP core (Figs. 1*A* and 2*B*), and their pyrrole rings D each interact primarily with different parts of ApcC (Table S6). Because ApcC is not a FRL-specific subunit, these differences in pyrrole ring D orientations are possibly due to species-specific differences in the protein sequences rather than differences related to FRL-specific sequence differences.

To test this hypothesis, we compared the protein environments of the respective pyrrole rings D in more detail. In the case of the ApcB2 bilin of chain B, there is an ApcB2-Phe87 residue that appears to push pyrrole ring D into the plane of the rest of the bilin (Fig. S20). This is unlike other ApcB2 subunits in the FaRLiP-AP core complex due to unique ApcC residues in this region (to reiterate, all ApcB2 pyrrole ring D environments, which includes the environment of ApcB2-Phe87, are unique). ApcB2-Phe87 is present only in the *Synechococcus* 7335 ApcB2 sequence in our multiple sequence alignment, being Tyr in most other sequences of ApcB1 and ApcB2 (Fig. S14*C*), including the VL-absorbing AP core from *Synechocystis* 6803. Thus, although this ApcB2-Phe87 interaction with pyrrole ring D might red-shift the absorbance properties of the phycocyanobilin in the FaRLiP-AP core, it is probably species specific and due primarily to differences in ApcC that is not a FRL-specific subunit. The situation is similar with the ApcB2 bilin of chain D in the FaRLiP-AP core: the only difference in the protein environment is an ApcC-Gln44 residue in the FaRLiP-AP core that is instead Met in the *Synechocystis* 6803 structure. These observations suggest that the differences in the ApcB2 pyrrole ring D orientations of chains B and D compared to the corresponding chromophores in a standard VL-absorbing AP core are not related to FRL-specific sequence differences but are instead due to sequence variation among organisms.

## Discussion

The arrangement of subunits in the FaRLiP-AP core provides a structural basis for recent spectroscopic measurements from Ho *et al*. (52). In that work, time-resolved fluorescence spectra were recorded of FaRLiP-AP bicylindrical cores excited at 600 nm. It was found that energy migrates from a higher energy pool emitting at ∼720 nm to a lower energy terminal emitter at 730 nm (ApcD3 and/or ApcE2). This is consistent with the structural model (Fig. 3), in which the terminal emitters are co-located in the hexamer whose chromophores would be closest to the Chls in FRL-PSII. Thus, the structure can be viewed as two distinct modules: a sensitizer module comprising an ApcD5/ApcB2 hexamer that emits at ∼720 nm, and an emitter module containing lower energy chromophores emitting at ∼730 nm. Structurally, this arrangement mirrors the bottom core cylinders of typical hemidiscoidal PBS (Fig. 2*B*). AP-B and the chromophore-binding domain of ApcE1 are found in one hexamer, whose chromophores are the closest to the Chl molecules found in PSII, and the other is further away due to the overall tilting of the core cylinder. The similarities in this arrangement between FRL and VL imply that, whatever the route for energy transfer is in one case, it is probably similar or identical in the other. The distal position of the fourth trimer, and the ease with which it is lost during isolation, might explain why some cyanobacteria and red algae have lost this AP core trimer (20, 21, 24).

A marked difference between the hemidiscoidal PBS and FaRLiP-AP core is the number of chromophores that they add as antenna. Whereas hemidiscoidal PBS contains hundreds of chromophores, a FaRLiP bicylindrical core contains only 48. However, FaRLiP is specifically induced to access a much narrower range of light wavelengths, so perhaps more chromophores are unnecessary. Additionally, FaRLiP is facultative, so the ability to stop producing FaRLiP-AP cores and quickly return to producing hemidiscoidal PBS is probably advantageous, which may explain why larger complexes are not produced during FaRLiP. Furthermore, FaRLiP uses fewer resources by making fewer protein subunits, which conserves both energy and nutrient resources. Although we describe the FaRLiP-AP bicylindrical cores as separate entities from the photosystems, it is important to be aware of their intimate dependance upon one another. Mutants lacking any of the FRL-specific FaRLiP-AP subunits do not grow in FRL (41). Thus, the FaRLiP-AP cores are essential components of the molecular processes that allow for photoautotrophic growth under FRL. In fact, it is possible that the FaRLiP-AP cores evolved before Chl *f* or Chl *d* were produced and bound to the photosystems. These adaptations might have followed changes in the antenna that were introduced to improve the efficiency of using FRL (53, 54).

We wish to highlight an observation about the β-subunits when comparing the helical (14) FRL-absorbing AP complexes expressed during low-light photoacclimation and the core cylinder AP core complexes expressed during FaRLiP. In the absorbance spectra of AP family oligomers, two peaks are observed: one of which arises primarily from the α-subunit chromophores (the lower energy peak) and the other which arises primarily from the β-subunit chromophores (the higher energy peak). This is exemplified by the comparison of absorbance spectra from the two FRL-absorbing AP complexes shown in Fig. S2. In the case of the helical FRL-AP, the peak primarily arising from the β-subunit chromophores has its absorbance maximum at 621 nm, but for the FaRLiP-AP core structure described here, the absorbance band primarily arising from the β-subunit chromophores exhibits a maximum at 650 nm. The basis for this difference can be attributed to the protein environments of pyrrole ring D of the β-subunit chromophores. In the case of the helical FRL-AP complexes, pyrrole ring D of the β-subunit chromophores apparently is largely devoid of protein interactions (*i.e.*, there are no linker protein interactions equivalent to those provided by ApcC and ApcE in isolated complexes), and this is the case for every β-subunit chromophore in the helix, which results in the 621 nm peak maximum (Fig. S2). In the FaRLiP-AP core complex, however, pyrrole ring D for every β-subunit chromophore has an interaction with a linker protein, and that interaction is unique for each β-subunit, resulting in a bathochromic shift of the absorbance maximum to 650 nm. Therefore, it can be deduced that the linker protein interactions with pyrrole ring D of β-subunit chromophores induce a red-shift of their absorbance of ∼30 nm. Whereas α-subunit chromophores are red-shifted by coplanarity of pyrrole ring A with the other pyrrole rings or a lack of thioether bonding and give rise to similar maxima in the two FRL-absorbing AP complexes (∼710 nm) (51), β-subunit chromophores are more red-shifted in the FaRLiP-AP core complexes due to linker protein interactions with pyrrole ring D.

In addition to the influence of the protein environments on the chromophores of the β-subunits that gives rise to the absorbance maximum at 650 nm of FaRLiP-AP cores, it is also possible that the ApcD3 subunit contributes to this absorbance peak. By recombinant expression of ApcD3 and ApcB2, Soulier *et al*. showed that the isolated protomers, some of which may form higher oligomeric states, exhibited absorbance maxima at 701 and 660 nm, with a strong shoulder at ∼615 nm (40). Other protomers containing ApcB2 (*e.g.*, ApcD5-ApcB2) exhibited maxima at ∼705 and 615 nm, suggesting that ApcB2 contributes mostly absorbance ∼615 nm in isolated protomers. This suggests that, at least in isolated protomers, the phycocyanobilin chromophore on ApcD3 contributes some absorbance at ∼660 nm. There is one ApcD3-ApcB2 protomer in the FaRLiP-AP core complex, so it is reasonable to suggest that part of the absorbance around 650 nm (Fig. S2) arises from the chromophore bound to ApcD3.

Our structural elucidation of an AP core complex expressed during FaRLiP and our inferred model provide excellent insights into the molecular basis of FaRLiP, especially in accompaniment with the available photosystem structures that are also expressed during FaRLiP. It is noteworthy, however, that numerous questions remain. First, how do FaRLiP-AP cores interact with one another? The presence of ApcD2, which contains a highly planar and therefore red-shifted chromophore, at the interface where the two core cylinders would be expected to interact implies that energy transfer can probably occur between cores. Another important question pertains both to FaRLiP-AP cores and VL-absorbing hemidiscoidal PBS: How do these complexes interact with PSII? This is especially interesting for the complexes expressed during FaRLiP because energy is probably transferred from the FaRLiP-AP core through a Chl *f* molecule found in FRL-PSII. The only direct structural data on a cyanobacterial PBS-PSII complex is very low resolution (19), so the measurements we derive in Figure 3 should be interpreted cautiously. Although chemical crosslinking data on cyanobacterial PBS-PSII exist (55), it does not directly inform the distances between pigments that are required to determine how energy is transferred between the complexes. *In situ* structural data on how the hemiellipsoidal PBS of red algae interacts with the photosystems were recently reported (25), but the arrangement of the PBS and PSII complexes in red algae is much different than is thought to be the case for the cyanobacterial PBS-PSII complex (19). Thus, one could imagine that future tomographic cryo-EM data might be used to determine how cyanobacterial PBS interact with PSII, for both VL and FaRLiP complexes.

## Experimental procedures

### Cell cultivation and core complex isolation

Liquid cultures of *Synechococcus* 7335 cells were grown in ASNIII medium in FRL sparged with 1% (v/v) CO_2_ in air as previously described (37, 39). FRL-acclimated cells were harvested by centrifugation at 5010*g* for 10 min. The cell pellets (∼28 g, wet weight) were washed once in 0.75 M K-phosphate buffer, pH 7.0, resuspended in ∼100 ml of 0.75 M K-phosphate buffer, and disrupted by three passages through a chilled French pressure cell at 138 MPa. Triton X-100 (2% w/v, final concentration) was added to the lysed cell suspension, which was gently stirred at room temperature until the solution became homogenous (∼20 min). Unbroken cells and large cell debris were removed by centrifugation at 17,210*g* for 20 min. Aliquots (5.5 ml) of the PBP-containing supernatant fraction were loaded onto linear sucrose gradients (24 ml) made with 0.4 M to 2.0 M sucrose in 0.75 M K-phosphate buffer, pH 7.0. The resulting gradients were centrifuged at 125,800*g* for 18 h at 20 °C. The middle aqua-colored band containing the FRL-absorbing cores was collected and dialyzed against 0.75 M K-phosphate, pH 7.0, changing the buffer twice. The dialyzed solution was subjected to centrifugation at 33,210*g* for 20 min to remove precipitated, contaminating Chl-containing complexes, and aliquots were removed for absorbance and fluorescence spectroscopy. The dialyzed solution was concentrated using Millipore Ultra 15-mL centrifugal filters (30 kDa cutoff). Aliquots (4 ml) of the concentrated solution containing the FRL-absorbing core complexes were loaded on linear sucrose gradients (24 ml) made with 0.4 M and 1.5 M sucrose solutions prepared with 0.75 M K-phosphate buffer, pH 7.0. These second-round gradients were centrifuged at 125,800*g* for 18 h at 20 °C, and a typical gradient is shown in Fig. S4. The upper pinkish-blue band containing contaminating phycoerythrin and phycocyanin was discarded. The broad, aqua-colored band containing PBPs absorbing at 710 nm was collected and dialyzed against 0.75 M K-phosphate buffer, pH 7.0, to remove the sucrose. This fraction was concentrated as described above on Millipore Ultra 15-mL centrifugal filters, and aliquots were removed for absorbance and fluorescence emission spectroscopy, SDS-PAGE, and chymotryptic peptide fingerprinting by mass spectrometry as previously described (37, 39). Size-exclusion chromatography (Fig. S4*C*) was performed using a HiLoad Superdex 200 16/600 column equilibrated with 100 mM K-phosphate buffer, pH 7.0 with 10 mM ethylenediaminetetraacetic acid. The collected fractions (Fig. S4*C*) were concentrated using a Millipore Ultra 15-mL centrifugal filter and the sample was stored at 4 °C until required for cryo-EM analysis.

### Cryo-EM sample preparation and data collection

To prepare the cryo-EM sample, 3 μl of the protein solution at ∼2.15 mg/ml (based on A_280_) was applied to a glow-discharged (60 s at 25 mA, TedPella PELCO easiGlow) C-flat 2/1 Au 300-mesh (Electron Microscopy Sciences) microscopy grid in a Vitrobot Mark IV system (Thermo Fisher Scientific). The grid was blotted for 3 s and was plunge-frozen into liquid ethane with the Vitrobot set to 4 °C and 100% humidity. It was then transferred to liquid nitrogen until imaging. The grid was imaged using an FEI Titan Krios transmission electron microscope (300 kV) equipped with a Gatan K3 direct electron detector. Nominal magnification was set to 1,050,00 × with a super-resolution pixel size of 0.4125 Å, the defocus range was set to −0.8 to −2.2 μm, and the GIF slit size was 15 eV. Data were collected using a dose rate of 15 e^−^ physical pixel^−1^ s^−1^. For each micrograph movie, the total exposure time was 2.23 s delivering a total dose of 50 e^−^ (Å)^−2^. Eleven thousand sixty seven micrograph movies with 40 images per stack were collected using EPU (Thermo Fisher Scientific).

### Cryo-EM data processing and modeling

Figs. S6 and S10 show the data processing workflows for Rubisco and the FaRLiP-AP core, respectively. All data processing was performed using RELION 3.1 (56). Frame alignment and correction and dose-weighting were performed using MotionCor2 (57), and Ctffind-4.1.13 (58) was used to estimate the contrast transfer function. To create initial classes for template-based Autopicking, ∼1000 particles were selected manually. Based on the resulting 2D classes, the initial Autopicking selected 6,708,708 particle coordinates. The first round of 2D classification showed two types of particles, and both types were selected (1,165,500 particles in total) for continued processing. Upon the second round of 2D classification, either only Rubisco (Fig. S6, 108,079 particles) or only FaRLiP-AP core particles (Fig. S10, 931,259 particles) were selected. For the Rubisco workflow, one round of 3D classification led to the isolation of 57,918 particles in the final particle selection. Repeated rounds of CTF refinement and Polishing led to a final 3D reconstruction at a resolution of 2.35 Å with a B-factor of −57.4 Å^2^ using D4 symmetry (Table S2 and Fig. S7). For the FaRLiP-AP core workflow, four rounds of 3D classification led to the isolation of 113,162 particles in the final particle selection. Repeated rounds of CTF refinement and Polishing led to a final 3D reconstruction at a resolution of 2.78 Å with a B-factor of −58.9 Å^2^ without symmetry (*i.e.*, C1 symmetry) (Table S2 and Fig. S11). Resolutions were determined based on the Gold standard Fourier Shell Correlation cutoff criterion of 0.143 (56, 59). Note that in the FaRLiP-AP core map, particles exhibit preferential orientation. This can be observed in the 2D classes (Fig. S10*A*) and the angular distribution (Fig. S11*C*). As a result, the map exhibits a directional elongation that can be observed in the local resolution map shown in Fig. S11*B*.

For the Rubisco structure subunits, the starting models were homology models of the large and small subunits from PDB 2V63 (60) and PDB 1RSC (61), respectively, created using SwissModel (62). For the FaRLiP-AP core structure, homology models were made of ApcB2, ApcC, ApcE2, and ApcF from corresponding subunits in PDB 7SC7 as templates, and homology models were made of ApcD2, ApcD3, and ApcD5 from template ApcD4 in PDB 8DDY. These were also created using SwissModel (62). Initial homology model fitting into the maps was performed using UCSF Chimera (63). Different AP α- and β-subunits were assigned in the structure by fitting each of the potential FaRLiP-AP subunits into the map and determining which fit the map best based on the sequence. Homology models were manually edited into the sharpened and unsharpened maps using Coot version 0.8.6.1 (64). Rounds of automated refinement were performed using Phenix *real_space_refine* (65). The final model statistics for both structures are shown in Table S2. In the FaRLiP-AP core structure, peripheral regions of the map were challenging to model due to the preferential orientation issue described above. In some cases, only the main chain could be modeled, and sidechains were therefore removed. As a result, the map-to-model Fourier Shell Correlation is lower resolution than the map-to-model Fourier Shell Correlation by 0.3 Å (Fig. S11). Higher resolution regions such as the chromophore-binding sites, which are buried closer to the center of the map, exhibit much less map elongation caused by the preferential orientation, and their positions are therefore modeled with greater confidence.

## Data availability

The FaRLiP-AP and Rubisco coordinates are deposited in the Protein Data Bank under accession code 8UHE and 8UHI, respectively. The corresponding maps are deposited in the Electron microscopy Data Bank under accession codes EMD-42278 and EMD-42281, respectively.

## Supporting information

Supplemental information can be found online at https://doi.org/10.1016/j.jbc.2023.105590 (42, 66, 67).

## Conflict of interest

The authors declare that they have no conflicts of interest with the contents of this article.
