# Supplementary material for: Structure of the antenna complex expressed during far-red light photoacclimation in Synechococcus sp. PCC 7335
Source: J Biol Chem. 2023 Dec 22;300(2):105590. doi: 10.1016/j.jbc.2023.105590 (PMC10810746; doi:10.1016/j.jbc.2023.105590)
Supplement: Supporting information [file mmc1.docx]

Supplementary Information for

**Structure of the antenna complex expressed during far-red light photoacclimation in *Synechooccus* sp. PCC 7335**

**Christopher J. Gisriel^1^, Gaozhong Shen^2^, Gary W. Brudvig^1,3^, Donald A. Bryant^2,^***

^1^Department of Chemistry, Yale University, New Haven, CT 06520, USA; ^2^Department of Biochemistry and Molecular Biology, The Pennsylvania State University, University Park, PA 16802, USA; ^3^Department of Molecular Biophysics and Biochemistry, Yale University, New Haven, CT 06520, USA

*Correspondence: [dab14@psu.edu](mailto:dab14@psu.edu)

**Text S1.** Description of the *Synechcoccus* 7335 Rubisco structure and comparison to Rubisco from *Synechococcus* 6301.

**Figure S1.** Comparison of spectra of FaRLiP-AP core complexes after one or two sucrose gradient separations.

**Figure S2.** Comparison of the absorbance spectrum of the helical FRL-AP (ApcD4-ApcB3) from *Thermostichus* sp. (formerly *Synechococcus* sp.) produced during LoLiP with the AP core complexes from *Synechococcus* 7335 produced during FaRLiP.

**Figure S3.** SDS-PAGE analysis of the FaRLiP-AP core complex isolated from cells of *Synechococcus* 7335 grown in FRL.

**Figure S4.** Preparation of FaRLiP-AP core sample for cryo-EM.

**Figure S5.** Screening micrograph image of FaRLiP-AP core complexes after size exclusion chromatography.

**Figure S6.** Cryo-EM data processing for Rubisco.

**Figure S7.** Cryo-EM data processing for Rubisco.

**Figure S8.** Structural comparison of Rubisco from *Synechococcus* 7335 and Rubisco from *Synechococcus* 6301.

**Figure S9.** Cryo-EM structure and map of selected regions from *Synechococcus* 7335 Rubisco determined herein.

**Figure S10.** Cryo-EM data processing for FaRLiP-AP cores.

**Figure S11.** Cryo-EM data processing for FaRLiP-AP cores.

**Figure S12.** Anion tentatively modeled as a chloride.

**Figure S13.** Stereo views of chromophores in sharpened and unsharpened maps.

**Figure S14.** Partial sequence alignment of the ApcE region that interacts with a fourth trimer and ApcC in PBS structures.

**Figure S15.** Multiple sequence alignments of α-subunits and β-subunits for representatives of the AP family.

**Figure S16.** Multiple sequence alignment of selected AP family α-subunits for which structures are available.

**Figure S17.** Sequence alignment comparing ApcE2 from *Synechococcus* 7335 and ApcE1 from *Synechocystis* 6803.

**Figure S18.** Distances between chromophores near the interface of the two cylinders of the FaRLiP-AP core complex.

**Figure S19.** Comparison of phycocyanobilin chromophores bound to ApcE2 and ApcE1.

**Figure S20.** Sequence alignment of selected AP family β-subunits for which structures are available.

**Figure S21.** Protein environment of ApcB2 chain B pyrrole ring D compared to the corresponding environment in ApcB1 pyrrole ring D in the VL-absorbing PBS structure from *Synechocystis* sp. PCC 6803.

**Table S1.** Selected phycobiliproteins identified by chymotryptic peptide fingerprinting by MS/MS spectrometry of the isolated FaRLiP-AP core fraction analyzed by cryo-EM in this study.

**Table S2.** Cryo-EM data statistics for the FaRLiP-AP core and Rubisco complexes.

**Table S3.** Sequence identity comparing selected AP family α-subunits.

**Table S4.** C_α_ superpositions comparing selected AP family α-subunits.

**Table S5.** Sequence identity comparing selected AP family β-subunits.

**Table S6.** C_α_ superpositions comparing selected AP family β-subunits.

**Table S7.** Linker protein interactions with pyrrole ring D of the phycocyanobilins attached to β-subunits.

Supplemental Text

**Text S1. Description of the *Synechcoccus* 7335 Rubisco structure and comparison to Rubisco from *Synechococcus* 6301.**

Ribulose-1,5-bisphosphate carboxylase/oxygenase, or Rubisco, is the first enzyme involved in the dark reactions of photosynthesis, and is responsible for converting CO_2_ into organic molecules for energy storage. Rubisco is one of the most abundant enzymes on Earth and was apparently abundant in our protein isolation of the FaRLiP-AP core complexes as well. The fact that Rubisco was present in our protein isolation suggest that its hydrophilicity, size, charge and density are similar those of the FaRLiP-AP complex. Upon the second round of 2D classification, classes corresponding to Rubisco were selected for continued processing (~100,000 particles, **Fig. S6**). Compared to the number of FaRLiP-AP core particles selected after the second round of 2D classification (~900,000, **Fig. S10**), we estimate that ~1/10th of the particles in the initial data set corresponded to Rubisco. Despite a much lower number of particles in the Rubisco data set, its D4 symmetry allowed for its structural determination to 2.35 Å global resolution with local resolutions spanning 1.95 to 3.15 Å (**Fig. S7**).

Rubisco identified in this protein isolation is form I, the most common form among cyanobacteria, comprising eight large and eight small subunits (L_8_S_8_) with local 422 symmetry (**Fig. S8A**). It is activated, with a carbamylated Lys, a bound ribulose-1,5-bisphosphate (RuBP) molecule, and a Mg^2+^ ion in its active site (**Fig. S9**). The structure is essentially identical to other similar Rubisco structures such as that of *Synechococcus elongatus* PCC 6301 (hereafter *Synechococcys* 6301, PDB 1RBL) (1). To provide a brief comparison, we visualized sequence conservation of *Synechococcus* 7335 Rubisco with *Synechococcus* 6301 Rubisco by showing the structure of the former colored by sequence conservation (**Fig. S8B**). Most of the sequence differences are in peripheral looping regions away from the active site, and there are more differences in the small subunit than in the large (large subunit sequences are 85.17% identical and small subunits are 66.06% identical). This is consistent with the fact that there is more variation among small Rubisco subunits (2), and that that the deletion of small subunits from Rubisco in *Synechococcus* sp. still preserves some carboxylase activity (3). We also calculated the electrostatic surfaces of the two Rubisco molecules (**Fig. S8C**). In a view of the side facing the active site (left panel of **Fig. 8C**), the electrostatic surfaces appear quite similar, but in a view of the side facing the small subunits, there is much more variation in the surface electrostatics, again exemplifying the lower conservation in Rubisco small subunits.

**References for Text S1**

1. Newman, J., Brändén, C.-I., and Jones, T. A. (1993) Structure determination and refinement of ribulose 1,5-bisphosphate carboxylase/oxygenase from *Synechococcus* PCC6301. *Acta Crystallogr., Sect. D-Biol. Crystallogr*. **49**, 548–560
2. Andersson, I., and Backlund, A. (2008) Structure and function of Rubisco. *Plant Physiol. Biochem.* **46**, 275–291
3. Andrews, T. J. (1988) Catalysis by cyanobacterial ribulose-bisphosphate carboxylase large subunits in the complete absence of small subunits. *J. Biol. Chem*. **263**, 12213–12219

Supplemental Figures


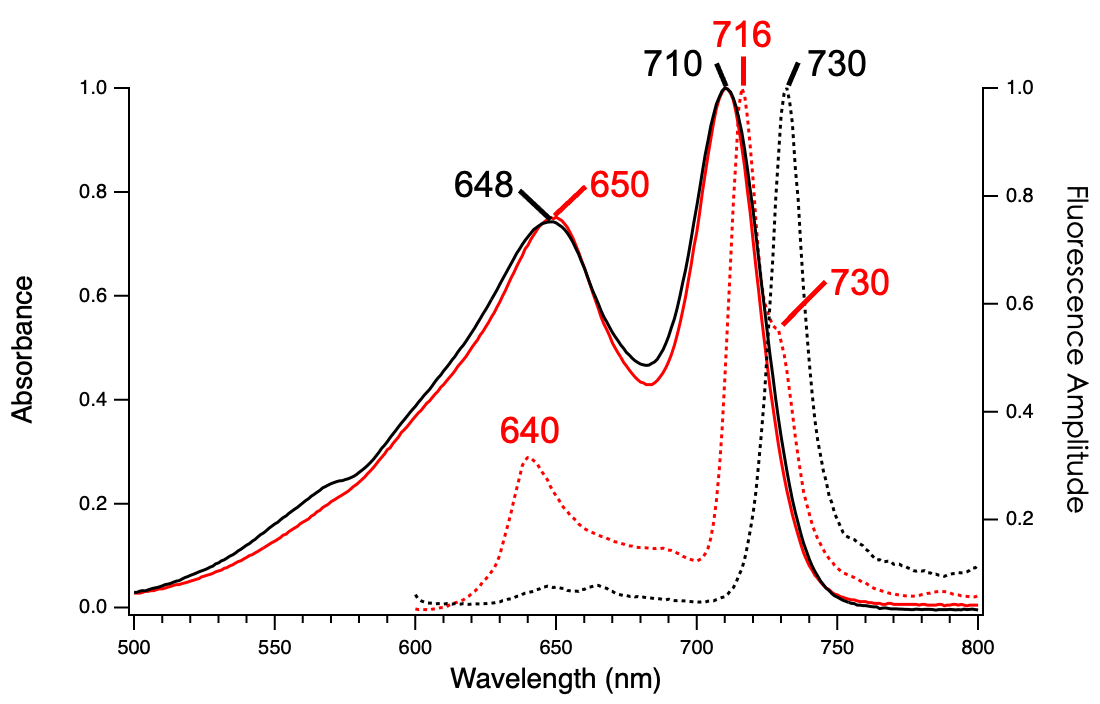


**Figure S1. Comparison of spectra of FaRLiP-AP core complexes after one or two sucrose gradient separations.** Spectra of FaRLiP-AP cores upon the first (black) and second (red) sucrose gradients. Absorbance spectra are shown as solid lines and fluorescence emission spectra at 77 K are shown as dotted lines. The excitation wavelength for the fluorescence emission spectra was 590 nm. Spectra were normalized at their maximum for ease of comparison.


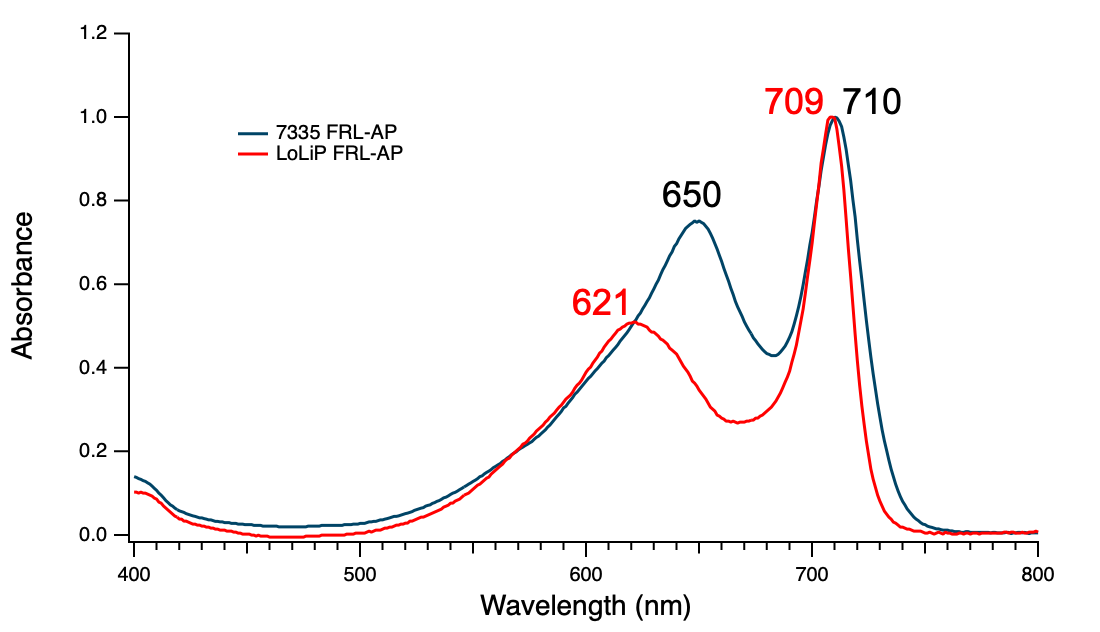


**Figure S2. Comparison of the absorbance spectrum of the helical FRL-AP (ApcD4-ApcB3) from *Thermostichus* sp. (formerly *Synechococcus* sp.) produced during LoLiP (red line) with the AP core complexes from *Synechococcus* 7335 (black line) produced during FaRLiP.** Note the significantly broadened and enhanced absorbance around 650 nm and the increase in absorbance from 710 to 725 nm in the complexes from *Synechococcus* 7335. Spectra were normalized at their maximum for ease of comparison.


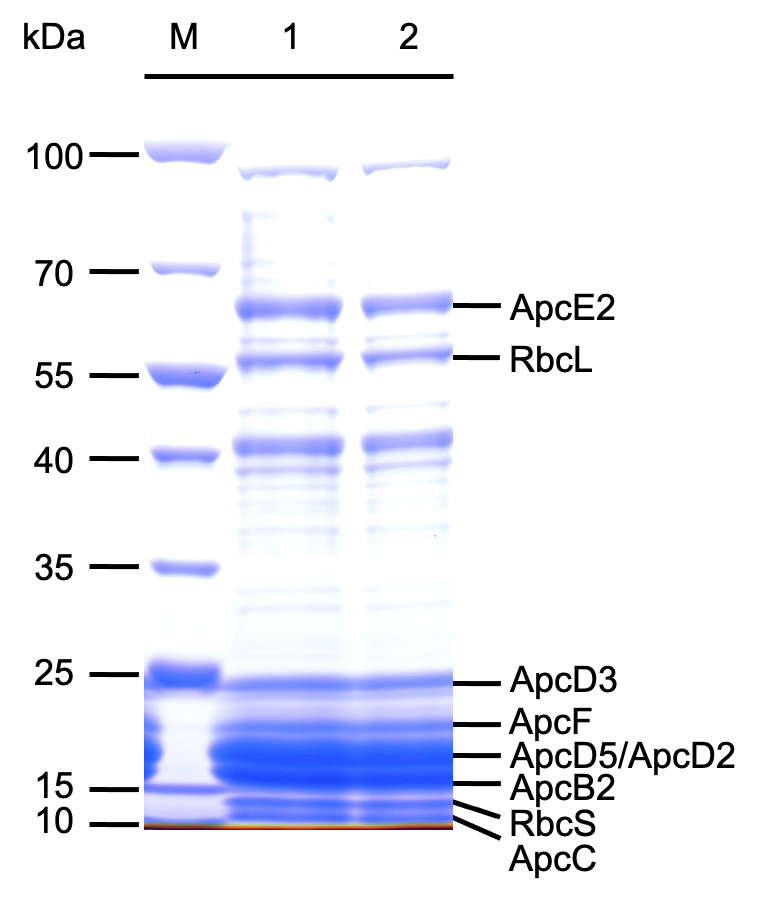


**Figure S3. SDS-PAGE analysis of the FaRLiP-AP core complex isolated from cells of *Synechococcus* 7335 grown in FRL.** Lane M, molecular mass markers; masses are indicated at the left in kDa. Lanes 1 and 2, isolated core complexes after two rounds of sucrose gradient centrifugation. Lane 1, 20 µg; Lane 2, 15 µg. Gel was stained with Coomassie blue. Polypeptides identities are indicated at the right.


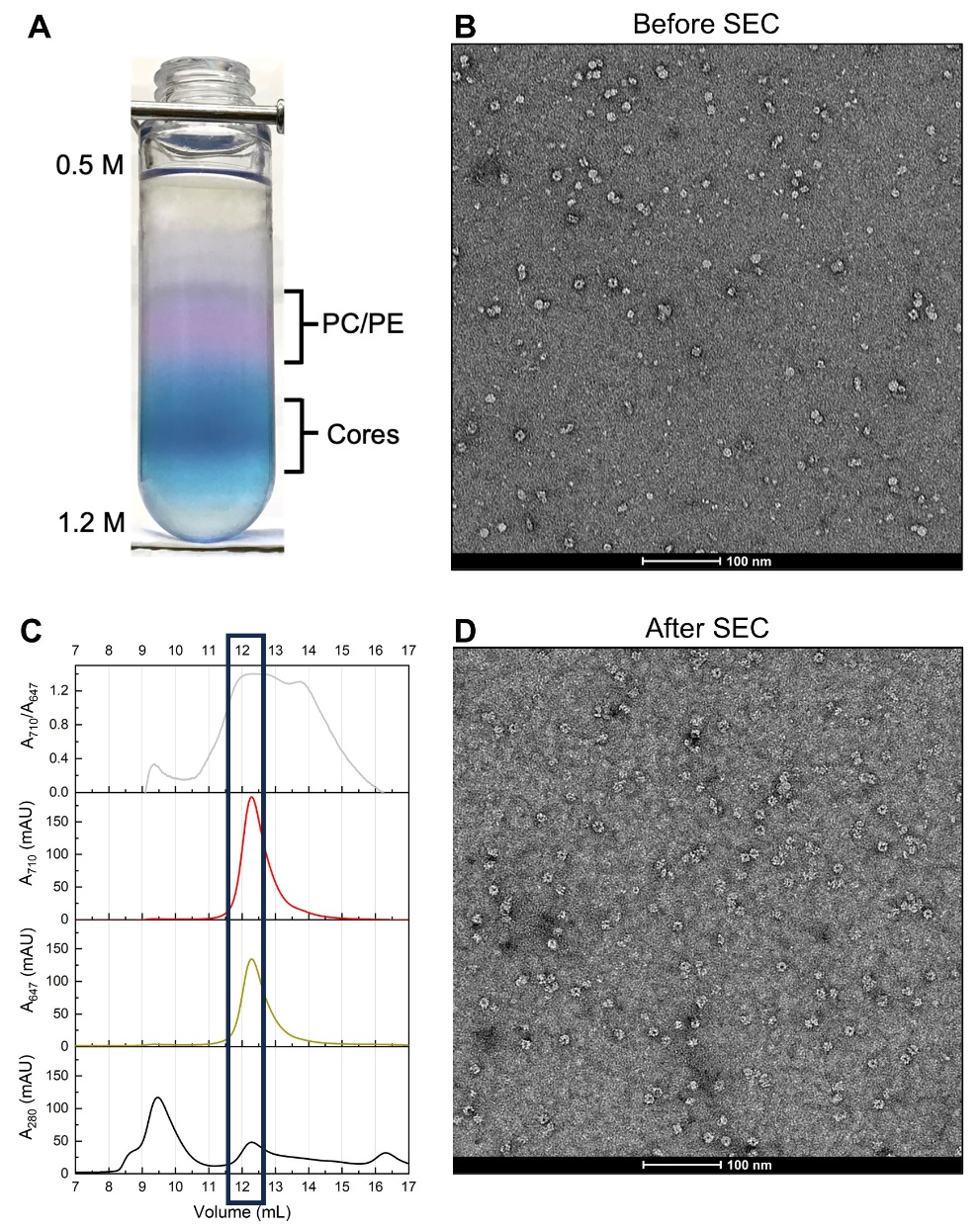


**Figure S4. Preparation of FaRLiP-AP core sample for cryo-EM.** (A) Image of the second sucrose gradient. (B) Transmission electron microscopy micrograph image of negatively stained protein prior to size exclusion chromatography. (C) Size exclusion chromatography (SEC) of buffer-exchanged and concentrated “Cores” band from the second sucrose gradient shown in panel A. The boxed fractions were collected and concentrated for single-particle cryo-EM. (D) Transmission electron microscopy micrograph image of negatively stained protein after size exclusion chromatography


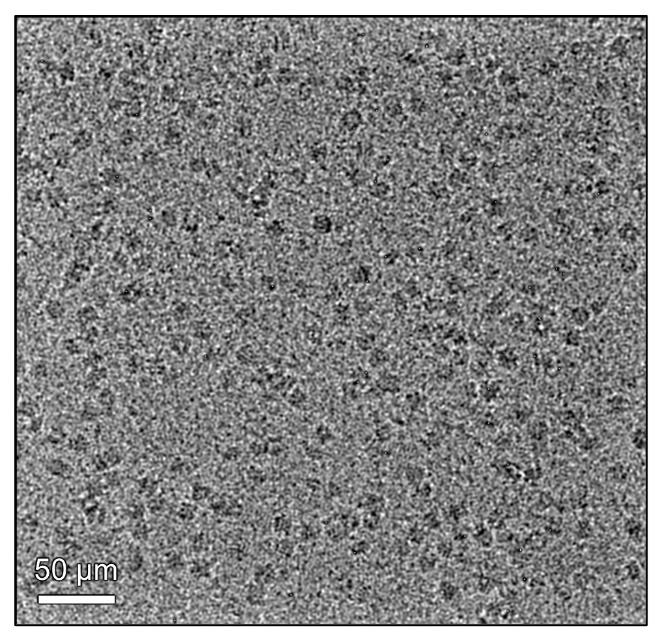


**Figure S5. Screening micrograph image of FaRLiP-AP core complexes after size exclusion chromatography.** The sample was plunge frozen at a concentration of ~1 mg/mL protein and was imaged on a ThermoFisher Glacios cryo-transmission electron microscope.

**
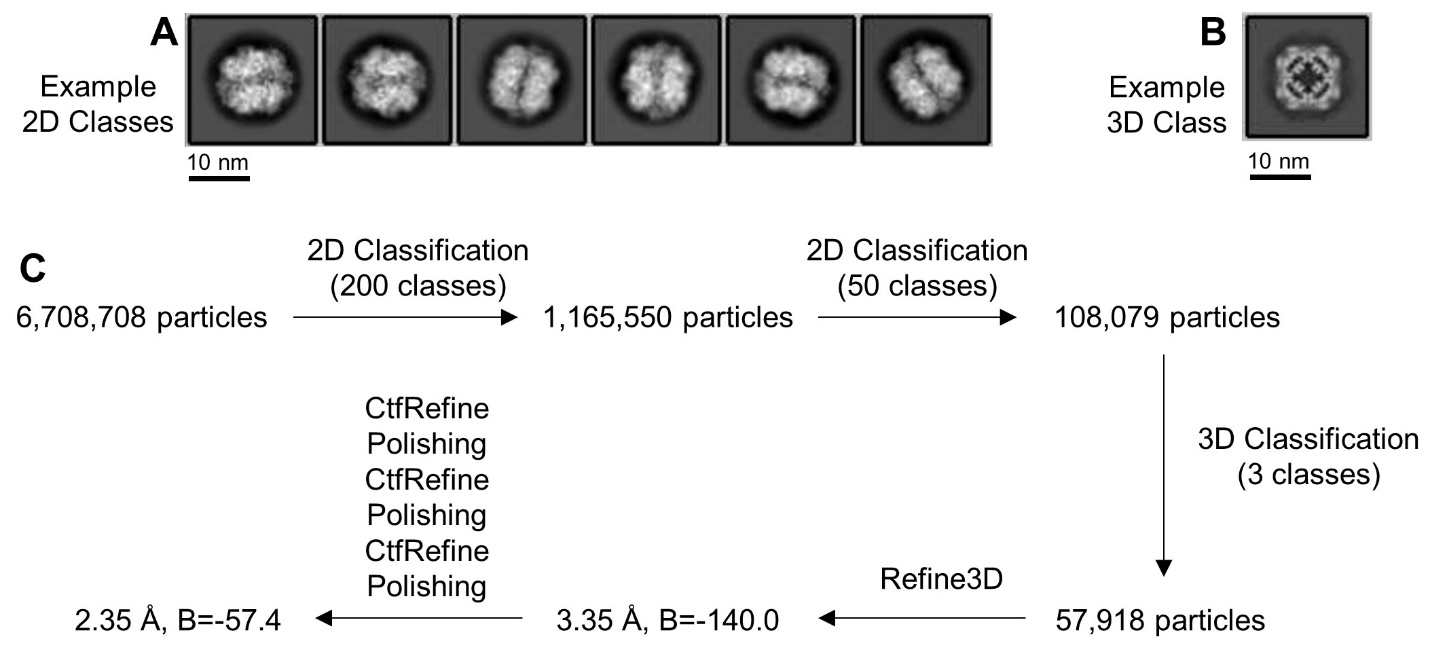
**

**Figure S6. Cryo-EM data processing for Rubisco.** (A) Example 2D classes. (B) Example 3D class. (C) Processing workflow.


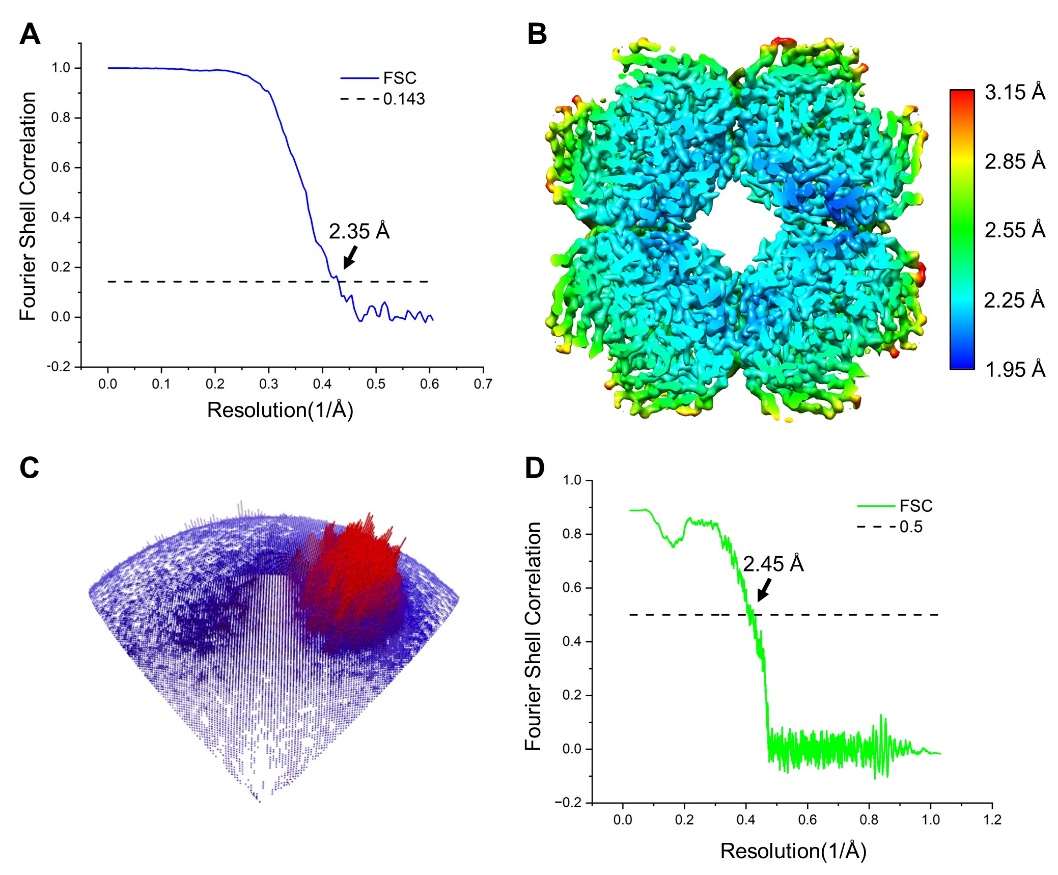


**Figure S7. Cryo-EM data processing for Rubisco.** (A) Map-map Fourier shell correlation. (B) Local resolution map. (C) Angular distribution of particles. (D) Map-model Fourier shell correlation.


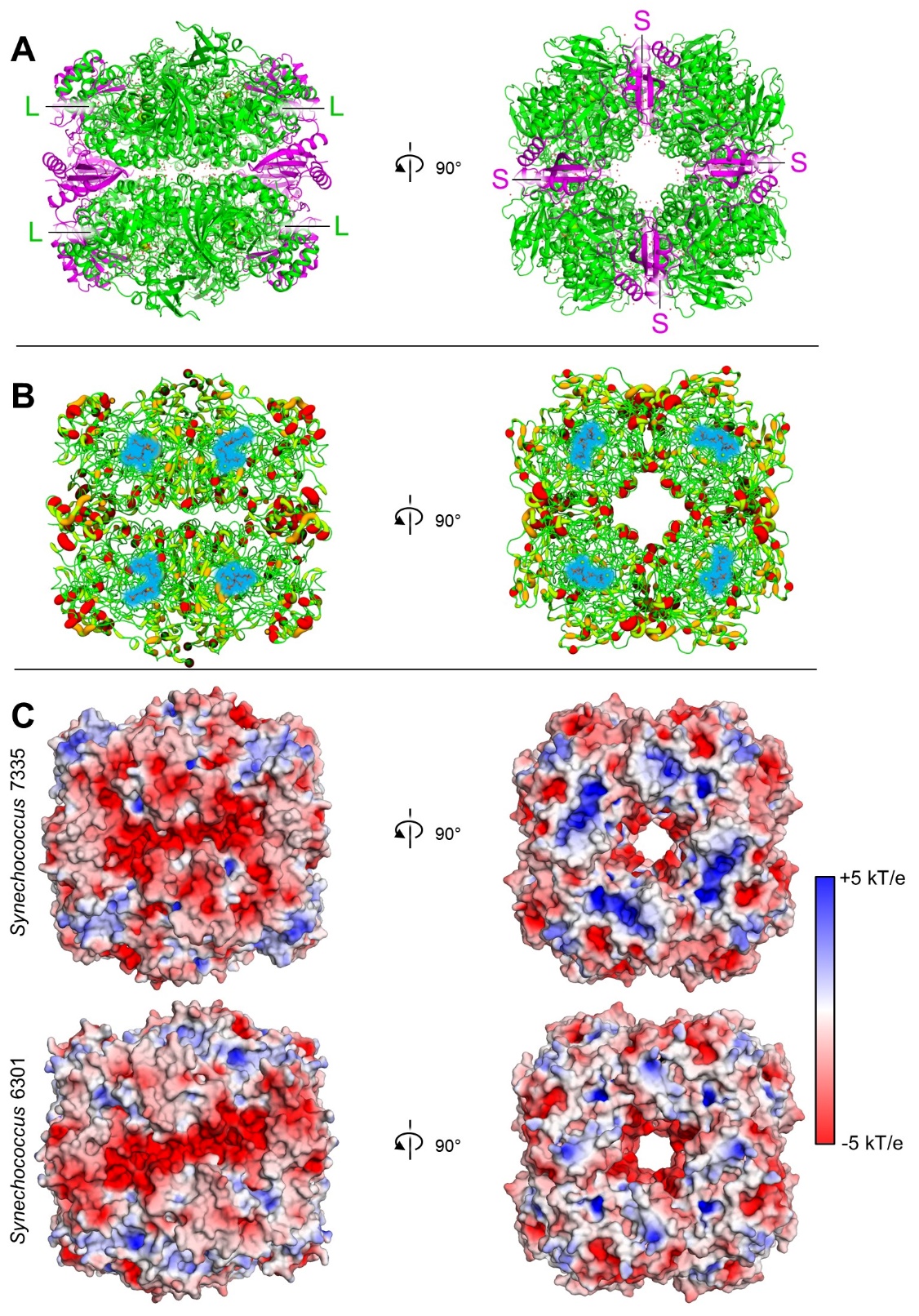


**Figure S8. Structural comparison of Rubisco from *Synechococcus* 7335 and Rubisco from *Synechococcus* 6301.** (A) Structure of Rubisco from *Synechococcus* 7335. The large and small subunits are labeled L (green) and S (magenta), respectively. (B) Structure of Rubisco from *Synechococcus* 7335 colored by sequence conservation comparison to the sequences from *Synechococcus* 6301. Green corresponds to full conservation and red corresponds to no conservation. The RuBP cofactors are shown with a blue glow. (C) Surface electrostatic map comparison of Rubisco from *Synechococcus* 7335 and Rubisco from *Synechococcus* 6301.


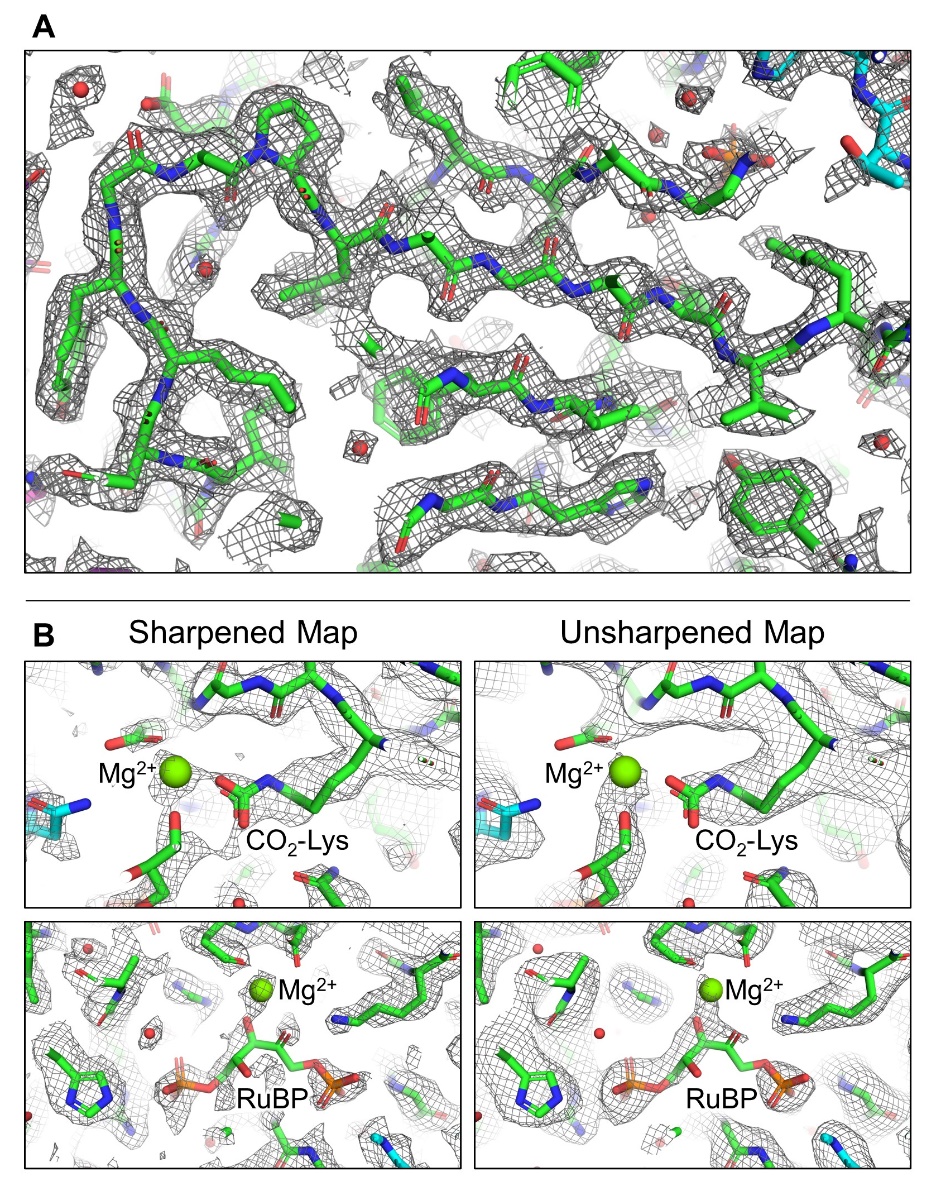


**Figure S9. Cryo-EM structure and map of selected regions from *Synechococcus* 7335 Rubisco determined herein.** (A) Example β-sheet region showing the quality of the map. (B) Views of the carbamylated Lys and RuBP in the active site of Rubisco. Both the sharpened and unsharpened maps are shown.


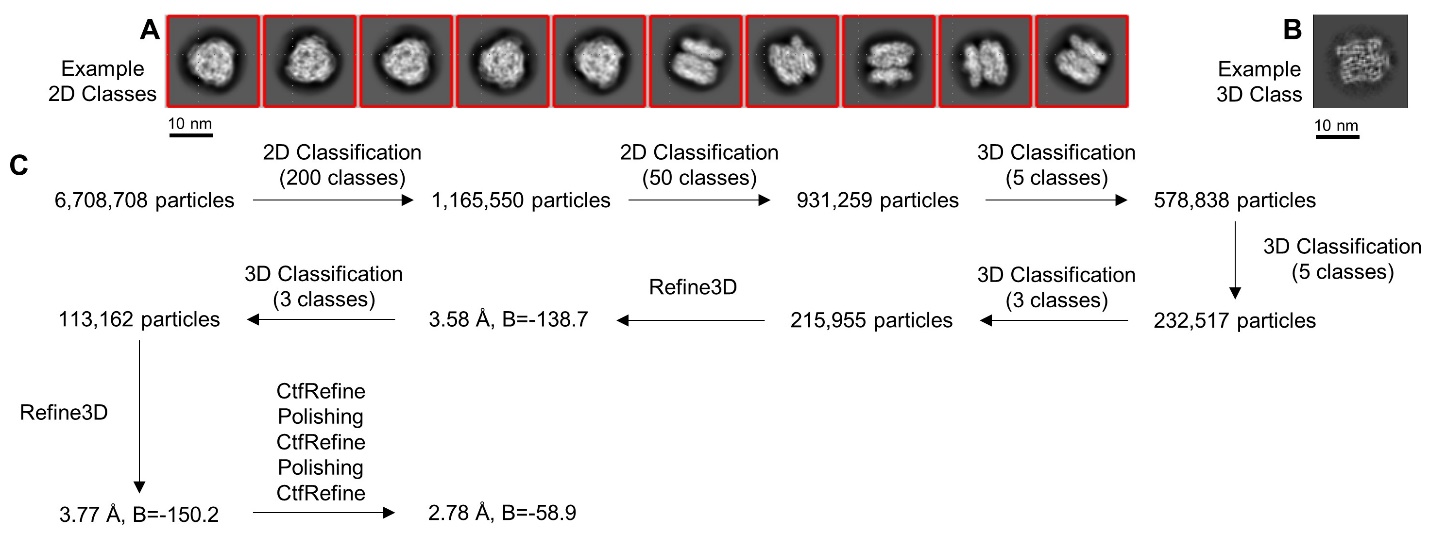


**Figure S10. Cryo-EM data processing for FaRLiP-AP cores.** (A) Example 2D classes. (B) Example 3D class. (C) Processing workflow.


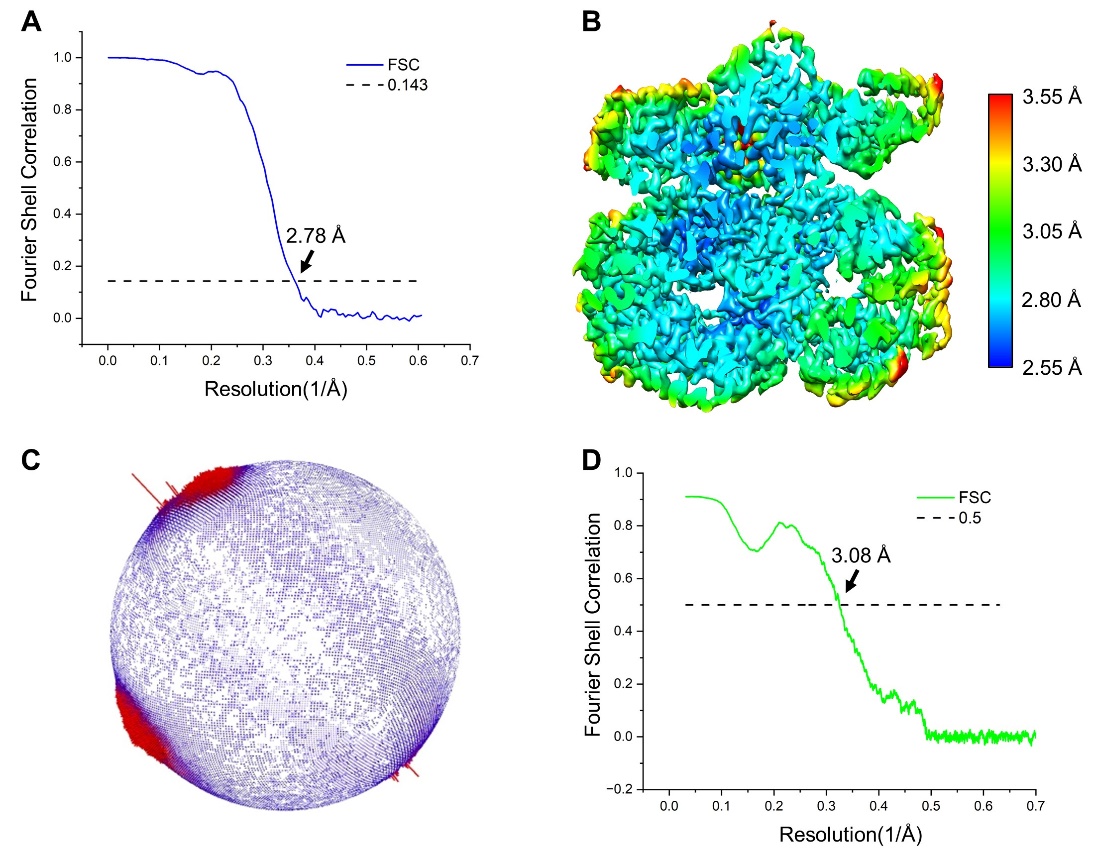


**Figure S11. Cryo-EM data processing for FaRLiP-AP cores.** (A) Map-map Fourier shell correlation. (B) Local resolution map. (C) Angular distribution of particles. (D) Map-model Fourier shell correlation.


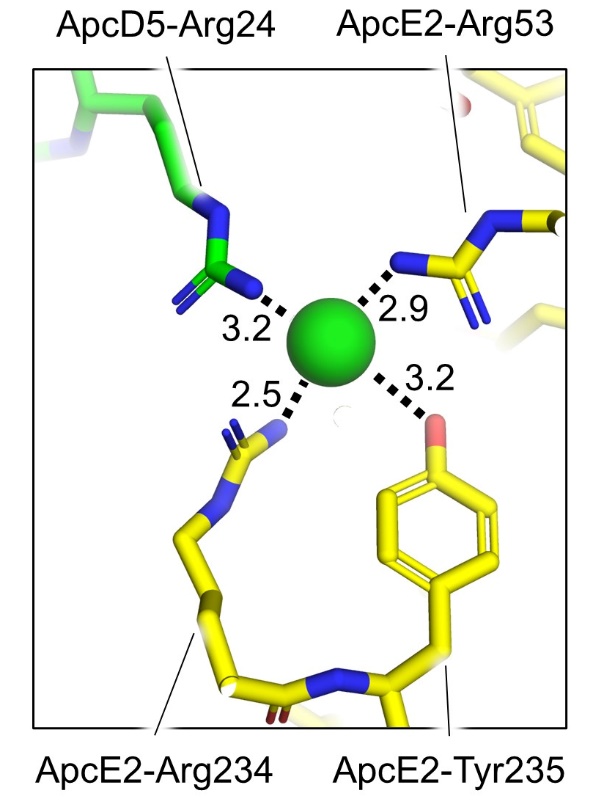


**Figure S12. Anion tentatively modeled as a chloride.** The green sphere shows the position of the modeled Cl^–^ anion. Residues within interaction distance of the anion are shown and labeled.

**
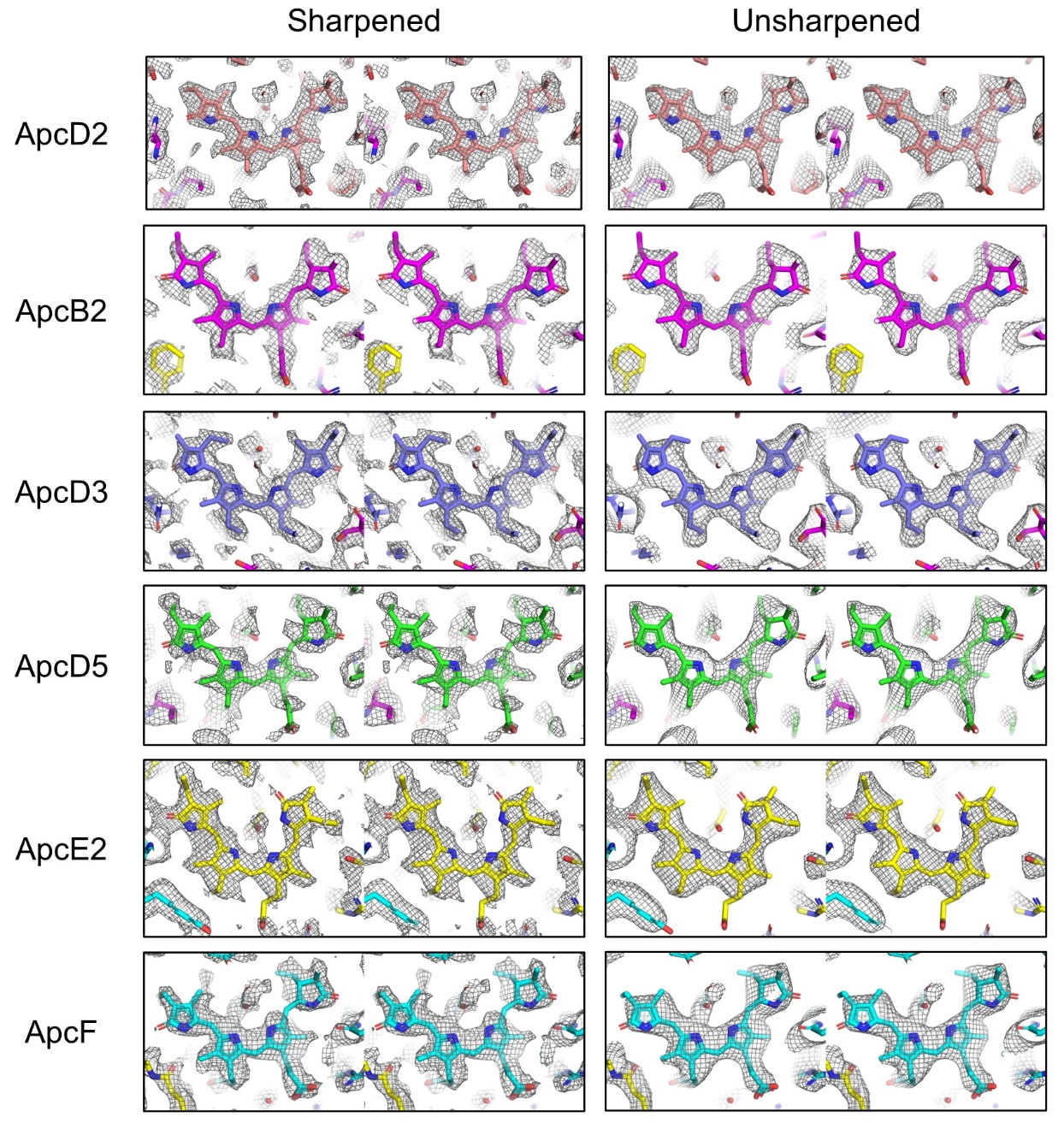
**

**Figure S13. Stereo views of chromophores in sharpened and unsharpened maps.** The left column shows the sharpened map and the right column shows the unsharpened map. For ApcD5 and ApcB2, only the chromophores in chains C and H are shown, respectively.

ApcE2 FaRLiP *Synechococcus* 7335 IFSLKTLAHKP---TKASESLSFFPSSDSRQHSV 542

ApcE1 *Synechocystis* 6803 VFRLNGGLPGAKVGKNTGTSVK------FGESST 539

ApcE1 *Synechococcus* 7002 VFRLNNELPSGKT-----TNVS------FSESAT 530

:* *:

**Figure S14. Partial sequence alignment of the ApcE region that interacts with a fourth trimer and ApcC in PBS structures.** The ApcE1 sequences correspond to those present in PBS structures from *Synechocystis* 6803 (PDB 7SC9) and *Synechococcus* 7002 (PDB 7EXT). These interact with a fourth trimer and ApcC that we suggest are missing in the FaRLiP-AP core structure. The region that could not be modeled in the FaRLiP-AP core structure is highlighted in grey.

**A**

α-subunits (A1, D1, D2, D3, D4, and D5)

ApcA1 Anabaena sp. PCC 7120 (WP_010994626.1) ----------MSLIIKSILNADAEARYFRPGELAQIKGFNASAASRLRLVQALTENRERI 50

ApcA1 Synechococcus sp. PCC 6301 (WP_011243498.1) ----------MSIVSKSIVNADAEARYLSPGELERIKTFVVGGDRRLRIAQTIAESRERI 50

ApcA1 Synechococcus sp. PCC 7335 (WP_006456136.1) ----------MSIVTKSIVNADAEARYLSPGELDRIKGFVTSGERRVRIAQVLTESRERI 50

ApcA1 Synechocystis sp. PCC 6803 (WP_010872503.1) ----------MSIVTKSIVNADAEARYLSPGELDRIKAFVTGGAARLRIAETLTGSRETI 50

ApcA1 Synechococcus sp. PCC 7002 (WP_012307540.1) ----------MSIVTKSIVNADAEARYLSPGELDRIKAFVTSGESRLRIAETLTGSRERI 50

ApcA1 Calothrix sp. PCC 7507 (WP_015126817.1) ----------MSIVTKAIVNADAEARYLSPGELDRIKSFVGSGERRLRIAQVLTDNRERL 50

ApcA1 Thermosynechococcus vestitus BP-1 (WP_011056801.1) MRDVLSGVSNMSVVTKSIVNADAEARYLSPGELDRIKNFVSTGERRLRIAQTLTENRERI 60

ApcA1 Anabaena sp. PCC 7120 (WP_010994198.1) ----------MSIVTKSIVNADAEARYLSPGELDRIKSFVAGGQQRLRIAQALTDNRERL 50

ApcA1 Chlorogloeopsis sp. PCC 9212 (WP_026087462.1) ----------MSIVTKSIVNADAEARYLSPGELDRIKSFVSGGEKRLRIAQVLTDNRERI 50

ApcA1 Chroococcidiopsis thermalis PCC 7203 (WP_015156256.1) ----------MSIVTKSIVNADAEARYLSPGELDRIKSFVTSGERRLRIAQALTDNRERI 50

ApcA1 Fischerella thermalis PCC 7521 (WP_009457007.1) ----------MSIVTKAIVNADAEARYLSPGELDRIKSFVTSGERRLRIAQVLTDNRERI 50

ApcA1 Leptolyngbya sp. JSC-1 (WP_036003991.1) ----------MSIVTKSIVNADAEARYLSPGELDRIKGFVTSGERRLRIAQVLTDSRERI 50

ApcD1 Synechococcus sp. A1463 (WP_011431562.1) ----------MSVINQIIETADDQLRYLSVSELQAIKDYMSSGEKRLQIAQVLTENKKRI 50

ApcD1 Synechocystis sp. PCC 6803 (WP_010871516.1) ----------MSVVSQVILQADDQLRYPTSGELKGIQAFLTTGAQRIRIAETLAENEKKI 50

ApcD1 Synechococcus sp. PCC 7002 (WP_012307741.1) ----------MSVVSQVILRADDELRYPSSGELSGIKNFLATGAVRIRIAEALADNEKKI 50

ApcD1 Leptolyngbya sp. PCC 7376 (WP_015135290.1) ----------MSVVSQVILKADDELRYPSSGELTGMESFLATGAIRIRIAEALADSEKKI 50

ApcD1 Synechococcus sp. PCC 6301 (WP_011243585.1) ----------MTIVSQVILKADDELRYPSGGELKNITDFFKTGEQRLRIAQVLSDSEKKI 50

ApcD1 Thermosynechococcus sp. CL-1 (QEQ1666.1) MRDVLSGVSNMSVISQVLLKADDELRYPTTGELQTISDFFQTGEQRLRIATTLAENEKRI 60

ApcD1 Arthrospira platensis (GCE93786.1) ----------MTVVSQVILKADDELRYPTTGELKNISDFLKTGEQRLRIVDTLTENEKKI 50

ApcD1 Synechococcus sp. PCC 7335 (WP_006454063.1) ----------MSVVSQVILNADDELRYPTSGELKGIENFLKTGDQRMRIAQILADNEKKI 50

ApcD1 Leptolyngbya sp. JSC-1 (WP_036003919.1) ----------MSVVTQVILNADEELRYPSSGELKSIQNFLQTGSQRMRIAATLADNEKKI 50

ApcD1 Chroococcidiopsis thermalis PCC 7203 (WP_015152396.1) ----------MSVVSQVILKADDELRYPSAGELENIKNFLQTGIQRMRIAATLAENEKKI 50

ApcD1 Nostoc sp. PCC 7524 (WP_015139163.1) ----------MTVISQVILQADDELRYPSSGELKSIREFLQTGLQRTRIAGTLAENEKKI 50

ApcD1 Anabaena sp. PCC 7120 (WP_010997797.1) ----------MTVISQVILQADDELRYPSSGELKSISDFLQTGVQRTRIVATLAENEKKI 50

ApcD1 Calothrix sp. PCC 7507 (WP_015130965.1) ----------MTVISQVIFKADDELRYPSSGELKSIQNFLQTGIQRTRIAATLAENEKKI 50

ApcD1 Chlorogloeopsis fritschii PCC 9212 (WP_016878526.1) ----------MTVISQVILKADDELRYPSSGELKSIKDFLQTGEQRVRIANTLAENEKKI 50

ApcD1 Nostoc sp. PCC 7107 (WP_015113750.1) ----------MTVISQVILKADDELRYPSSGELKSIKEFLQTGEQRTRIAATLAENEKKI 50

ApcD2 Synechococcus sp. PCC 7335 (WP_006455320.1) ----------MSVISQVIATADREVRYLSKGELDAINRFFNNGPQRLRIVSILNSNAEEI 50

ApcD2 Leptolyngbya sp. JSC-1 (IMG: 2022833634) ----------MSIITNVIATADREARYLNVEELNAVQNFYAAGRDRLRLAATLAANEQRI 50

ApcD2 Chroococcidiopsis thermalis PCC 7203 (WP_015153115.1) ----------MSIITKAIASADREARYLSPGELRTIRDFYNGGENRLRIATTLIENRKEI 50

ApcD2 Calothrix sp. PCC 7507 (WP_015126588.1 ----------MSIITKSIASADREARYLSPGELNAIRDFYEGGFYRMRIAITLTENEQKI 50

ApcD2 Chlorogloeopsis sp. PCC 9212 (WP_016873422.1) ----------MSIIIKSIVNADREARYLNAGELRAIQEFYENGVSRLNFAVTLTENEKTI 50

ApcD2 Fischerella thermalis PCC 7521 (WP_009453700.1) ----------MSIIIKSIVNADREARYLNAGELSAIQEFYESGVSRLNLAMTLTENEQKI 50

ApcD3 Synechococcus sp. PCC 7335 (WP_006456515.1) ----------MSIVKQIISNADEELRYPTPGELEMIRSFCKTGASQIQLAKTLESHAPTI 50

ApcD3 Leptolyngbya sp. JSC-1 (MBF2050150.1) ----------MSLVKQVIQNADEELRYPTPGEIRMIQNFCQTGERRIQIAQKLAAAEQDL 50

ApcD3 Calothrix sp. PCC 7507 (WP_015126586.1) ----------MSLVKQVILNADEELRYPTPAEIRMIQNFCHSGEKRIRIATTLAKNQNRL 50

ApcD3 Chlorogloeopsis sp. PCC 9212 (WP_016873424.1) ----------MSLVKQVIENADEQMRYPTPGEIRMIQKFCHSGDKRIRIATVLASNQNRL 50

ApcD3 Fischerella thermalis PCC 7521 (WP_009453698.1) ----------MSLVKQVIENADEQMRYPSPGEIRMIQNFCHSGDKRIRIATTLASNQNRL 50

ApcD3 Chroococcidiopsis thermalis PCC 7203 (WP_015153117.1) ----------MSIVKQMILNADEEVRYLTPGEIHALQNFYRSGTERIRLAKVLAQNEKKI 50

ApcD4 Synechococcus sp. A1463 (WP_099812040.1) ----------MSIVAQVIAQSDAADRFLSSAEIAKLEDFFSKGQVRIRAAQKLAENEQKI 50

ApcD4 Chroococcidiopsis sp. FACHB-1243 (WP_192159074.1) ----------MSLVAQVIAQSDEADRFLSRTELDKLQDFFKTGETRLKVAQILSQNEQKI 50

ApcD4 Chroococcidiopsis thermalis PCC 7203 (WP_015156293.1) ----------MSLVAQVIAQSDEADRFLSRTELDKLQDFFKTGETRLKVAQILSKNEQKI 50

ApcD4 Gloeocapsa sp. PCC 7428 (WP_015190166.1) ----------MSIVAQVIAQSDDANRFLSNTELDKLQDFFRTGEQRLKVAQILTQNEQKI 50

ApcD4 Chlorogloeopsis sp. PCC 9212 (WP_016874155.1) ----------MSIVAQVIAQSDDAARFLSRTELDKLDNFFKSGETRLRVAQILAQNEQNI 50

ApcD4 Halomicronema hongdechloris (WP_080809693.1) ----------MSIVAKVIAQSDEANRFLSSAELNKLQDFFKDGAVRISAAQKLAANQQKL 50

ApcD4 Leptolyngbya sp. PCC 6406 (WP_008312402.1) ----------MSIVAKVIAQSDEASRFLSSAELTKLQDFFGNGTVRISASQKLASNQQKI 50

ApcD4 Xenococcus sp. PCC 7305 (WP_006512043.1) ----------MSIVAKVIAQSDRSDRFPSTAEITQLQDFFNNSSVRISTAQKLSENQQKI 50

ApcD4 Pleurocapsa sp. CCALA 161 (WP_106232622.1) ----------MSIVAKVIAQSDMSDRFLSSAELTQLQDFFNKGGVRISAAQKLAANEQKI 50

ApcD4 Synechococcus sp. PCC 7335 (WP_006454943.1) ----------MSIVAKAIAQSDRSDRFLGSTELTQLQDFFNNSSARISAAQKLAANQQKI 50

ApcD4 Romeria gracilis (WP_193906454.1) ----------MSIVAKVIAQSDRADRFLSSAELTQLQDFFNDSGARISAAQKLAANQQKI 50

ApcD4 Gloeomargarita lithophora (WP_071455395.1) ----------MSLVAQVIAQSDAAERFLSSTELNKLAEFFSKGALRVRVAEKLAANEKKI 50

ApcD5 Synechococcus sp. PCC 7335 (WP_006456289.1) ----------MSLVTELILSADSEARYPAPKELRIFQDFVKTGEQRVRIAKALAANEERI 50

ApcD5 Chroococcidiopsis thermalis PCC 7203 (WP_015153113.1) ----------MSIVTELILNADSESRYPAPKEIQVYQNFVKTGEQRIRIAKILAENEQRI 50

ApcD5 Leptolyngbya sp. JSC-1 (IMG: 2022833632) ----------MSVVTELILNADSESRYPVPKEMRIFQDFLRSGEQRIRIAATLAENEQRI 50

ApcD5 Calothrix sp. PCC 7507 (WP_015126590.1) ----------MSIVTELILNADSESRYPAPKELRVFQEFLKSGDQRIRIAKILSQNEQQI 50

ApcD5 Chlorogloeopsis sp. PCC 9212 (WP_016873420.1) ----------MSVVTELILNADSESRYPAPKELRIFQDFVKTGDQRIRIAKILSDNEQLI 50

ApcD5 Fischerella thermalis PCC 7521 (WP_009453702.1) ----------MSVVTELILNADSESRYPAPKELRIFQDFLRTGDQRIRIAKILAENEQLI 50

*::: : : :* *: *: . : : :

|||||||||||||||||

ApcA1 Anabaena sp. PCC 7120 (WP_010994626.1) VKQSANQLFQKRPDIVSPGGNAYGQDMTATCLRDMDYYLRLITYSIVAGDSTPIQEIGVI 110

ApcA1 Synechococcus sp. PCC 6301 (WP_011243498.1) VKQAGNQLFQKRPDVVSPGGNAYGEDMTATCLRDLDYYLRLVTYGVVSGDITPIEEIGIV 110

ApcA1 Synechococcus sp. PCC 7335 (WP_006456136.1) VKTAGDQLFQKRPDVVSPGGNAYGEEMTATCLRDMDYYLRLITYGVVAGDVAPIEEIGLV 110

ApcA1 Synechocystis sp. PCC 6803 (WP_010872503.1) VKQAGDRLFQKRPDIVSPGGNAYGEEMTATCLRDMDYYLRLVTYGVVSGDVTPIEEIGLV 110

ApcA1 Synechococcus sp. PCC 7002 (WP_012307540.1) IKSAGDALFQKRPDVVSPGGNAYGEEMTATCLRDMDYYLRLITYGVVAGDVTPIEEIGLV 110

ApcA1 Calothrix sp. PCC 7507 (WP_015126817.1) VKQAGEQLFQKRPDVVSPGGNAYGQELTATCLRDLDYYLRLVTYGIVAGDVTPIEEIGVI 110

ApcA1 Thermosynechococcus vestitus BP-1 (WP_011056801.1) VKQAGDQLFQKRPDVVSPGGNAYGEEMTATCLRDLDYYLRLVTYGIVAGDVTPIEEIGLV 110

ApcA1 Anabaena sp. PCC 7120 (WP_010994198.1) VKQAGDQLFQKRPDVVSPGGNAYGQEMTATCLRDLDYYLRLVTYGIVAGDVTPIEEIGVI 110

ApcA1 Chlorogloeopsis sp. PCC 9212 (WP_026087462.1) VKQAGDQLFQKRPDVVSPGGNAYGQEMTATCLRDLDYYLRLVTYGVVAGDVTPIEEIGIV 110

ApcA1 Chroococcidiopsis thermalis PCC 7203 (WP_015156256.1) VKQAGDQLFQKRPDVVSPGGNAYGQEMTATCLRDLDYYLRLITYGVVAGDVTPIEEIGIV 110

ApcA1 Fischerella thermalis PCC 7521 (WP_009457007.1) VKQAGDQLFQKRPDVVSPGGNAYGQEMTATCLRDLDYYLRLITYGVVSGDVTPIEEIGVV 110

ApcA1 Leptolyngbya sp. JSC-1 (WP_036003991.1) VKQAGDQLFQKRPDVVSPGGNAYGEEMTATCLRDLDYYLRLITYGVVSGDVTPIEEIGIV 110

ApcD1 Synechococcus sp. A1463 (WP_011431562.1) IDQAQKQLFAKRPEYIQPGGNAYGEKRYNQCLRDYDWYLRLVTYGIIAGSKEPIESIGLI 110

ApcD1 Synechocystis sp. PCC 6803 (WP_010871516.1) VDQAQKQLFKKHPEYRAPGGNAYGQRQYNQCLRDYGWYLRLVTYGVLAGNKEPIETTGLI 110

ApcD1 Synechococcus sp. PCC 7002 (WP_012307741.1) VDQAQKQLFSIHPEYRTSGGNAATTKQYNQCLRDYGWYLRLVTYGILAGDKDPIERIGLI 110

ApcD1 Leptolyngbya sp. PCC 7376 (WP_015135290.1) VDEAQKKLFAIHPEYRAAGGNASTTKRYNQCLRDYGWYLRLVTYGVLAGDKDPIENIGLI 110

ApcD1 Synechococcus sp. PCC 6301 (WP_011243585.1) VDQASRKLWQRRPDFIAPGGNAYGQRQRAQCLRDYGWYLRLITYGVLAGDKEPIESIGLL 110

ApcD1 Thermosynechococcus sp. CL-1 (QEQ1666.1) VEQASKQLWQKRPDFISPGGNAYGQKQRALCLRDYGWYMRLITYGILAGDKDPIERTGII 130

ApcD1 Arthrospira platensis (GCE93786.1) VDRASAQLWKKRPDFIAPGGNAAGQRERSLCLRDYGWYLRVITYGILSGDKDPIESIGLI 110

ApcD1 Synechococcus sp. PCC 7335 (WP_006454063.1) VEQASGALWKLRPDFIAKGGNAYGQKQRALCLRDYGWYLRLITYSVLAGDKEPIESIGLI 110

ApcD1 Leptolyngbya sp. JSC-1 (WP_036003919.1) VQEASKELWKKRPDFIAPGGNAYGDKQRALCLRDYGWYLRLISYGVLAGDKEPIEKIGLI 110

ApcD1 Chroococcidiopsis thermalis PCC 7203 (WP_015152396.1) VQEASKKLWQKRPDFIAPGGNAYGERQRALCLRDYGWYLRLITYGVLAGDKEPIEKIGLV 110

ApcD1 Nostoc sp. PCC 7524 (WP_015139163.1) VQEATKQLWQKRPDFIAPGGNAYGEKQRALCIRDFGWYLRLITYGVLAGDIEPIEKIGII 110

ApcD1 Anabaena sp. PCC 7120 (WP_010997797.1) VQEATKQLWQKRPDFIAPGGNAYGERQRALCIRDFGWYLRLITYGVLAGDIEPIEKIGII 110

ApcD1 Calothrix sp. PCC 7507 (WP_015130965.1) VQEATKQLWQKRPDFISPGGNAYGERQRSLCIRDFGWYLRLITYGVLAGDKEPIEKIGLI 110

ApcD1 Chlorogloeopsis fritschii PCC 9212 (WP_016878526.1) VQEATKQLWQKRPDFIAPGGNAYGERQRALCIRDFGWYLRLITYGVLAGDKEPIEKIGLV 110

ApcD1 Nostoc sp. PCC 7107 (WP_015113750.1) VQEATKQLWQKRPDFIAPGGNAYGERQRALCIRDFGWYLRLITYGVLAGDKEPIEKIGLI 110

ApcD2 Synechococcus sp. PCC 7335 (WP_006455320.1) VEKGARRFWQRCPITPSNSDNQ---QFQASCLRDQAWFIRLISYAVAVGDVDPLEASGVR 107

ApcD2 Leptolyngbya sp. JSC-1 (IMG: 2022833634) VEQATQRFWQRCPVTPSNSGNP---TFQSSCMRDQSWYVRLVTYAIVLGDIEPIETSGVR 107

ApcD2 Chroococcidiopsis thermalis PCC 7203 (WP_015153115.1) VERGSLKFWECCPDTPSNSGNR---TYRASCLRDQDWYIRLIAYTVIVGDVEPLKDIGIV 107

ApcD2 Calothrix sp. PCC 7507 (WP_015126588.1 VEKASLKFWERCHDTPSNSGNR---IYRSSCLRDQSWYIRLITYSIVLQDIEPLAEIGTI 107

ApcD2 Chlorogloeopsis sp. PCC 9212 (WP_016873422.1) VEKASLKFWERCPNTPSNSGNR---MYRNSCLRDQSWYIRLITYALVVGDVEPLAEIGTI 107

ApcD2 Fischerella thermalis PCC 7521 (WP_009453700.1) VEKASLKFWERCPNTPSNSGNR---MYRNSCLRDQSWYIRLITYAVVVGDVEPLAAIGTI 107

ApcD3 Synechococcus sp. PCC 7335 (WP_006456515.1) VERGTRKFWQICPRTPSNSGSP---RKTEAAQRDMSWYIRLISYCLLAGNDQPLREIGLL 107

ApcD3 Leptolyngbya sp. JSC-1 (MBF2050150.1) VQKGSQRFWKRCPVTPSNSGNP---RKTASCQRDQGWYIRLVAYCVLAGSEQPLADIGTV 107

ApcD3 Calothrix sp. PCC 7507 (WP_015126586.1) VEKASDKFWKVCPVTPSNSGNM---RKTASCQRDQGWYIRLVAYCILAGNEQPLEDIGTI 107

ApcD3 Chlorogloeopsis sp. PCC 9212 (WP_016873424.1) VEKASQKFWKRCPITPSNSGNM---RKTASCQRDQGWYIRLVAYCVLAGSEQPLTEIGTV 107

ApcD3 Fischerella thermalis PCC 7521 (WP_009453698.1) VERASQKFWKRCPVTPSNSGNM---RKTASCQRDQGWYIRLVAYCVLAGNEQPLTEIGTV 107

ApcD3 Chroococcidiopsis thermalis PCC 7203 (WP_015153117.1) VERATQKFWKICPRTPSNSGNA---RKTEAAMRDIGWYIRLVSYCLLAGNEKPLEEIGLI 107

ApcD4 Synechococcus sp. A1463 (WP_099812040.1) VQEGSKRFWAKCPNTPSNKGNP---QKTALCQRDQGWYIRLVSYCILAGNDKPLEDIGLN 107

ApcD4 Chroococcidiopsis sp. FACHB-1243 (WP_192159074.1) VQEGSQRFWKVIPNTPSNSGDP---KKTALCQRDQAWYLRLITYAVLAGNMKPLDDIGIN 107

ApcD4 Chroococcidiopsis thermalis PCC 7203 (WP_015156293.1) VQEGSQRFWKVIPNTPSNSGDP---KKTALCQRDQAWYLRLITYAVLAGNMKPLDDIGIN 107

ApcD4 Gloeocapsa sp. PCC 7428 (WP_015190166.1) VQEGSRRFWQVVPNTPSNSGDP---QKTALCQRDQSWYLRLISYAVLAGNMKPLEDIGVD 107

ApcD4 Chlorogloeopsis sp. PCC 9212 (WP_016874155.1) VEEGSRRFWKIVPNTPSNSGDP---KKTALCQRDQSWYLRLITYAVLAGNTKPLEDIGVD 107

ApcD4 Halomicronema hongdechloris (WP_080809693.1) VDEGSKRYWAQCPDAPSNSGDP---QKTANCQRDQGWYIRIISYCVLAGNGKPMEDLGIE 107

ApcD4 Leptolyngbya sp. PCC 6406 (WP_008312402.1) VDEGSKRFWAQCPNTPSNSGDS---QKTMLCQRDQGWYIRLVSYCVLAGNAKPLEDIGLD 107

ApcD4 Xenococcus sp. PCC 7305 (WP_006512043.1) VEEGSKQFWAQCSNTPSNKGDA---TKTALCQRDQGWYIRLVSYCVLAGNSKPLEDIGLD 107

ApcD4 Pleurocapsa sp. CCALA 161 (WP_106232622.1) VDQGSKQFWSQCSNTPSNSGDS---QKTALCQRDQGWYIRLVSYCVLAGNSKPLEDIGLD 107

ApcD4 Synechococcus sp. PCC 7335 (WP_006454943.1) VDEGSKQFWNQCPNTPSNSGDK---QKTALCQRDQGWYIRLVSYCILAGNSKPLEDIGLD 107

ApcD4 Romeria gracilis (WP_193906454.1) VEEGSKRFWEQCPNTPSNSGDK---QKTALCQRDQGWYIRLVSYCLLAGNSKPLEDIGLD 107

ApcD4 Gloeomargarita lithophora (WP_071455395.1) VEEGSKRFWAKCSNTPSNRGNA---QKTALCQRDQGWYIRLVSYCVLAGNDKPLDDIGLN 107

ApcD5 Synechococcus sp. PCC 7335 (WP_006456289.1) VQNGSQKFWERCPNTPSNSGVD---RKTASCQRDQGWYVRLIAYSILAGSERPLEDIGTV 107

ApcD5 Chroococcidiopsis thermalis PCC 7203 (WP_015153113.1) VQNGSARFWERVPNTPSNSGNE---RKTASCQRDQGWYIRLIAYSVLAGSEKPLEEIGTI 107

ApcD5 Leptolyngbya sp. JSC-1 (IMG: 2022833632) VQNASAKFWERCPVTPSNSGNP---RKTASCQRDQGWYIRLVAYSVLAGSEKPLSEIGTV 107

ApcD5 Calothrix sp. PCC 7507 (WP_015126590.1) VQNGSLKFWERCPNTPSNSGNE---RKTASCQRDQGWYVRLVAYSILAGSEKPLEEIGTI 107

ApcD5 Chlorogloeopsis sp. PCC 9212 (WP_016873420.1) VQRGSQRFWERCPNTPSNSGNE---RKTASCQRDQGWYVRLVAYSVLAGSEKPLEEIGTV 107

ApcD5 Fischerella thermalis PCC 7521 (WP_009453702.1) VQRGSQKFWERCPNTPSNSGNE---RKTASCQRDQGWYVRLVAYSVLAGSEKPLEEIGTV 107

:. . : . . ** :::*:::* : . *: *

ApcA1 Anabaena sp. PCC 7120 (WP_010994626.1) GVREMYRSLGT-PIEAVAESIRAMKYVATSMMSVEDRAEVDTYFDYLIGAMQ-------- 161

ApcA1 Synechococcus sp. PCC 6301 (WP_011243498.1) GVREMYKSLGT-PIEAVAEGVRELKSAATALLTGEDADEAGAYFDYVIGALS-------- 161

ApcA1 Synechococcus sp. PCC 7335 (WP_006456136.1) GAREMYNSLGT-SIPAMADSIRCMKSVAGSMMSGDDALEAASYFDYVIGGLQ-------- 161

ApcA1 Synechocystis sp. PCC 6803 (WP_010872503.1) GVREMYRSLGT-PIEAVAQSVREMKEVASGLMSSDDAAEASAYFDFVIGKMS-------- 161

ApcA1 Synechococcus sp. PCC 7002 (WP_012307540.1) GVREMYKSLGT-PVDAVAQAVREMKAVATGMMSGDDAAEAGAYFDYVIGAME-------- 161

ApcA1 Calothrix sp. PCC 7507 (WP_015126817.1) GARELYKSLGT-PIEGVAEGIRGLKNVAASLLSAEDASEAGSYFDYLVGALL-------- 161

ApcA1 Thermosynechococcus vestitus BP-1 (WP_011056801.1) GVREMYNSLGT-PIPAVAEGIRAMKNVACSLLSAEDAAEAGSYFDFVIGAMQ-------- 161

ApcA1 Anabaena sp. PCC 7120 (WP_010994198.1) GVREMYKSLGT-PIEAVGEGVRALKNAASTLLSAEDAAEAGSYFDYVVGALQ-------- 161

ApcA1 Chlorogloeopsis sp. PCC 9212 (WP_026087462.1) GVREMYKSLGT-PIDAVAAGVSAMKNVAASLLSADDASEAGAYFDYVAGALA-------- 161

ApcA1 Chroococcidiopsis thermalis PCC 7203 (WP_015156256.1) GVREMYKSLGT-PIEAVAEGVRAMKNVATSMMSGEDAGEAGSYFDYLVGAMQ-------- 161

ApcA1 Fischerella thermalis PCC 7521 (WP_009457007.1) GVREMYKSLGT-PIDAVAAGVNAMKNVAASLLSGEDAAEAGAYFDYLVGAMS-------- 161

ApcA1 Leptolyngbya sp. JSC-1 (WP_036003991.1) GVREMYKSLGT-PIDAVAEGVRAMKSVATSLLSGDDAAEAGTYFDYVIGAMQ-------- 161

ApcD1 Synechococcus sp. A1463 (WP_011431562.1) GVREMYNALNV-PIAGMIDAIVFLKEAALSLLDPDSAAEAAPYFDYIINAMS-------- 161

ApcD1 Synechocystis sp. PCC 6803 (WP_010871516.1) GVKEMYNSLNV-PVPGMVDAVTVLKDAALGLLSAEDANETAPYFDYIIQFMS-------- 161

ApcD1 Synechococcus sp. PCC 7002 (WP_012307741.1) GVKEMYNALGV-PVPGMVDAIRCLKDAALGVLDSEEARIAAPYFDFITQAMS-------- 161

ApcD1 Leptolyngbya sp. PCC 7376 (WP_015135290.1) GVKEMYNALDV-PVTGMIDAIRCLKEAALGVLDLEEASIAAPYFDFITQSMS-------- 161

ApcD1 Synechococcus sp. PCC 6301 (WP_011243585.1) GAREMYNSLGV-PLPGMAEAIRTLKEASLALLSSADATVAAPYFDFLIQGMETI------ 163

ApcD1 Thermosynechococcus sp. CL-1 (QEQ1666.1) GVREMYNSLGV-PMTGMAEAMRCLKDASLALLSTEDAEVAAPYFDYIIQEMS-------- 171

ApcD1 Arthrospira platensis (GCE93786.1) GVKEMYNSLGV-PMPGMVEAIRCLKEASLALLDDEDAKEAAPYFDFIIQAMS-------- 161

ApcD1 Synechococcus sp. PCC 7335 (WP_006454063.1) GVREMYNALDV-PVPGMVEAIRCLKNASLSLMNEEDAAEATPYFDYIIQAMSA------- 162

ApcD1 Leptolyngbya sp. JSC-1 (WP_036003919.1) GVKEMYNSLGV-PVPGMVESIRCLKNAALALMSDEDATEAAPYFDYIIQTMS-------- 161

ApcD1 Chroococcidiopsis thermalis PCC 7203 (WP_015152396.1) GVREMYNSLGV-PVPGMVESIRCLKNASLSLLSAEEAAEAAPYFDYIIQAMS-------- 161

ApcD1 Nostoc sp. PCC 7524 (WP_015139163.1) GVREMYNSLGV-PVPGMVEAINALKKASLDLLSAEDAAEAAPYFDYIIQAMS-------- 161

ApcD1 Anabaena sp. PCC 7120 (WP_010997797.1) GVREMYNSLGV-PVPGMVEAINSLKKASLDLLSSEDAAAAAPYFDYIIQAMS-------- 161

ApcD1 Calothrix sp. PCC 7507 (WP_015130965.1) GVREMYNSLGV-PVPGMVEAINSLKKASLDLLSTEDAAETAPYFDYIIQAMS-------- 161

ApcD1 Chlorogloeopsis fritschii PCC 9212 (WP_016878526.1) GVREMYNSLGV-PVPGMVEAINCLKKASLDLLNAEDAAEAAPYFDYIIQAMS-------- 161

ApcD1 Nostoc sp. PCC 7107 (WP_015113750.1) GVREMYNSLGV-PVPGMVEAINSLKKASLDLLSAEDAAAASPYFDYIIQAMS-------- 161

ApcD2 Synechococcus sp. PCC 7335 (WP_006455320.1) GVREMYLSLEV-PLRSVALCMRSLKEVTLAMLSREDAAEVGPYFDYLIAGLMP------- 159

ApcD2 Leptolyngbya sp. JSC-1 (IMG: 2022833634) GAKEMYASLGV-PLTNLVECMRCLKEVALELLALDDAVEVAPYFDYLIQGLKP------- 159

ApcD2 Chroococcidiopsis thermalis PCC 7203 (WP_015153115.1) GVKEMYESLEI-PLRNWVECIRCLKEVTLDLLSREDAAEVTPYFDCLIQGMIP------- 159

ApcD2 Calothrix sp. PCC 7507 (WP_015126588.1 GVQEMYNSLEI-PLANLVIAIRCLKEVSLDLFNLEDAAELAPYFDYLIQELTP------- 159

ApcD2 Chlorogloeopsis sp. PCC 9212 (WP_016873422.1) GVKEMYDSLEI-PLPNLVEAIRCLKEVSLDLFTLEDAAEVAPYFDYLIQSLMP------- 159

ApcD2 Fischerella thermalis PCC 7521 (WP_009453700.1) GVKEMYESLEI-PLPNLVEAIRCLKEVSLDLFTLEDATEIAPYFDYLIQSLMP------- 159

ApcD3 Synechococcus sp. PCC 7335 (WP_006456515.1) GMKELYTNIGI-PLDNILQYLRCLKAEAIALLSEAEAEAIIPYFDQIIQELVRPGPSYFG 166

ApcD3 Leptolyngbya sp. JSC-1 (MBF2050150.1) GMKEMYISLGI-PLANWVEAVQCLKEEAIALLGQPDAAVVAPYFDHIIQTLALPGTPYFV 166

ApcD3 Calothrix sp. PCC 7507 (WP_015126586.1) GMKEMYASLGI-PIFNWVEAVRCIKEAAREFLGEEDAAIAIPYFDHIIQTLASPGSPYFM 166

ApcD3 Chlorogloeopsis sp. PCC 9212 (WP_016873424.1) GMQQMYESLGI-PLSNWVEAVRCIKEEAQALLGDEDAAEVTPYFDHIIQTLSFPGAPYFM 166

ApcD3 Fischerella thermalis PCC 7521 (WP_009453698.1) GMQQMYQSLGI-PLSNWVEAVRCIKEEAQALLGDEDAAEVTPYFDYIIQALSFPGAPYFM 166

ApcD3 Chroococcidiopsis thermalis PCC 7203 (WP_015153117.1) GMKELYNSVGI-PLENVRQYMLCVKAEVSAMLTPEDAAEVIPYFDLILQVISSPGAPYFQ 166

ApcD4 Synechococcus sp. A1463 (WP_099812040.1) GMREMYISLGV-PLPNLRVAMSCLKEVAAGILSSEEMALAAPYFDRLIRAF--------- 157

ApcD4 Chroococcidiopsis sp. FACHB-1243 (WP_192159074.1) GMREMYVSLGV-PVSNIGSCMRSLKEVATGLMSREEADLVKPYFDYLIRAMY-------- 158

ApcD4 Chroococcidiopsis thermalis PCC 7203 (WP_015156293.1) GMREMYVSLGV-PVSNIGSCMRSLKEVATGLMSREEADLVKPYFDYLIRAMY-------- 158

ApcD4 Gloeocapsa sp. PCC 7428 (WP_015190166.1) GMREMYTSLGV-PVSNIGNCMRCLKEVATNMMSSEEAAIAKPYFDYLIRAMY-------- 158

ApcD4 Chlorogloeopsis sp. PCC 9212 (WP_016874155.1) GMREMYTSLGV-PVSNIGTCMRCLKEVASGMMNREEAAIAGPYFDYLIRAMY-------- 158

ApcD4 Halomicronema hongdechloris (WP_080809693.1) GLRESYVSLGVLPLSYHKVAYRCIKEVAMEILTAEEGALVAPYFDQLIRAF--------- 158

ApcD4 Leptolyngbya sp. PCC 6406 (WP_008312402.1) GMRDMYLSLGV-PLSNLKLAMKCLKDVAIGLLTAEEGALAAPYFEQLIRAF--------- 157

ApcD4 Xenococcus sp. PCC 7305 (WP_006512043.1) GMRDMYVSLQV-PLANLKIAMRCIKSVATGLLNSEEAALAAPYFDELIRAF--------- 157

ApcD4 Pleurocapsa sp. CCALA 161 (WP_106232622.1) GMRDMYTSLQV-PLANLKLAMRCLKSVAMGLLTSEEAALAGPYFDQLIRAF--------- 157

ApcD4 Synechococcus sp. PCC 7335 (WP_006454943.1) GMRDMYVSLNV-PLTNLKTAMRCIKQSAMGVLSSEEASLAGPYFDQLIRAF--------- 157

ApcD4 Romeria gracilis (WP_193906454.1) GMRDMYVSLNV-PLANLKLAMRCLKQVATGLLSSEEAALASPYFDQLIRAF--------- 157

ApcD4 Gloeomargarita lithophora (WP_071455395.1) GMREMYVSLGV-PLANLRMAMGSLKDVAAGLMSGEEMALAAPYFDRLIRAF--------- 157

ApcD5 Synechococcus sp. PCC 7335 (WP_006456289.1) GIKEMYNNLEI-PIRNIAECMRCLKEEAMAVLSDEDAQEVAAYFDLIIQSLS-------- 158

ApcD5 Chroococcidiopsis thermalis PCC 7203 (WP_015153113.1) GIKEMYNNLEI-PLRNIVECMRCLKEEALSLMSEEDALEVSAYFDYVMRSLS-------- 158

ApcD5 Leptolyngbya sp. JSC-1 (IMG: 2022833632) GIKEMYNSLEI-PLKNLVEAMRCVKDEAISLMSEDDAVEVAPYFDYIIRALS-------- 158

ApcD5 Calothrix sp. PCC 7507 (WP_015126590.1) GIKEMYNNLEI-PLRNIVEAMRCVKEEAISLMSEEDAVEVGPYFDYIIRALS-------- 158

ApcD5 Chlorogloeopsis sp. PCC 9212 (WP_016873420.1) GIKEMYNNLEI-PLRNIVEAMRCIKEEAVSMMSEEDAVEVGPYFDYIIRALS-------- 158

ApcD5 Fischerella thermalis PCC 7521 (WP_009453702.1) GIKEMYNNLEI-PLRNIVEAMRCIKEEAVSMMSEEDAVEVGPYFDYIIRALS-------- 158

* :: * : : :* .: . **: : :

ApcA1 Anabaena sp. PCC 7120 (WP_010994626.1) --------------- 161

ApcA1 Synechococcus sp. PCC 6301 (WP_011243498.1) --------------- 161

ApcA1 Synechococcus sp. PCC 7335 (WP_006456136.1) --------------- 161

ApcA1 Synechocystis sp. PCC 6803 (WP_010872503.1) --------------- 161

ApcA1 Synechococcus sp. PCC 7002 (WP_012307540.1) --------------- 161

ApcA1 Calothrix sp. PCC 7507 (WP_015126817.1) --------------- 161

ApcA1 Thermosynechococcus vestitus BP-1 (WP_011056801.1) --------------- 171

ApcA1 Anabaena sp. PCC 7120 (WP_010994198.1) --------------- 161

ApcA1 Chlorogloeopsis sp. PCC 9212 (WP_026087462.1) --------------- 161

ApcA1 Chroococcidiopsis thermalis PCC 7203 (WP_015156256.1) --------------- 161

ApcA1 Fischerella thermalis PCC 7521 (WP_009457007.1) --------------- 161

ApcA1 Leptolyngbya sp. JSC-1 (WP_036003991.1) --------------- 161

ApcD1 Synechococcus sp. A1463 (WP_011431562.1) --------------- 161

ApcD1 Synechocystis sp. PCC 6803 (WP_010871516.1) --------------- 161

ApcD1 Synechococcus sp. PCC 7002 (WP_012307741.1) --------------- 161

ApcD1 Leptolyngbya sp. PCC 7376 (WP_015135290.1) --------------- 161

ApcD1 Synechococcus sp. PCC 6301 (WP_011243585.1) --------------- 163

ApcD1 Thermosynechococcus sp. CL-1 (QEQ1666.1) --------------- 171

ApcD1 Arthrospira platensis NIES46 (GCE93786.1) --------------- 161

ApcD1 Synechococcus sp. PCC 7335 (WP_006454063.1) --------------- 162

ApcD1 Leptolyngbya sp. JSC-1 (WP_036003919.1) --------------- 161

ApcD1 Chroococcidiopsis thermalis PCC 7203 (WP_015152396.1) --------------- 161

ApcD1 Nostoc sp. PCC 7524 (WP_015139163.1) --------------- 161

ApcD1 Anabaena sp. PCC 7120 (WP_010997797.1) --------------- 161

ApcD1 Calothrix sp. PCC 7507 (WP_015130965.1) --------------- 161

ApcD1 Chlorogloeopsis fritschii PCC 9212 (WP_016878526.1) --------------- 161

ApcD1 Nostoc sp. PCC 7107 (WP_015113750.1) --------------- 161

ApcD2 Synechococcus sp. PCC 7335 (WP_006455320.1) --------------- 159

ApcD2 Leptolyngbya sp. JSC-1 (IMG: 2022833634) --------------- 159

ApcD2 Chroococcidiopsis thermalis PCC 7203 (WP_015153115.1) --------------- 159

ApcD2 Calothrix sp. PCC 7507 (WP_015126588.1 --------------- 159

ApcD2 Chlorogloeopsis sp. PCC 9212 (WP_016873422.1) --------------- 159

ApcD2 Fischerella thermalis PCC 7521 (WP_009453700.1) --------------- 159

ApcD3 Synechococcus sp. PCC 7335 (WP_006456515.1) IKDRSARQSARQAAA 181

ApcD3 Leptolyngbya sp. JSC-1 (MBF2050150.1) NDGTSEY-------- 173

ApcD3 Calothrix sp. PCC 7507 (WP_015126586.1) NDGRTDW-------- 173

ApcD3 Chlorogloeopsis sp. PCC 9212 (WP_016873424.1) NDGRLDW-------- 173

ApcD3 Fischerella thermalis PCC 7521 (WP_009453698.1) NDGRSDW-------- 173

ApcD3 Chroococcidiopsis thermalis PCC 7203 (WP_015153117.1) NNGRTDWQR------ 175

ApcD4 Synechococcus sp. A1463 (WP_099812040.1) --------------- 157

ApcD4 Chroococcidiopsis sp. FACHB-1243 (WP_192159074.1) --------------- 158

ApcD4 Chroococcidiopsis thermalis PCC 7203 (WP_015156293.1) --------------- 158

ApcD4 Gloeocapsa sp. PCC 7428 (WP_015190166.1) --------------- 158

ApcD4 Chlorogloeopsis sp. PCC 9212 (WP_016874155.1) --------------- 158

ApcD4 Halomicronema hongdechloris (WP_080809693.1) --------------- 158

ApcD4 Leptolyngbya sp. PCC 6406 (WP_008312402.1) --------------- 157

ApcD4 Xenococcus sp. PCC 7305 (WP_006512043.1) --------------- 157

ApcD4 Pleurocapsa sp. CCALA 161 (WP_106232622.1) --------------- 157

ApcD4 Synechococcus sp. PCC 7335 (WP_006454943.1) --------------- 157

ApcD4 Romeria gracilis (WP_193906454.1) --------------- 157

ApcD4 Gloeomargarita lithophora (WP_071455395.1) --------------- 157

ApcD5 Synechococcus sp. PCC 7335 (WP_006456289.1) --------------- 158

ApcD5 Chroococcidiopsis thermalis PCC 7203 (WP_015153113.1) --------------- 158

ApcD5 Leptolyngbya sp. JSC-1 (IMG: 2022833632) --------------- 158

ApcD5 Calothrix sp. PCC 7507 (WP_015126590.1) --------------- 158

ApcD5 Chlorogloeopsis sp. PCC 9212 (WP_016873420.1) --------------- 158

ApcD5 Fischerella thermalis PCC 7521 (WP_009453702.1) --------------- 158

**B**

ApcE subunits (E1 and E2)

ApcE1_Synechococcus_sp_PCC_7335_WP_006453887.1 MTVTASGGSSVARPQLYQTLPASTISQAEQKDRYMENTELGELKTFFNSGMKRVAIAQTL 60

ApcE_Synechococcus_7002_WP_012307618.1 MTIKASGGSSLARPQLYQTVPLSNISQAEQQDRYLESGELTALKTFYDSGLKRLAIAQAI 60

ApcE_Synechocystis_6803_WP_010873271.1 MSVKASGGSSLARPQLYQTVPVSAISQAEQQDRFLEGSELNELTAYFQSGALRLEIAETL 60

ApcE1_Calothrix_sp_PCC_7507_WP_015126818.1 MSVKASGGSSVARPQLYQTLAVATISQAEQQDRFLGTGELNELASYFASGAKRLEIAQTL 60

ApcE1_Chroococcidiopsis_thermalis_PCC_7203_WP_015156255.1 MSVKASGGSSVARPQLYQTLAVATISQAEQQDRFLGRGELDELANYFASGNKRLEIAETL 60

ApcE1_Chlorogloeopsis_sp_PCC_9212_WP_016874650.1 MSVKASGGSSVARPQLYQTLAVSTISQAEQQDRFLGAGELSELANYFASGARRLEIAQIL 60

ApcE1_Fischerella_thermalis_PCC_7521_WP_009457006.1 MSVKASGGSSVARPQLYQTLAVSTISQAEQQDRFLGAGELNELANYFASGAKRLEIAQTL 60

ApcE2_Synechococcus_sp_PCC_7335 MTDRTNGGSPVVHPQQYHTVPTAVINGAHQRDRYPNHSEMQTLSTFLRTGLQRLEIAQTL 60

ApcE2_Chroococcidiopsis_thermalis_PCC_7203_WP_015153116.1 MSVKASGGSPVTQPQRYHTVPVAVISHAVQQDRCLKNTELQELADFFSSGVKLLEIANTL 60

ApcE2_Calothrix_sp_PCC_7507_WP_015126587.1 MSIKASGGSPVVYPQRYQTVPVALISVAEQQDRCLKHTELQELGSFFSSGNQRLEIAETL 60

ApcE2_Chlorogloeopsis_sp_PCC_9212_WP_016873423.1 MSAKASSNSSVAHPQQYQTVPIAVISQAEQQDRCLKRTELQVLNSFFSSGNKRLEIVETL 60

ApcE2_Fischerella_thermalis_PCC_7521_WP_009453699.1 MSAKASSSISVAHPQLYQTVPIAVISQAEQQDRCLKRTELQELNSFFSSGNKRLEIVETL 60

*: :... :. ** *:*: : *. * *:** *: * : :* : *.: :

ApcE1_Synechococcus_sp_PCC_7335_WP_006453887.1 TRYSELIVSQAANRIFTGGSALAYLEKSADDAPQEMTRG-GVPLDQKEASK--LGTATF- 116

ApcE_Synechococcus_7002_WP_012307618.1 KLSSQLIVSRAANRIFAGGSPLAYLDQPETDTDDS---------DLGVSM-------AV- 103

ApcE_Synechocystis_6803_WP_010873271.1 TQNADLIVSRAANRIFTGGSPLSYLEKPVERQPALVGAS--------SDSR--NGSVTY- 109

ApcE1_Calothrix_sp_PCC_7507_WP_015126818.1 TENSEIIVSRAANRIFVGGSPMAFLEKPKEPELVLAS---VGGGDVQEGMK--LGTVTY- 114

ApcE1_Chroococcidiopsis_thermalis_PCC_7203_WP_015156255.1 TKNSEIIVSRAANRIFVGGSPMAYLEKPRETEMAMA----ATVPDVKQGMQ--LGTITY- 113

ApcE1_Chlorogloeopsis_sp_PCC_9212_WP_016874650.1 TDNSEIIVSRAANRIFVGGSPMAFLEKPREPEVAMAVAAAASSSDVRDAMK--LGTVTY- 117

ApcE1_Fischerella_thermalis_PCC_7521_WP_009457006.1 TDNSEIIVSRAANRIFVGGSPMAFLEKPREPELAMAVA--ASTSDVRDAMK--LGTVTY- 115

ApcE2_Synechococcus_sp_PCC_7335 AQHANEIVAAGGKRIFVGGNPMAYFEQPEELVGMPGS-GYFVAEDYLSPKSRRQTGNG-- 117

ApcE2_Chroococcidiopsis_thermalis_PCC_7203_WP_015153116.1 TQHADEIVLAGANRIFVGGSPMAYLEKPKEKIGLPGS-GYYVGEDFLTAARRKAGAVMVK 119

ApcE2_Calothrix_sp_PCC_7507_WP_015126587.1 TRNADEIVAAGANRIFVGGSPMAYLEKPQDPVGLPGS-GYYVAEDYLTAARK-SGVAPGK 118

ApcE2_Chlorogloeopsis_sp_PCC_9212_WP_016873423.1 TKNADEIVSVGANRIFVGGFPMDYLEKPQDPLGLPGS-GYYVGEDFLSAARR-NGYVPDQ 118

ApcE2_Fischerella_thermalis_PCC_7521_WP_009453699.1 TKNADEIVSVGANRIFVGGFPMDYLEKPQDPLGLPGS-GYYVGEDYLSAARR-NGYVPDK 118

:: ** ..:***.** : ::::

ApcE1_Synechococcus_sp_PCC_7335_WP_006453887.1 ----VAANSDGNGSRGGIFSGLRNLLINDPD---AGVTPPNFRPINVARYGPSNMQKSLR 169

ApcE_Synechococcus_7002_WP_012307618.1 ----GDAS-----GATGIFGGVKNLFL----GSGGGKIPAGFRPISVSRYGPRNMTKSLR 150

ApcE_Synechocystis_6803_WP_010873271.1 ----AESN-----GSGGLFGGLRSVFSST------GPIPPGFRPINIARYGPSNMQKSLR 154

ApcE1_Calothrix_sp_PCC_7507_WP_015126818.1 ----VESK-----G--GFLENLRSIFNTSAG----GPTPAGFRPINIARYGPSNMAKSLR 159

ApcE1_Chroococcidiopsis_thermalis_PCC_7203_WP_015156255.1 ----VESR-----G-GGFLEGLRSLFSASPGGGGGGPTPPGFRPINVARYGPGNMQKSLR 163

ApcE1_Chlorogloeopsis_sp_PCC_9212_WP_016874650.1 ----VETR-----G--GFLENLRSIFNSSPS----GPTPPGFRPINIARYGPANMAKSLR 162

ApcE1_Fischerella_thermalis_PCC_7521_WP_009457006.1 ----VETR-----G--GFLENLRSIFNSSPS----GPIPPGFRPINIARYGPANMAKSLR 160

ApcE2_Synechococcus_sp_PCC_7335 --HSVQNSSSSITNPVAWLKG--LFFSGK------PSVPSRFQAINIADYGAVRMKRSMR 167

ApcE2_Chroococcidiopsis_thermalis_PCC_7203_WP_015153116.1 EALKIQEVAYYSNPLSGWLQRFRDLFNNQ------DPLPGGFRFINVSRYGAVRMKRSMR 173

ApcE2_Calothrix_sp_PCC_7507_WP_015126587.1 ERLNLAENLNFFNPLSGWWERARTLLTDR------EPLPGGFRFINISRYGPIRMKRSMR 172

ApcE2_Chlorogloeopsis_sp_PCC_9212_WP_016873423.1 ERVNLLPTPRFFNPLSAWWEQARTLFTDR------EPLPEGFRFINISRYGPTRMKRSMR 172

ApcE2_Fischerella_thermalis_PCC_7521_WP_009453699.1 ERVNLIPTPRFFNPLRGWWEQARTLFTDR------DPLPEGFRFINISRYGPTRMKRSMR 172

. .: * *: *.:: ** .* :*:*

ApcE1_Synechococcus_sp_PCC_7335_WP_006453887.1 DMSWFLRYLTYAVVAGDPNILKVNVRGLREIIENACSTPATIVAIQTMRGASVGYFKGDP 229

ApcE_Synechococcus_7002_WP_012307618.1 DMAWFLRYTTYAIVAGDPSILVVNTRGLKEVIENACSIPATIVAIQEMKAASLDLFRGDR 210

ApcE_Synechocystis_6803_WP_010873271.1 DMSWFLRYTTYAIVAGDPNIIVVNTRGLKEVIENACSIDATIVAIQEMRAASADYFRNNA 214

ApcE1_Calothrix_sp_PCC_7507_WP_015126818.1 DLSWFLRYATYAIVAGDPNIIAVNTRGLREIIENACSGEATLVALQEIKVGALSFFRKDA 219

ApcE1_Chroococcidiopsis_thermalis_PCC_7203_WP_015156255.1 DLSWFLRYATYAIVAGDPNIISVNVRGLREIIENACSGEATIVALQEMRASALSYFRQNP 223

ApcE1_Chlorogloeopsis_sp_PCC_9212_WP_016874650.1 DLSWFLRYATYAIVAGDPNIIAVNTRGLREIIENACSGEATIVALQEMKAAALSYFRQDA 222

ApcE1_Fischerella_thermalis_PCC_7521_WP_009457006.1 DLSWFLRYATYAIVAGDPNIIAVNTRGLREIIENACSGEATIVALQELKAAALSYFRKDT 220

ApcE2_Synechococcus_sp_PCC_7335 DLGWFLRYITYAVVAGDTSIITVNTRGLRGIIPEDV-TVATTVALQEMQWKSLSFFPVDS 226

ApcE2_Chroococcidiopsis_thermalis_PCC_7203_WP_015153116.1 DLAWFLRYITYAIVAGDGSILSANVRGLRGVIPEDV-TEATIVALRAMRRQSLDYFLEDA 232

ApcE2_Calothrix_sp_PCC_7507_WP_015126587.1 DLAWFLRYVTFAIVAGDDSILKVNVRGLRGVIPEDV-TEATVVALKEMQRLSLSYFPQNA 231

ApcE2_Chlorogloeopsis_sp_PCC_9212_WP_016873423.1 DLAWFLRYITFAIVAGDTSILSANARGLRGVIPEDV-TDATVVALKEMQRQSLSYFANDA 231

ApcE2_Fischerella_thermalis_PCC_7521_WP_009453699.1 DLAWFLRYITYAIVAGDTSILSANARGLRGIIPEDV-TDATVVALKEMQRQSLNYFANDA 231

*:.***** *:*:**** .*: .*.***: :* : ** **:: :: : . * :

ApcE1_Synechococcus_sp_PCC_7335_WP_006453887.1 EAQDILRQYFDIMLSEFKGPTPSKKLRQRSSKDQQGLQLPQIYFNASERRPKYAMKPGLS 289

ApcE_Synechococcus_7002_WP_012307618.1 EAQETVVQYFDVLITEMQTQVPNDKLRQRPSIDAQGLQLPQSYFNAAEKRQKFVMKPGLS 270

ApcE_Synechocystis_6803_WP_010873271.1 QAKEIVLQYFDILLSEFKAPTPANKVRQGPSNDIQGLELPQSYFNAAAKRQKYAMKPGLS 274

ApcE1_Calothrix_sp_PCC_7507_WP_015126818.1 EATEIVSQYMDVLLTEFQAPTPSTKLRQRPSSDQQGLQLPQIYFNSAERRPKFVLKAGSS 279

ApcE1_Chroococcidiopsis_thermalis_PCC_7203_WP_015156255.1 EAANIVAQYMDVLITEFKAPTPSNKLRQRPSSDQQGLQLPQIYFNAAERRPKYAMKPGLS 283

ApcE1_Chlorogloeopsis_sp_PCC_9212_WP_016874650.1 EAADIVSQYMDVLITEFKAPSPSNKLRQRPSGDQQGLQLPQIYFNAAERRQKFVMKPGLS 282

ApcE1_Fischerella_thermalis_PCC_7521_WP_009457006.1 EAADIVSQYMDVLITEFKAPTPSNKLRQRPSGDQQGLELPQIYFNAAERRQKFVMKPGLS 280

ApcE2_Synechococcus_sp_PCC_7335 AAAALVRRYFDVLIADYQVEKPSDRYRTGVSKHDQGLSFPESYEDSGCAIPRWVMKPTLP 286

ApcE2_Chroococcidiopsis_thermalis_PCC_7203_WP_015153116.1 EATQLVKGYFDLLIAEYLTDKPSNQVRIGVSNDQQGLQLPQSYSMSAEVRPKFVFKQAAT 292

ApcE2_Calothrix_sp_PCC_7507_WP_015126587.1 EASELIKQSFETLITEYLAEKPSVQLRVGVSNEQQGLVLPQSYAIAAEHRLKFVIKSVLS 291

ApcE2_Chlorogloeopsis_sp_PCC_9212_WP_016873423.1 EAQEIIKHNFEVLISEYLVEKPPAQLRIGVSNEQQGLVLPQSYAIASLVRLKYVMKSVLP 291

ApcE2_Fischerella_thermalis_PCC_7521_WP_009453699.1 EAQEIIKHNFQVLISEYLVEKPPIELRIGVSDEQQGLVLPQSYAIASLVRLKYVMKSVLP 291

* : :: :::: * . * * . *** :*: * :. ::.:*

|||

ApcE1_Synechococcus_sp_PCC_7335_WP_006453887.1 RLEKNEAIKAAYRQVFERDITRAYSQSI-SDLESKVRNGEISMKEFIRRLAKSPLYRRQF 348

ApcE_Synechococcus_7002_WP_012307618.1 ALEKNSVVKAAYRQIFERDITRAYSQSI-SYLESQVKSGDISMKEFVRRLAKSPLYRKQF 329

ApcE_Synechocystis_6803_WP_010873271.1 ALEKNAVIKAAYRQIFERDITKAYSQSI-SYLESQVRNGDISMKEFVRRLAKSPLYRKQF 333

ApcE1_Calothrix_sp_PCC_7507_WP_015126818.1 ATEKNEVIKATYRQIFERDITRAYSLSI-SDLESKVKNGDISVKEFVRRLAKSPLYQKQF 338

ApcE1_Chroococcidiopsis_thermalis_PCC_7203_WP_015156255.1 AAEKNDIVKAAYRQVFERDITRAYSQSI-SYLESQVKNGDISMKEFIRRLAKSPLYRKQF 342

ApcE1_Chlorogloeopsis_sp_PCC_9212_WP_016874650.1 AAEKTEVVKAAYRQIFERDITRAYSLSI-SYLESQVKNGDISMKEFVRRLGKSPLYRKQF 341

ApcE1_Fischerella_thermalis_PCC_7521_WP_009457006.1 AAEKTEVVKAAYRQIFERDITRAYSLSI-SYLESQVKNGDISMKEFVRRLAKSPLYRKQF 339

ApcE2_Synechococcus_sp_PCC_7335 DSEKDAVIRAAYRQVFERDISGL-GTAELTQPISQLKGEDGSMELFIRQLGKSRLYRQLF 345

ApcE2_Chroococcidiopsis_thermalis_PCC_7203_WP_015153116.1 LTQKQEAIAAIYRHVFERDVTDTYGFTQKAELESQLIGGNISVKEFVRRLGKSRLYRRLF 352

ApcE2_Calothrix_sp_PCC_7507_WP_015126587.1 ETEKQAAIKAAYRQVFERDITATYDFPA-SELESQVKGGQISTKEFIRRLGKSRLYRRLF 350

ApcE2_Chlorogloeopsis_sp_PCC_9212_WP_016873423.1 ETEKQAVIKAAYRQVFERDVTASYGFAV-DELESQVKGGQISMKEFVRRLGKSRLYRRLF 350

ApcE2_Fischerella_thermalis_PCC_7521_WP_009453699.1 ETEKQAVIKAAYRQVFERDVAATYGFPV-DELESQVKGGQISMKEFVRRLGKSRLYRRLF 350

:* : * **::****:: . *:: . : * : *:*:*.** **:: *

|||

ApcE1_Synechococcus_sp_PCC_7335_WP_006453887.1 FEPFINSRALELAFRHILGRGPSSREEVQKYFAIVSEGGLSKLVDALVDSQEYADYFGEE 408

ApcE_Synechococcus_7002_WP_012307618.1 FEPFINSRALELAFRHILGRGPSSREEVQEYFAIVSSGGLAALVDALVDSQEYADYFGEE 389

ApcE_Synechocystis_6803_WP_010873271.1 FEPFINSRALELAFRHILGRGPSSREEVQKYFSIVSSGGLPALVDALVDSQEYADYFGEE 393

ApcE1_Calothrix_sp_PCC_7507_WP_015126818.1 YQPFINSRVIELAFRHILGRGPSSREEVQKYFAIISKGGLAALVDALVDSAEYSDYFGEE 398

ApcE1_Chroococcidiopsis_thermalis_PCC_7203_WP_015156255.1 YEPFINSRALELAFRHILGRGPSSREEVQKYFSIVSNGGLSALIDALVDSQEYSDYFGEE 402

ApcE1_Chlorogloeopsis_sp_PCC_9212_WP_016874650.1 YEPFINSRALELAFRHFLGRGPSSREEVQKYFDIVSKGGLAALIDALVDSEEYSDYFGEE 401

ApcE1_Fischerella_thermalis_PCC_7521_WP_009457006.1 YEPFINSRALELAFRHILGRGPSSREEVQKYFDIVSRGGLSALVDALVDSDEYSDYFGEE 399

ApcE2_Synechococcus_sp_PCC_7335 YEPYMISRSIELACRHFLGRGLSCMEEFQRYFELVADQGFSALVDALVSSQEYADYFGAE 405

ApcE2_Chroococcidiopsis_thermalis_PCC_7203_WP_015153116.1 YEPFTISRAIELAARHFLGRGLSSREEFQTYFDVMTKGGLPALVDAFVDSAEYSDYFGEE 412

ApcE2_Calothrix_sp_PCC_7507_WP_015126587.1 YEPFTISRVIELAMRHFLGRGLSSLAEFQGYFAIITKGGLHKLIDALVDSSEYADYFGEE 410

ApcE2_Chlorogloeopsis_sp_PCC_9212_WP_016873423.1 WEPYTISRVIELAMRHFLGRGLSSLEEFQAYFAVVTKGGLPKLIDTLVDSQEYADYFGEE 410

ApcE2_Fischerella_thermalis_PCC_7521_WP_009453699.1 WEPYTISRVIELAMRHFLGRGLSSLEEFQEYFAVVTKGGLPKLVDTLVDSQEYADYFGEE 410

::*: ** :*** **:**** *. *.* ** ::: *: *:*::*.* **:**** *

||| ||| ||

ApcE1_Synechococcus_sp_PCC_7335_WP_006453887.1 TVPYIRGLGQEAQECRNWGAQQELFNYSAPYRKIPQFVTLFASYNQPLPDQHVYGSGNDP 468

ApcE_Synechococcus_7002_WP_012307618.1 TVPYLRGLGQEAQECRNWGMQQDLFKYSAPFRKVPQFITTFASYNQPLPDQHVYGSGNDA 449

ApcE_Synechocystis_6803_WP_010873271.1 TVPYLRGLGVEAQECRNWGMQQDLFSYSAPFRKVPQFITTFAQYDRPLPDQHVYGSGNDP 453

ApcE1_Calothrix_sp_PCC_7507_WP_015126818.1 TVPYIRGLGQEAQECRNWGLQQDLFNYSAPFRKIPQFITTFAAYDRPLPDQHPYGSGNDP 458

ApcE1_Chroococcidiopsis_thermalis_PCC_7203_WP_015156255.1 TVPYIRGLGQEAQECRNWGPQQDLLNYSAPFRKVPQFITTFAAYNRPLPDQHPYGSGNDP 462

ApcE1_Chlorogloeopsis_sp_PCC_9212_WP_016874650.1 TVPYIRGLGQEAQECRNWGPQQDLFKYSAPFRKVPQFLTTFADYEQPLPDQHPYGSGNDP 461

ApcE1_Fischerella_thermalis_PCC_7521_WP_009457006.1 TVPYIRGLGQEAQECRNWGPQQDLFKYSAPFRKVPQFITTFADYNQPLPDQHPYGSGNDP 459

ApcE2_Synechococcus_sp_PCC_7335 TVPYIRGLGIEAQACRNWGPQLDLFKYSAPARKVPQFVTAFASYRQPLPNQHPYGMGNDP 465

ApcE2_Chroococcidiopsis_thermalis_PCC_7203_WP_015153116.1 TVPYLRGLGQEAQECRNWGPQLDLFKYSAPVRKVPQFITLFGSYQKPLPEQHPYGCGNDP 472

ApcE2_Calothrix_sp_PCC_7507_WP_015126587.1 TVPYLRGLGQEAQECCNWGPQLELFKYSAPVRKVPQFLTLFGKYQKPLPNQHPYGSGNDP 470

ApcE2_Chlorogloeopsis_sp_PCC_9212_WP_016873423.1 TVPYLRGLGQEAQECRNWGPQIDLFKYSAAVHKVPQFVTLFGKYTKPLPNQHPYGSGNDP 470

ApcE2_Fischerella_thermalis_PCC_7521_WP_009453699.1 TVPYLRGLGQEAQECRNWGPQIDLFKYSTVVHKVPQFVTLFGKYTKPLPNQHPYGSGNDP 470

****:**** *** * *** * :*:.**: :*:***:* *. * :***:** ** ***

|

ApcE1_Synechococcus_sp_PCC_7335_WP_006453887.1 LEIQFGAIFPKETRDP--SSSPAPFSKDTRRILIHQGAGINNQLSNPAARPVAPGSLGAK 526

ApcE_Synechococcus_7002_WP_012307618.1 LEIQFGAIFPKATRSP--SASPAPFNKDTRRILIHRGPGINNQLGNPRARATQPGSLGAK 507

ApcE_Synechocystis_6803_WP_010873271.1 LEIQFGAIFPKETRNP--SKRPAPFNKDTKRILIHRGPAVNNQVGNPSAVGEFPGSLGAK 511

ApcE1_Calothrix_sp_PCC_7507_WP_015126818.1 LEIQFGAIFSKETRNP--STSPAPFAKDTKRILIHQGPGINNQNSNPQARGAAPGSLGPK 516

ApcE1_Chroococcidiopsis_thermalis_PCC_7203_WP_015156255.1 LEIQFGAIFPKETRNP--SSSPAPFGKDTKRILIHQGPGINNQNSNPRARGEFPGTLGAK 520

ApcE1_Chlorogloeopsis_sp_PCC_9212_WP_016874650.1 LEIQFGAIFPKETRNP--SSRPAPFGKDTRRILIHQGPATNNQTGNPSARGEFPGTLGAK 519

ApcE1_Fischerella_thermalis_PCC_7521_WP_009457006.1 LEIQFGAIFPKETRNP--SSRPAPFGKDTKRILIHQGPAINNQNSNPAARGEFPGSLGPK 517

ApcE2_Synechococcus_sp_PCC_7335 LETQFGAIFPHETTNP--AAQPVHFSEDSRRILVGHAHRKSHA------------EISQQ 511

ApcE2_Chroococcidiopsis_thermalis_PCC_7203_WP_015153116.1 LEIQFGAIFPQETRNP--HPQPAFFNKDTRRILIGSGAGSPDKL-NGNALGKVPGSLGTR 529

ApcE2_Calothrix_sp_PCC_7507_WP_015126587.1 LEIQFGAIFPVDTLPATTLHSPAPFGKDNRRILISS-----------EGFGQVPGYLGRL 519

ApcE2_Chlorogloeopsis_sp_PCC_9212_WP_016873423.1 LEIQFGAIFPVDTLPATTLHSPAPFGRDNRRILISS-----------NGLGQVPGTLGRV 519

ApcE2_Fischerella_thermalis_PCC_7521_WP_009453699.1 LEIQFGAIFPVDTLPATTLHSPAPFGKDNRRILISS-----------NGLGQVPGTLGKI 519

** ****** * *. * .*.:***: :.

ApcE1_Synechococcus_sp_PCC_7335_WP_006453887.1 VFKS-------------------------SEASKQTIISAVYRQVFGRPVYAGQEI--SK 559

ApcE_Synechococcus_7002_WP_012307618.1 VFRLNNELP-----------SGKTTNVSFSESATQKVIEAAYRQVFGRMVYAGQRQ--KV 554

ApcE_Synechocystis_6803_WP_010873271.1 VFRLNGGLPGAK------VGKNTGTSVKFGESSTQALIRAAYRQVFGRDLYEGQRL--SV 563

ApcE1_Calothrix_sp_PCC_7507_WP_015126818.1 VFKLDQVPGTIG------KKVGKGASVKFSESSTQAVIRAIYLQVFGRDVYEGQRQ--KV 568

ApcE1_Chroococcidiopsis_thermalis_PCC_7203_WP_015156255.1 VFRLDQIPATGG------RKSPTTSSVRFSESSTQAVIRAAYLQVFGRDVYEGQRL--KV 572

ApcE1_Chlorogloeopsis_sp_PCC_9212_WP_016874650.1 VFRLDQIPRTLS------KGTGKGASVKYSESSTQAVIRAAYLQVFGRDVYEGQRL--KV 571

ApcE1_Fischerella_thermalis_PCC_7521_WP_009457006.1 VFRLDQIPRTLS------KGTGKGSSVKYSESSTQAVIRGAYLQVFGRDVYEGQRL--KV 569

ApcE2_Synechococcus_sp_PCC_7335 IFSLKTLAH---KPTKASESLSFFPSSDSRQHSVESVILAAYRQVFGCEVLGSQRH--QA 566

ApcE2_Chroococcidiopsis_thermalis_PCC_7203_WP_015153116.1 VLKLEPLHHANGKSNGVTQAGHQSPSVNLLHHSSPAFIEGAYRQVFGRSLYEGQRQPLSS 589

ApcE2_Calothrix_sp_PCC_7507_WP_015126587.1 MKLEQTNHRYGQETTGSSPKLPMGLSVSLTKNSPSAVIEGAYRQIFGRDVFAGQRI--TS 577

ApcE2_Chlorogloeopsis_sp_PCC_9212_WP_016873423.1 IKLDHPEKLHTTSLV---NGKQTPPNVSLTKNSLPAVIQGAYRQVFGRDVFEGQRI--TV 574

ApcE2_Fischerella_thermalis_PCC_7521_WP_009453699.1 IKLDHPEKLHTTSPV---NGKQAPPNISLAKNSPSIVIQVAYRQVFGRDVFEGQRI--TV 574

: . : .* * *:** : .*.

|||

ApcE1_Synechococcus_sp_PCC_7335_WP_006453887.1 AESRFNNDEINVREFVKAVAKSESFRKIYWTSLYVMKAVEYIHRRLLGRPTYGRQETNQY 619

ApcE_Synechococcus_7002_WP_012307618.1 AEIKLENGEITLREFIRALAKSDVFRNTYWSSLYVTKAVEYIHRRLLGRPTYGRQEINSY 614

ApcE_Synechocystis_6803_WP_010873271.1 AEIQLENGDISVREFIKRLAKSELFLKLYWAPHYVCKAIEYMHRRLLGRPTYGRQEMNQY 623

ApcE1_Calothrix_sp_PCC_7507_WP_015126818.1 LEIKLENGEISVREFVRALAKSDLFRSLYWTPLYVCKAIEYIHRRLLGRPTYGRQENNKY 628

ApcE1_Chroococcidiopsis_thermalis_PCC_7203_WP_015156255.1 AEIKLENGEISLREFIRALAKSDLFRKLYWTPFYVCKAIEYIHRRLLGRPTYGRQENNKY 632

ApcE1_Chlorogloeopsis_sp_PCC_9212_WP_016874650.1 QEIKLENGEISVREFIRALAKSDLFRKLYWTPLYVCKAIEYIHRRLLGRPTYGRQENNKY 631

ApcE1_Fischerella_thermalis_PCC_7521_WP_009457006.1 QEIKLENGEISVREFIRALAKSDLFRKLYWTPYYVCKAIEYIHRRLLGRPTYGRQENNKY 629

ApcE2_Synechococcus_sp_PCC_7335 AETQLKGGLITVREFVRQLAKSRSFRQAYWENLYMTKAAEIIHRRLLGRPTYGRRETSKY 626

ApcE2_Chroococcidiopsis_thermalis_PCC_7203_WP_015153116.1 TESKLLGGEISVREFVRQLAKSKVFRSLYWDSLYVTKAIEYIHRRLMGRPTYGRQEMNRY 649

ApcE2_Calothrix_sp_PCC_7507_WP_015126587.1 VESELLSGEITMREFIRQLAKSKLFRKTYWESLYITKAIEYIHRRLLGRPTYGREEMNRY 637

ApcE2_Chlorogloeopsis_sp_PCC_9212_WP_016873423.1 AESALLSGAITMREFIRQLAKSKLFRSMFWEPLYVTKAIEYIHRRLLGRPTYGRQEMNHY 634

ApcE2_Fischerella_thermalis_PCC_7521_WP_009453699.1 AESAFLSGAITMREFIRQLAKSKLFRRMFWEPLYITKAIEYIHRRLLGRPTYGRQEMNHY 634

* : .. *.:***:: :*** * :* *: ** * :****:*******.* . *

ApcE1_Synechococcus_sp_PCC_7335_WP_006453887.1 FDICAKKGFYALIDAIIDSQEYTECFGEDTVPYERYLTPGGQAMRSLRVGSIQE-TGARP 678

ApcE_Synechococcus_7002_WP_012307618.1 FDTCAKKGFYALVDAIIDSKEYEEAFGEDTVPYERYLTPGGYSLRQTRPGALREDVGVKV 674

ApcE_Synechocystis_6803_WP_010873271.1 FDIASKQGFYAVVEAMIDSKEYSDAFGEDTVPYERYLTPGGLQMRSARVGSLREDIGQRV 683

ApcE1_Calothrix_sp_PCC_7507_WP_015126818.1 FDIASKKGFYAVVDAILDTLEYTEAFGEDTVPYERYLTPGGEALRRLRVGSIREDVGGKV 688

ApcE1_Chroococcidiopsis_thermalis_PCC_7203_WP_015156255.1 FDICAKKGFYALVDAIIDSPEYGEAFGEDTVPYERYLTPQGQALRSLRTGSIREDVGARV 692

ApcE1_Chlorogloeopsis_sp_PCC_9212_WP_016874650.1 FDICSKKGFYALVDAIIDSEEYSEAFGEDTVPYERYLTPGGVALRKLRVGSIREDVGAKV 691

ApcE1_Fischerella_thermalis_PCC_7521_WP_009457006.1 FDICSKKGFYALIDAIIDSEEYSQAFGEDTVPYERYLTPGGVALRKLRVGSIREDVGARV 689

ApcE2_Synechococcus_sp_PCC_7335 YDICGRQGFYALVDALIDSDDYRTAFGENTVPYERYVTPRGLALRSPKGPVAISKLRDNP 686

ApcE2_Chroococcidiopsis_thermalis_PCC_7203_WP_015153116.1 YDICATRGFYALIDAIIDSPEYLECFGENTVPYERYVTARGYLMRSPRHENQLRREQAA- 708

ApcE2_Calothrix_sp_PCC_7507_WP_015126587.1 YDICANQGFYALIDEIIDSPEYIQAFGEDTVPYERYVTPRGLLMRQSSVAHSVNMWEKSL 697

ApcE2_Chlorogloeopsis_sp_PCC_9212_WP_016873423.1 YDISANQGFYALIDEIINSPEYMQTFGEDTVPYERYVTPRGFAMRSPKSSYAVNLSAKSL 694

ApcE2_Fischerella_thermalis_PCC_7521_WP_009453699.1 YDISANQGFYALIDEMIDSPEYMQTFGEDTVPYERYVTPRGFAMRSPKSSYAVNLSAKSL 694

:* .. :****::: :::: :* ***:*******:* * :*

ApcE1_Synechococcus_sp_PCC_7335_WP_006453887.1 --EEKTTPRFIELGAVDKN-RPIPEVQQRVNQGVSVQRE----QTKRFKLTT--QEKTAV 729

ApcE_Synechococcus_7002_WP_012307618.1 --KVEKTARFIELGTSSTKNLPVTDVDARLKQGVNIQRQ----QTKAFKLTD-TFNKVEL 727

ApcE_Synechocystis_6803_WP_010873271.1 --DKEVTPRFVELGQVSAI-RTEPEIAYRSNQGVTRQRQ----QTKVFKLVS-TYDKVAV 735

ApcE1_Calothrix_sp_PCC_7507_WP_015126818.1 --QKEATPRFVELGTVTES-RTEPDIQFRINQGVTKQRE----QTKIFKLVANTDDKVAV 741

ApcE1_Chroococcidiopsis_thermalis_PCC_7203_WP_015156255.1 --DKEETPRFVELGAVTEM-RTQPDIQIRVAQGVTKQRE----QTKIFKLEE-NSDKVAV 744

ApcE1_Chlorogloeopsis_sp_PCC_9212_WP_016874650.1 --EKQETPRFVELGTVKES-RTEPDVQFRINQGVSKKRE----QRKIFKLVAGTNDKVAV 744

ApcE1_Fischerella_thermalis_PCC_7521_WP_009457006.1 --DKQETPMFVQMGAVTAT-RTEPDIQARINQGVSKKRE----QRKIFKLVAGTGDKVAV 742

ApcE2_Synechococcus_sp_PCC_7335 HTVGEYMMRYQPPAANISP-RSPLN----------NSASNRQPATARHSDNGALEDRSAS 735

ApcE2_Chroococcidiopsis_thermalis_PCC_7203_WP_015153116.1 ---ETVPDKYNPRKANWAA-LTEFVEQPILNQITSDGRSNRATEMRHAEIVGDRSNSPEE 764

ApcE2_Calothrix_sp_PCC_7507_WP_015126587.1 LSSPSELDRFVPNNRNG--------------LGTGNGGS------------GAIIEIEEV 731

ApcE2_Chlorogloeopsis_sp_PCC_9212_WP_016873423.1 LSSTSELDRFVPNHRNG--------------QTQTLSEL----------VNGCEADVQEQ 730

ApcE2_Fischerella_thermalis_PCC_7521_WP_009453699.1 LSSPSELDRFVPNHRNG--------------QTQTLPEL----------VNGCETNLQEQ 730

: :

ApcE1_Synechococcus_sp_PCC_7335_WP_006453887.1 LTVAQAAY------------------RQIFERNIDPYVVKGSEFSELESKLVNGEINVKE 771

ApcE_Synechococcus_7002_WP_012307618.1 KTAIAAAY------------------RQIFERDIEPYIVD-AQFTALESKLGNREINMKE 768

ApcE_Synechocystis_6803_WP_010873271.1 KNAIRAAY------------------RQVFERDLEPYIIN-SEFTALESKLSNNEINVKE 776

ApcE1_Calothrix_sp_PCC_7507_WP_015126818.1 KTLINAAY------------------RQVFERDVAPYIAK-NEFTVLESKLSNGEISVKE 782

ApcE1_Chroococcidiopsis_thermalis_PCC_7203_WP_015156255.1 QTVIRAAY------------------RQIFERDIEPYIAQ-NEFTALESKLGNGEITVKE 785

ApcE1_Chlorogloeopsis_sp_PCC_9212_WP_016874650.1 QTAISAAY------------------RQIFERDIAPYVASSREFKVLESKLSNGEITVKE 786

ApcE1_Fischerella_thermalis_PCC_7521_WP_009457006.1 QNVISAAY------------------RQIFERDIAPYVASSREFKVLESKLGNGEITVKE 784

ApcE2_Synechococcus_sp_PCC_7335 STEPATSKSSVALADPPASDEPAASEDSAISENIEPSMAVALQETS-----SD------- 783

ApcE2_Chroococcidiopsis_thermalis_PCC_7203_WP_015153116.1 SLEESYEYS---QANDSER----------------------------------------- 780

ApcE2_Calothrix_sp_PCC_7507_WP_015126587.1 ---STL-------SAPNA------------------------------------------ 739

ApcE2_Chlorogloeopsis_sp_PCC_9212_WP_016873423.1 KLEPELTYV---QAAPVIE---SESEDSYA----NPA----------------------- 757

ApcE2_Fischerella_thermalis_PCC_7521_WP_009453699.1 KLEAELT--------PVIE---SESQESYI----NPV----------------------- 752

ApcE1_Synechococcus_sp_PCC_7335_WP_006453887.1 FIEGLGNSQLYIKEFYAPYPNTKVIELGTKHFLGRAPQDQGEIRKYNRILASEGIRGFIR 831

ApcE_Synechococcus_7002_WP_012307618.1 FIEGLGCSELYQKEFYTPYPNTKVIEMGTKHFLGRAPLDQQEIRKYNQILASQGLKAFIG 828

ApcE_Synechocystis_6803_WP_010873271.1 FIEGLGTSELYMKEFYAPYPNTKVIEMGTKHFLGRAPLNQKEIQQYNQILASQGLKAFIG 836

ApcE1_Calothrix_sp_PCC_7507_WP_015126818.1 FIAGLGYSNLYRKEFYTPYPNTKVIEQGTKHFLGRAPIDQAEIRKYNQILATQGINAFIG 842

ApcE1_Chroococcidiopsis_thermalis_PCC_7203_WP_015156255.1 FIEGLGNSNLYLKEFYAPYPNTKVIEQGTKHFLGRAPIDQAEIRKYNQILATQGLRAFVG 845

ApcE1_Chlorogloeopsis_sp_PCC_9212_WP_016874650.1 FIEGLGCSGLYLKEFYAPYPNTKVIELGTKHFLGRAPADQAEIRKYNQILATQGIRAFIR 846

ApcE1_Fischerella_thermalis_PCC_7521_WP_009457006.1 FIEGLGCSGLYLKEFYTPYPNTKVIELGTKHFLGRAPLDQAEIRKYNQILATQGIRAFIR 844

ApcE2_Synechococcus_sp_PCC_7335 ------------------------------------------------------------ 783

ApcE2_Chroococcidiopsis_thermalis_PCC_7203_WP_015153116.1 ------------------------------------------------------------ 780

ApcE2_Calothrix_sp_PCC_7507_WP_015126587.1 ------------------------------------------------------------ 739

ApcE2_Chlorogloeopsis_sp_PCC_9212_WP_016873423.1 ------------------------------------------------------------ 757

ApcE2_Fischerella_thermalis_PCC_7521_WP_009453699.1 ------------------------------------------------------------ 752

ApcE1_Synechococcus_sp_PCC_7335_WP_006453887.1 ALVDTPEYAQYFGEDTVPYRRFPTLPAANFPNTEILYNRLTKQSDDVVVPSFDALNPSTS 891

ApcE_Synechococcus_7002_WP_012307618.1 AMVNSMEYLDNFGEDTVPFRRFPTLPAANFPNTERLYNQLTKQNRDLVVPSFEPAVKR-- 886

ApcE_Synechocystis_6803_WP_010873271.1 AMVNGMEYLQTFGEDTVPYRRFPTLPAANFPNTERLYNKLTKQDKELVVPSFTPVVKVGG 896

ApcE1_Calothrix_sp_PCC_7507_WP_015126818.1 ALLSSAEYREVFGEDTVPYRRFPTLPAANFPNTEKLHNQLTKQNDDVVVPSFKPVKARIE 902

ApcE1_Chroococcidiopsis_thermalis_PCC_7203_WP_015156255.1 AMVNSAEYAQVFGEYYVPYRRFPTLPAANFPNTEKLYNQLTKQNDDIVVPSFEPVQPRMA 905

ApcE1_Chlorogloeopsis_sp_PCC_9212_WP_016874650.1 AMLNTPEYLEAFGEDTVPYNRFTTLPAANFPNSQKLYNQLTKQSKDIVIPSFEPTKSRMN 906

ApcE1_Fischerella_thermalis_PCC_7521_WP_009457006.1 AMLNTPEYLQAFGEDTVPYNRFTTLPAANFPNTQKLYNQLTKQSKDIVVPSFESTKPRMD 904

ApcE2_Synechococcus_sp_PCC_7335 ------------------------------------------------------------ 783

ApcE2_Chroococcidiopsis_thermalis_PCC_7203_WP_015153116.1 ------------------------------------------------------------ 780

ApcE2_Calothrix_sp_PCC_7507_WP_015126587.1 ------------------------------------------------------------ 739

ApcE2_Chlorogloeopsis_sp_PCC_9212_WP_016873423.1 ------------------------------------------------------------ 757

ApcE2_Fischerella_thermalis_PCC_7521_WP_009453699.1 ------------------------------------------------------------ 752

ApcE1_Synechococcus_sp_PCC_7335_WP_006453887.1 VSEKLPLMAGALED---------------------------------------------- 905

ApcE_Synechococcus_7002_WP_012307618.1 ------------------------------------------------------------ 886

ApcE_Synechocystis_6803_WP_010873271.1 ------------------------------------------------------------ 896

ApcE1_Calothrix_sp_PCC_7507_WP_015126818.1 -SAKTPLLAQAIADLASLAKAPDNTKPRFIELGRSFNDGRGQSVEVGVGTSRRKPARIYR 961

ApcE1_Chroococcidiopsis_thermalis_PCC_7203_WP_015156255.1 -TENMPLMAKAIADMAAKARQVDKSKPLFIELGRSYNDGRGQSVEVGVGTTRRKPARIYR 964

ApcE1_Chlorogloeopsis_sp_PCC_9212_WP_016874650.1 -VAQMPIMAKAIADMAAQARAIDKSKPLFIELGRSFNDGRGQSVEVGVGTTRRKPARIYR 965

ApcE1_Fischerella_thermalis_PCC_7521_WP_009457006.1 -AAQMPIMAKAIADMAAKARQIDKSKPLFIELGRSYNDGRGQSVEVGVGTNRRKPARIYR 963

ApcE2_Synechococcus_sp_PCC_7335 ------------------------------------------------------------ 783

ApcE2_Chroococcidiopsis_thermalis_PCC_7203_WP_015153116.1 ------------------------------------------------------------ 780

ApcE2_Calothrix_sp_PCC_7507_WP_015126587.1 ------------------------------------------------------------ 739

ApcE2_Chlorogloeopsis_sp_PCC_9212_WP_016873423.1 ------------------------------------------------------------ 757

ApcE2_Fischerella_thermalis_PCC_7521_WP_009453699.1 ------------------------------------------------------------ 752

ApcE1_Synechococcus_sp_PCC_7335_WP_006453887.1 ------------------------------------------------------------ 905

ApcE_Synechococcus_7002_WP_012307618.1 ------------------------------------------------------------ 886

ApcE_Synechocystis_6803_WP_010873271.1 ------------------------------------------------------------ 896

ApcE1_Calothrix_sp_PCC_7507_WP_015126818.1 LTNGTGQAERQLVINAAYRQVLDVFSGQVPDYYRRTELDSKLRNGEISVREFVRELASSE 1021

ApcE1_Chroococcidiopsis_thermalis_PCC_7203_WP_015156255.1 MTQGANQGEIAQVINAIYCQVMDVFSGQVPVYFRRSDLESKLRNGEISVREFIRALASSE 1024

ApcE1_Chlorogloeopsis_sp_PCC_9212_WP_016874650.1 MTVGANQAEMQQVMNAIYVQVMDVFSGQIPQYFRRSDLESKLRNGEISVREFVRDLASSE 1025

ApcE1_Fischerella_thermalis_PCC_7521_WP_009457006.1 MTVGANQAEMQQVMNAIYVQVMDVFSGQVPEYFRRSDLESKLRNGEISVREFVRDLASSE 1023

ApcE2_Synechococcus_sp_PCC_7335 ------------------------------------------------------------ 783

ApcE2_Chroococcidiopsis_thermalis_PCC_7203_WP_015153116.1 ------------------------------------------------------------ 780

ApcE2_Calothrix_sp_PCC_7507_WP_015126587.1 ------------------------------------------------------------ 739

ApcE2_Chlorogloeopsis_sp_PCC_9212_WP_016873423.1 ------------------------------------------------------------ 757

ApcE2_Fischerella_thermalis_PCC_7521_WP_009453699.1 ------------------------------------------------------------ 752

ApcE1_Synechococcus_sp_PCC_7335_WP_006453887.1 ------------------------------------------------------------ 905

ApcE_Synechococcus_7002_WP_012307618.1 ------------------------------------------------------------ 886

ApcE_Synechocystis_6803_WP_010873271.1 ------------------------------------------------------------ 896

ApcE1_Calothrix_sp_PCC_7507_WP_015126818.1 IYRKRFYTPYPNTKVIEYLFRHLLGRAPATQGEIRTYNKLLADSGLRAAVEGIIDSPEYA 1081

ApcE1_Chroococcidiopsis_thermalis_PCC_7203_WP_015156255.1 IYCRRFYTPYPNTKVIEFLFRHLLGRAPATQGEIRQYNKLLSEGGLKAAVDAMVESPEYA 1084

ApcE1_Chlorogloeopsis_sp_PCC_9212_WP_016874650.1 IYRKRFYTPYPNTKVIEFLFRHLLGRAPATQAEIRQYNKLLADGGLKAAVEAMVNSPEYA 1085

ApcE1_Fischerella_thermalis_PCC_7521_WP_009457006.1 IYRKRFYTPYPNTKVIEFLFRHLLGRAPATQAEIRQYNKLLADSGLRAAVEAMVNSPEYA 1083

ApcE2_Synechococcus_sp_PCC_7335 ------------------------------------------------------------ 783

ApcE2_Chroococcidiopsis_thermalis_PCC_7203_WP_015153116.1 ------------------------------------------------------------ 780

ApcE2_Calothrix_sp_PCC_7507_WP_015126587.1 ------------------------------------------------------------ 739

ApcE2_Chlorogloeopsis_sp_PCC_9212_WP_016873423.1 ------------------------------------------------------------ 757

ApcE2_Fischerella_thermalis_PCC_7521_WP_009453699.1 ------------------------------------------------------------ 752

ApcE1_Synechococcus_sp_PCC_7335_WP_006453887.1 ---------------------------------------------------- 905

ApcE_Synechococcus_7002_WP_012307618.1 ---------------------------------------------------- 886

ApcE_Synechocystis_6803_WP_010873271.1 ---------------------------------------------------- 896

ApcE1_Calothrix_sp_PCC_7507_WP_015126818.1 RYFGEDVVPYQRFPSLPAGNYLGSVQVAADLVKQSWSSLSPAVLTGRSDR-- 1131

ApcE1_Chroococcidiopsis_thermalis_PCC_7203_WP_015156255.1 QYFGEDVVPYRRYPSLPAGNYLGSVKAAADLVKQSWSDLSPSLLAQSFGQR- 1135

ApcE1_Chlorogloeopsis_sp_PCC_9212_WP_016874650.1 RYFGEDVVPYQRYPSLPAGNYLGSVKAAADLVKQSWSSLSPSVLTGRYSQG- 1136

ApcE1_Fischerella_thermalis_PCC_7521_WP_009457006.1 RYFGEDVVPYQRFPSLPAGNYLGSVKAAADLVKQSWSSLSPSVLTGRYTQGG 1135

ApcE2_Synechococcus_sp_PCC_7335 ---------------------------------------------------- 783

ApcE2_Chroococcidiopsis_thermalis_PCC_7203_WP_015153116.1 ---------------------------------------------------- 780

ApcE2_Calothrix_sp_PCC_7507_WP_015126587.1 ---------------------------------------------------- 739

ApcE2_Chlorogloeopsis_sp_PCC_9212_WP_016873423.1 ---------------------------------------------------- 757

ApcE2_Fischerella_thermalis_PCC_7521_WP_009453699.1 ---------------------------------------------------- 752

**C**

β-subunits (B1, B2, and B3)

ApcB1 Thermosynechococcus vestitus BP-1 (WP_011056800.1) -MQDAITAVINASDVQGKYLDTAAMEKLKAYFATGELRVRAASVISANAANIVKEAVAKS 59

ApcB1 Calothrix sp. PCC 7507 (WP_015126816.1) MAQDAITAVINSADVQGKYLDTAAIEKLKGYFSSGDLRVRAAGTISANAAVIVKEAVAKS 60

ApcB1 Chroococcidiopsis thermalis PCC 7203 (WP_015156257.1) -MQDAITSVINTSDVQGKYLDTAAMEKLKGYFQSGELRVRAATTIAANAAAIVKEAVAKS 59

ApcB1 Anabaena sp. PCC 7120 (WP_010994199.1) MAQDAITAVINSADVQGKYLDTAALEKLKAYFSTGELRVRAATTISANAAAIVKEAVAKS 60

ApcB1 Chlorogloeopsis sp. PCC 9212 (WP_016874648.1) -MQDAITAVINSSDVQGKYLDTAALEKLKGYFSTGELRVRAATTIAANAAAIVKEAVAKS 59

ApcB1 Fischerella thermalis PCC 7521 (WP_009457009.1) -MQDAITAVINSSDVQGKYLDTAALEKLKSYFSTGELRVRAATTIAANAAAIVKEAVAKS 59

ApcB1 Synechococcus sp. PCC 6301 (WP_011243499.1) -MQDAITAVINASDVQGKYLDSSALDRLKSYFQSGELRVRAAATISANSALIVKEAVAKS 59

ApcB1 Synechococcus sp. PCC 7002 (WP_012307539.1) -MQDAITSVINSADVQGKYLDGSAMDKLKAYFTTGALRVRAASTISANAAAIVKEAVAKS 59

ApcB1 Synechococcus sp. PCC 7335 (WP_006456852.1) -MQDAITAVINASDVQGKYLDSSSMDKLKAYFQTGELRVRAATSISANAAEIVKEAVAKS 59

ApcB1 Synechocystis sp. PCC 6803 (WP_010872504.1) -MQDAITAVINSADVQGKYLDGAAMDKLKSYFASGELRVRAASVISANAATIVKEAVAKS 59

ApcB1 Leptolyngbya sp. JSC-1 (MBF2047188.1) -MQDAITSVINSADVQGKYLDSTAMDKLKSYFSSGELRVRAATAISANAAAIVKEAVAKS 59

ApcB2 Synechococcus sp. PCC 7335 (WP_006454442.1) -MQDAITTLINTSDAQGKYLDDSSLDTLQEYFRSGDLRAKAAMTISANASTIVTKTVAKS 59

ApcB2 Leptolyngbya sp. JSC-1 (WP_036011375.1) -MQDAITALINSSDVQGRYLDNNGLDKLRSYFQSGEMRARAAITISANASSLVTQAVAKS 59

ApcB2 Chlorogloeopsis sp. PCC_9212 (WP_016873421.1) -MQDAITSLINSSDVQGKYLDNNSLDKLQHYYHTGDMRARAATAISANAKTIVTQTVAKS 59

ApcB2 Fischerella thermalis PCC 7521 (WP_009453701.1) -MQDAITSLINSSDVQGKYLDNNSLEKLQHYYHTGDMRARAATTISANAKTIVTQTVAKS 59

ApcB2 Chroococcidiopsis thermalis PCC 7203 (WP_015153114.1) -MQDAITALINSSDVQGRYLDPSSLDKLQNYFQSGDMRAKTAIAVSANAKNIVTKTVAKS 59

ApcB2 Calothrix sp. PCC 7507 (WP_015126589.1) -MQDAITALINSSDVQGKYLDSSSLEKLQNYFHSGDVRARAATTVSANAKNIVTKAVAKS 59

ApcB3 Synechococcus sp. A1463 (WP_099812041.1) -MKDTITSLINPADEKGSYLDAAALEQLNRYFQSGNMRVKAAKTISSSASSIISKTVAKS 59

ApcB3 Chlorogloeopsis sp. PCC 9212 (WP_016874154.1) -MQDTITSLINPADLRGKYLDNTELDKLRKYFQSGELRVKAAATISENAANIVSQAVANS 59

ApcB3 Chroococcidiopsis thermalis PCC 7203 (WP_015156292.1) -MQDTITSVINPADRQGKYLDTPELEKLRKYFQTGELRVKAAATISENASSIVSQAVANS 59

ApcB3 Synechococcus sp. PCC 7335 (WP_006453385.1) -MQDTITSLINPADEKGQYLEGGDLDSLKQYLQSGATRVKAAGQIGDSAASIISKTVERS 59

ApcB3 Gloeocapsa sp. PCC 7428 (WP_015190165.1) -MQDTITSLINPADQRGKYLETEELEKLRRYFQSGELRVKAASAISNNAANIIREAVANS 59

ApcB3 Gloeomargarita lithophora D10 (WP_071455396.1) -MQDTITSLINPADEQGQYLNAAALDQLNKYFQKGAVRVQAASTISDTASSIISKTVAKS 59

ApcB3 Halomicronema hongdechloris (WP_080809690.1) -MQDIITAAINPADERCAYLEDSSLEKLRQYYQSGTLRLKAATQIGNSAASIISDAVRKS 59

ApcB3 Xenococcus sp. PCC 7305 (WP_006512042.1) -MQDTITSLINPADEKGQYLEGQELDQLKKYFQSGSLRVKAADQIGSAAASIITESVAKS 59

ApcB3 Leptolyngbya sp. PCC 6406 (WP_008312401.1) -MQDTITATINPADEQGIYLEGEQLDALKAYFQSGTLRVKAASQIGDSAASIISETVAKS 59

:* **: ** :* : **: :: *. * .* * ::* :. : :: .:* .*

|||

ApcB1 Thermosynechococcus vestitus BP-1 (WP_011056800.1) LLYSDITRPGGNMYTTRRYAACIRDLDYYLRYATYAMLAGDPSILDERVLNGLKETYNSL 119

ApcB1 Calothrix sp. PCC 7507 (WP_015126816.1) LLYSDITRPGGNMYTTRRYAACIRDLDYYLRYATYAMLAGDASILDERVLNGLKETYNSL 120

ApcB1 Chroococcidiopsis thermalis PCC 7203 (WP_015156257.1) LLYSDITRPGGNMYTTRRYAACIRDLDYYLRYSTYAMLAGDPSILDERVLNGLKETYNSL 119

ApcB1 Anabaena sp. PCC 7120 (WP_010994199.1) LLYSDITRPGGNMYTTRRYAACIRDLDYYLRYATYAMLAGDPSILDERVLNGLKETYNSL 120

ApcB1 Chlorogloeopsis sp. PCC 9212 (WP_016874648.1) LLYSDITRPGGNMYTTRRYAACIRDLDYYLRYATYAMLAGDPSILDERVLNGLKETYNSL 119

ApcB1 Fischerella thermalis PCC 7521 (WP_009457009.1) LLYSDITRPGGNMYTTRRYAACIRDLDYYLRYATYAMLAGDPSILDERVLNGLKETYNSL 119

ApcB1 Synechococcus sp. PCC 6301 (WP_011243499.1) LLYSDITRPGGNMYTTRRYAACIRDLEYYLRYATYAMLAGDTSILDERVLNGLKETYNSL 119

ApcB1 Synechococcus sp. PCC 7002 (WP_012307539.1) LLYSDVTRPGGNMYTTRRYAACIRDLDYYLRYATYAMLAGDPSILDERVLNGLKETYNSL 119

ApcB1 Synechococcus sp. PCC 7335 (WP_006456852.1) LLYSDITRPGGNMYTTRRYAACIRDLDYYLRYSTYAMLAGDPSILDERVLNGLKETYNSL 119

ApcB1 Synechocystis sp. PCC 6803 (WP_010872504.1) LLYSDVTRPGGNMYTTRRYAACIRDLDYYLRYATYAMLAGDASILDERVLNGLKETYNSL 119

ApcB1 Leptolyngbya sp. JSC-1 (MBF2047188.1) LLYSDITRPGGNMYTTRRYAACIRDLDYYLRYATYAMLAGDPSILDERVLNGLKETYNSL 119

ApcB2 Synechococcus sp. PCC 7335 (WP_006454442.1) LLYTDITGPGGNMYTCRRYAACIRDMDFFLRYGTYAMLAGDASILDERVLNGLKETYNSL 119

ApcB2 Leptolyngbya sp. JSC-1 (WP_036011375.1) LMYTDITAPGGNMYTCRRYAACIRDLDYFLRYATYAMLAGDPSILDERILNGLRETYNSL 119

ApcB2 Chlorogloeopsis sp. PCC_9212 (WP_016873421.1) LLYTDITAPGGNMYTCRRYAACVRDLDYFLRYATYAMLAGDPSILDERILNGLRETYNSL 119

ApcB2 Fischerella thermalis PCC 7521 (WP_009453701.1) LLYTDITAPGGNMYTCRRYAACVRDLDYFLRYATYAMLAGDPSILDERILNGLRETYNSL 119

ApcB2 Chroococcidiopsis thermalis PCC 7203 (WP_015153114.1) LLYTDITAPGGNMYTCRRYAACVRDLDYFLRYATYAMLAGDTSILDERILNGLRETYNSL 119

ApcB2 Calothrix sp. PCC 7507 (WP_015126589.1) LLYTDITGPGGNMYTCRRYAACIRDLDYFLRYATYAMLAGDPSILDERILNGLRETYNSL 119

ApcB3 Synechococcus sp. A1463 (WP_099812041.1) LLYGDITLPGGNMYPTRRYAACLRDLTYFLRYATYAMLAADPSILDERVLQGLKETYITL 119

ApcB3 Chlorogloeopsis sp. PCC 9212 (WP_016874154.1) LLYGDITCPGGNMYPTRRYAACLRDLTLFLRYATYAMLADDASVLDERVLDGLKETYNSL 119

ApcB3 Chroococcidiopsis thermalis PCC 7203 (WP_015156292.1) LLYGDITCPGGNMYPTRRYAACIRDLTLFLRYATYAMLADDPSIIEERVLFGLKETFSTL 119

ApcB3 Synechococcus sp. PCC 7335 (WP_006453385.1) LLYGDITLPGGNMYPTRRYAACLQDLTYFLRYATYAMLADDASIIDERILNGLKDTYSSL 119

ApcB3 Gloeocapsa sp. PCC 7428 (WP_015190165.1) LLYGDITCPGGNMYPTRRYAACIRDLTLFLRYATYAMLAADPSILDERVLDGLKETYNSL 119

ApcB3 Gloeomargarita lithophora D10 (WP_071455396.1) LLYGDITLPGGNMYPTRRYAACLRDLNYFLRYATYAMLAADASILDERVLNGLKETYAAL 119

ApcB3 Halomicronema hongdechloris (WP_080809690.1) LLYGTITEPGGNMYPWRRYAACLRDLNYFLRYATYAMLAADASIIDERVLNGLRETYLSL 119

ApcB3 Xenococcus sp. PCC 7305 (WP_006512042.1) LLYGDITLPGGNMYPTRRYAACLRDLTYFLRYAVYAMLADDPSILDERVLNGLKDTYLSL 119

ApcB3 Leptolyngbya sp. PCC 6406 (WP_008312401.1) LLYGDITCPGGNMYPTRRYAACLRDLTYFLRYATYAMLAADASILDERVLNGLKETYSSL 119

*:* :* ****** ******::*: :***..***** * *:::**:* **::*: :*

ApcB1 Thermosynechococcus vestitus BP-1 (WP_011056800.1) GVPIAATVQAIQAMKEVTASLVGADAGKEMGIYFDYICSGLS- 161

ApcB1 Calothrix sp. PCC 7507 (WP_015126816.1) GVPVGATVQAIQAIKEVTAGLVGSDAGREIGVYLDYISSGLS- 162

ApcB1 Chroococcidiopsis thermalis PCC 7203 (WP_015156257.1) GVPVGATVQAIQAMKEVTASLTGPDAGKEMGVYFDYICSGLS- 161

ApcB1 Anabaena sp. PCC 7120 (WP_010994199.1) GVPVGATVQAIQAIKEVTASLVGADAGKEMGIYLDYISSGLS- 162

ApcB1 Chlorogloeopsis sp. PCC 9212 (WP_016874648.1) GVPIGATVQAIQAMKEVTASLVGPDAGKEMGVYFDYISSGLS- 161

ApcB1 Fischerella thermalis PCC 7521 (WP_009457009.1) GVPIGATVQAIQAMKEVTASLVGPDAGKEMGVYLDYICSGLS- 161

ApcB1 Synechococcus sp. PCC 6301 (WP_011243499.1) GVPIGATVQAIQAIKEVTASLVGPDAGREMGVYLDYISSGLS- 161

ApcB1 Synechococcus sp. PCC 7002 (WP_012307539.1) GVPVGSTVQAIQAMKEVTAGLVGADAGREMGVYFDYICSGLS- 161

ApcB1 Synechococcus sp. PCC 7335 (WP_006456852.1) GVPVGATVQAIQAIKEVTASLVGADAGKEMGVYLDYICSGLS- 161

ApcB1 Synechocystis sp. PCC 6803 (WP_010872504.1) GVPISSTVQAIQAIKEVTASLVGADAGKEMGVYLDYICSGLS- 161

ApcB1 Leptolyngbya sp. JSC-1 (MBF2047188.1) GVPIAATVQAIQAIKEVTASLVGADAGKEMGVYLDYICSGLS- 161

ApcB2 Synechococcus sp. PCC 7335 (WP_006454442.1) GVPVGATIRAVQAMKEVVNDMLGAEAGKEVGYYFDHICSGLS- 161

ApcB2 Leptolyngbya sp. JSC-1 (WP_036011375.1) GVPIGATIRSVQAMKEATTDLVGAEAGKEMGVYFDYICAGLS- 161

ApcB2 Chlorogloeopsis sp. PCC_9212 (WP_016873421.1) GVPIGATIRAVQAMKEVTNSIVGAEAGKEMGVYFDYIASGLS- 161

ApcB2 Fischerella thermalis PCC 7521 (WP_009453701.1) GVPIGATIRAVQAMKEVTNSMIGADAGKEMGVYFDYIASGLS- 161

ApcB2 Chroococcidiopsis thermalis PCC 7203 (WP_015153114.1) GVPIGATIRSVQAMKEVVTSLVGADAGREMGVYFDHIAAGLS- 161

ApcB2 Calothrix sp. PCC 7507 (WP_015126589.1) GVPVGATIRSVQALKEVSTSIVGADAGKELGVYFDYIASGLS- 161

ApcB3 Synechococcus sp. A1463 (WP_099812041.1) GVPIDRVIQALNAMKEVLTESLDTEASQEMAVYLDHIIAGLS- 161

ApcB3 Chlorogloeopsis sp. PCC 9212 (WP_016874154.1) GVPVDRTIQAVQAMKEVITRQVGAEAGEQVGRHLDHICNGLS- 161

ApcB3 Chroococcidiopsis thermalis PCC 7203 (WP_015156292.1) GVPIQPTVQAIQALKEVTTRLVGAEAGQEVGTHLDHICSGLSQ 162

ApcB3 Synechococcus sp. PCC 7335 (WP_006453385.1) GVPVEPTIQAIEAMKDVVSERVGTEAGQEVGKYLDHIIAGLR- 161

ApcB3 Gloeocapsa sp. PCC 7428 (WP_015190165.1) GVPIQPTIQAIQAMKEVTTRLVGAEAGGEIGMYFDHICNGLS- 161

ApcB3 Gloeomargarita lithophora D10 (WP_071455396.1) GVPIDRVVEALNAMKEVLHGAVGAEAGQELGVYLDHITAGLA- 161

ApcB3 Halomicronema hongdechloris (WP_080809690.1) GVPIEPTIQAIQAMKEVVTQRVGADAGQEMDVYLDHIISGLS- 161

ApcB3 Xenococcus sp. PCC 7305 (WP_006512042.1) GVPIEPTIQAVQAMKEVVTQRVGAEAGQEMDVYLDHIIAGLG- 161

ApcB3 Leptolyngbya sp. PCC 6406 (WP_008312401.1) GVPVEATIQAVQAMKEVVTHRVGADAGQEMDVYLDHIIAGLS- 161

***: .:.:::*:*:. . :*. :: ::*:* **

**Figure S15.** **Multiple sequence alignments of α-subunits and β-subunits for representatives of the AP family.** *A*, sequence alignments for α-subunits (excluding ApcE). *B*, sequence alignments for ApcE subunits. *C*, sequence alignments for β-subunits (excluding ApcF subunits). The organism name for sequences corresponding to the structure determined herein are highlighted. Those sequences are colored according to the subunit colors in **Fig. 1**. Below the alignments, the Clustal Omega sequence conservation identifiers are shown. For *A* (α-subunit sequences), the approximate sequence region corresponding to the BE loop of the α-subunit that defines the environment of its phycocyanobilin ring A is denoted with vertical lines above the multiple sequence alignment. For *B* (ApcE sequences), the approximate region of the sequence that interacts with pyrrole ring D of β-subunit chromophores in the FaRLiP-AP core structure is designated by horizontal lines, and the Cys residues form thioether linkages to phycocyanobilin chromophores are highlighted in magenta. For *C* (β-subunit sequences), the approximate sequence region corresponding to the BE loop of the β-subunit that defines the environment of the adjacent α-subunits phycocyanobilin ring D is denoted with vertical lines above the multiple sequence alignment.

ApcA1_4RMP --------VKADAEARYLSPGELDRIKGFVTSGERRLRIAQVLTESRECIVKQAGDQLFQ 52

ApcD1_4PO5 MSVVSQVILQADDQLRYPTSGELKGIQAFLTTGAQRIRIAETLAENEKKIVDQAQKQLFK 60

ApcD2_FaRLiP MSVISQVIATADREVRYLSKGELDAINRFFNNGPQRLRIVSILNSNAEEIVEKGARRFWQ 60

ApcD3_FaRLiP MSIVKQIISNADEELRYPTPGELEMIRSFCKTGASQIQLAKTLESHAPTIVERGTRKFWQ 60

ApcD4_LoLiP MSIVAQVIAQSDAADRFLSSAEIAKLEDFFSKGQVRIRAAQKLAENEQKIVQEGSKRFWA 60

ApcD5_FaRLiP MSLVTELILSADSEARYPAPKELRIFQDFVKTGEQRVRIAKALAANEERIVQNGSQKFWE 60

:* *: : *: :. * ..* ::: .. * **... :::

|||||||||||||||||

ApcA1_4RMP KRPDVVSPGGNAYGEEMTATCLRDMDYYLRLITYGVVAGDVTPIEEIGLVGVREMYNSLG 112

ApcD1_4PO5 KHPEYRAPGGNAYGQRQYNQCLRDYGWYLRLVTYGVLAGNKEPIETTGLIGVKEMYNSLN 120

ApcD2_FaRLiP RCPITPSNSDN---QQFQASCLRDQAWFIRLISYAVAVGDVDPLEASGVRGVREMYLSLE 117

ApcD3_FaRLiP ICPRTPSNSGS---PRKTEAAQRDMSWYIRLISYCLLAGNDQPLREIGLLGMKELYTNIG 117

ApcD4_8DDY KCPNTPSNKGN---PQKTALCQRDQGWYIRLVSYCILAGNDKPLEDIGLNGMREMYISLG 117

ApcD5_FaRLiP RCPNTPSNSGV---DRKTASCQRDQGWYVRLIAYSILAGSERPLEDIGTVGIKEMYNNLE 117

* : . . . ** :::**::* : .*. *:. * *::*:* .:

ApcA1_4RMP TPIPAVAEAVRCMKSVASSLLSGENAAEAASYFDYVVGAMQ------------------- 153

ApcD1_4PO5 VPVPGMVDAVTVLKDAALGLLSAEDANETAPYFDYIIQFMS------------------- 161

ApcD2_FaRLiP VPLRSVALCMRSLKEVTLAMLSREDAAEVGPYFDYLIAGLMP------------------ 159

ApcD3_FaRLiP IPLDNILQYLRCLKAEAIALLSEAEAEAIIPYFDQIIQELVRPGPSYFGIKDRSARQSAR 177

ApcD4_8DDY VPLPNLRVAMSCLKEVAAGILSSEEMALAAPYFDRLIRAF-------------------- 157

ApcD5_FaRLiP IPIRNIAECMRCLKEEAMAVLSDEDAQEVAAYFDLIIQSLS------------------- 158

*: : : :* : .:** : *** :: :

ApcA1_4RMP ---- 153

ApcD1_4PO5 ---- 161

ApcD2_FaRLiP ---- 159

ApcD3_FaRLiP QAAA 181

ApcD4_8DDY ---- 157

ApcD5_FaRLiP ---- 158

**Figure S16.** **Multiple sequence alignment of selected AP family α-subunits for which structures are available.** Residues that interact with the phycocyanobilin on the α-subunit are highlighted in yellow. The Arg sidechain that interacts with the putative Cl^–^ anion (**Figure S10**) in one of the ApcD5 subunits of the FaRLiP AP core is highlighted in green. The unmodeled C-terminal region of ApcD3 in the FaRLiP AP core is highlighted in grey. The approximate region of the BE loop is shown as vertical lines above the alignment. Below the alignments, the Clustal Omega sequence conservation identifiers are shown.

*bbbbbbbbbbbbbbbbbbbbbbbbbbbbbbbbbbbbbbbbbbbbbbbbbbbbbbbbbbbb*

ApcE2 MTDRTNGGSPVVHPQQYHTVPTAVINGAHQRDRYPNHSEMQTLSTFLRTGLQRLEIAQTL 60

ApcE1 MSVKASGGSSLARPQLYQTVPVSAISQAEQQDRFLEGSELNELTAYFQSGALRLEIAETL 60

*: ::.*** :.:** *:***.:.*. *.*:**: : **:: *::::::* *****:**

*bbbbbbbbbbbbbbbbbbbbbbbbbbbbbbbbbbbbbbbbbbbbbbbbbbbbbbbbbbbb*

ApcE2 AQHANEIVAAGGKRIFVGGNPMAYFEQPEELVGMPGSGYFVAEDYLSPKSRRQTGNGHSV 120

ApcE1 TQNADLIVSRAANRIFTGGSPLSYLEKPVERQPALVG----------ASSD--SRNGSVT 108

:*:*: **: ..:***.**.*::*:*:* * . .* : ** .

*bbbbbbbbbbbbbbbbbbbbbbbbbbbbbbbbbbbbbbbbbbbbbbbbbbbbbbbbbbbb*

ApcE2 QNSSSSITNPVAWLKGLFFSGKPSVPSRFQAINIADYGAVRMKRSMRDLGWFLRYITYAV 180

ApcE1 YAESNGSGGLFGGLR-SVFSSTGPIPPGFRPINIARYGPSNMQKSLRDMSWFLRYTTYAI 167

.*.. . .. *: .**.. :* *: **** ** .*::*:**:.***** ***:

*bbbbbbbbbbbbbbbbbbbbbbbbbbbbbbbbbbbbbbbbbbbbbbbbbbbbbbbbbbbb*

ApcE2 VAGDTSIITVNTRGLRGIIPEDVTVA-TTVALQEMQWKSLSFFPVDSAAAALVRRYFDVL 239

ApcE1 VAGDPNIIVVNTRGLKEVIENACSIDATIVAIQEMRAASADYFRNNAQAKEIVLQYFDIL 227

**** .**.******: :* : :: * **:***: * .:* :: * :* :***:*

ApcE2 IADYQVEKPSDRYRTGVSKHDQGLSFPESYEDSGCAIPRWVMKPTLPDSEKDAVIRAAYR 299

ApcE1 LSEFKAPTPANKVRQGPSNDIQGLELPQSYFNAAAKRQKYAMKPGLSALEKNAVIKAAYR 287

:::::. .*::: * * *:. ***.:*:** ::.. ::.*** * **:***:****

ApcE2 QVFERDISGLGTAELTQPISQLKGEDGSMELFIRQLGKSRLYRQLFYEPYMISRSIELAC 359

ApcE1 QIFERDITKAYSQSISYLESQVRNGDISMKEFVRRLAKSPLYRKQFFEPFINSRALELAF 347

*:*****: : .:: **::. * **: *:*:*.** ***: *:**:: **::***

ApcE2 RHFLGRGLSCMEEFQRYFELVADQGFSALVDALVSSQEYADYFGAETVPYIRGLGIEAQA 419

ApcE1 RHILGRGPSSREEVQKYFSIVSSGGLPALVDALVDSQEYADYFGEETVPYLRGLGVEAQE 407

**:**** *. **.*:**.:*:. *: *******.********* *****:****:***

ApcE2 CRNWGPQLDLFKYSAPARKVPQFVTAFASYRQPLPNQHPYGMGNDPLETQFGAIFPHETT 479

ApcE1 CRNWGMQQDLFSYSAPFRKVPQFITTFAQYDRPLPDQHVYGSGNDPLEIQFGAIFPKETR 467

***** * ***.**** ******:*:**.* :***:** ** ****** *******:**

ApcE2 NPAAQPVHFSEDSRRILVGHAHRKSHA------------EISQQIFSLKTLAHKPTKASE 527

ApcE1 NPSKRPAPFNKDTKRILIHRGPAVNNQVGNPSAVGEFPGSLGAKVFRLNGGLPGAK---V 524

**: :*. *.:*::***: :. .: .:. ::* *: .

ApcE2 SLSFFPSSDSRQHSVESVILAAYRQVFGCEVLGSQRHQAAETQLKGGLITVREFVRQLAK 587

ApcE1 GKNTGTSVKFGESSTQALIRAAYRQVFGRDLYEGQRLSVAEIQLENGDISVREFIKRLAK 584

. . * . : *.:::* ******** :: .** ..** **:.* *:****:::***

ApcE2 SRSFRQAYWENLYMTKAAEIIHRRLLGRPTYGRRETSKYYDICGRQGFYALVDALIDSDD 647

ApcE1 SELFLKLYWAPHYVCKAIEYMHRRLLGRPTYGRQEMNQYFDIASKQGFYAVVEAMIDSKE 644

*. * : ** *: ** * :************:* .:*:**..:*****:*:*:***.:

ApcE2 YRTAFGENTVPYERYVTPRGLALRSPKGPVAISKLRDNPHTVGEYMMRYQPPAANISPRS 707

ApcE1 YSDAFGEDTVPYERYLTPGGLQMRSARVGSLREDIGQRVD--KEVTPRF----VELGQVS 698

* ****:*******:** ** :** : ..: :. . * *: .::. *

ApcE2 PLNNSASNRQPATARHSDNGALEDRSASSTEPAT-SKSSVALADPPASDEPAASEDSAIS 766

ApcE1 -----AIRTEPEIAYRSNQGVTRQRQQTKVFKLVSTYDKVAVKNA-----IRAAYRQVFE 748

* . :* * :*::*. .:*. :.. . : ..**: : *: ..:.

ApcE2 ENIEPSMA----VALQETSSD--------------------------------------- 783

ApcE1 RDLEPYIINSEFTALESKLSNNEINVKEFIEGLGTSELYMKEFYAPYPNTKVIEMGTKHF 808

.::** : .**:.. *:

ApcE2 ------------------------------------------------------------ 783

ApcE1 LGRAPLNQKEIQQYNQILASQGLKAFIGAMVNGMEYLQTFGEDTVPYRRFPTLPAANFPN 868

ApcE2 ---------------------------- 783

ApcE1 TERLYNKLTKQDKELVVPSFTPVVKVGG 896

**Figure S17. Sequence alignment comparing ApcE2 from *Synechococcus* 7335 and ApcE1 from *Synechocystis* 6803.** Sequences correspond to those found in the structure presented here (NCBI Reference WP_010873271.1) and the structure of ApcE1 in the PBS structure of *Synechocystis* 6803 (PDB 7SC9, NCBI Reference WP_006455341.1). The sequence identity is 44.74%. Residues interacting with the chromophore are highlighted in yellow, and the approximate region of the sequence corresponding to the bilin-binding divergent α-subunit domain has “*b*” above the alignment. Residues in ApcE2 that interact with the putative Cl^–^ anion are highlighted in green. The Cys residue that covalently links the phycocyanobilin chromophore in ApcE1 is highlighted in magenta. The first gray shaded region comprises unmodeled residues in ApcE2 from the FaRLiP AP core structure and ApcE1 from the *Synechocystis* sp. PCC 6803 PBS structure, which likely interact with PSII *in vivo*. The second gray shaded region is unmodeled in ApcE2. In ApcE1, this region interacts with a fourth AP trimer in the cylinder. The third gray shaded region is unmodeled in ApcE2. The blue highlighted region corresponds to the third REP domain of ApcE1.


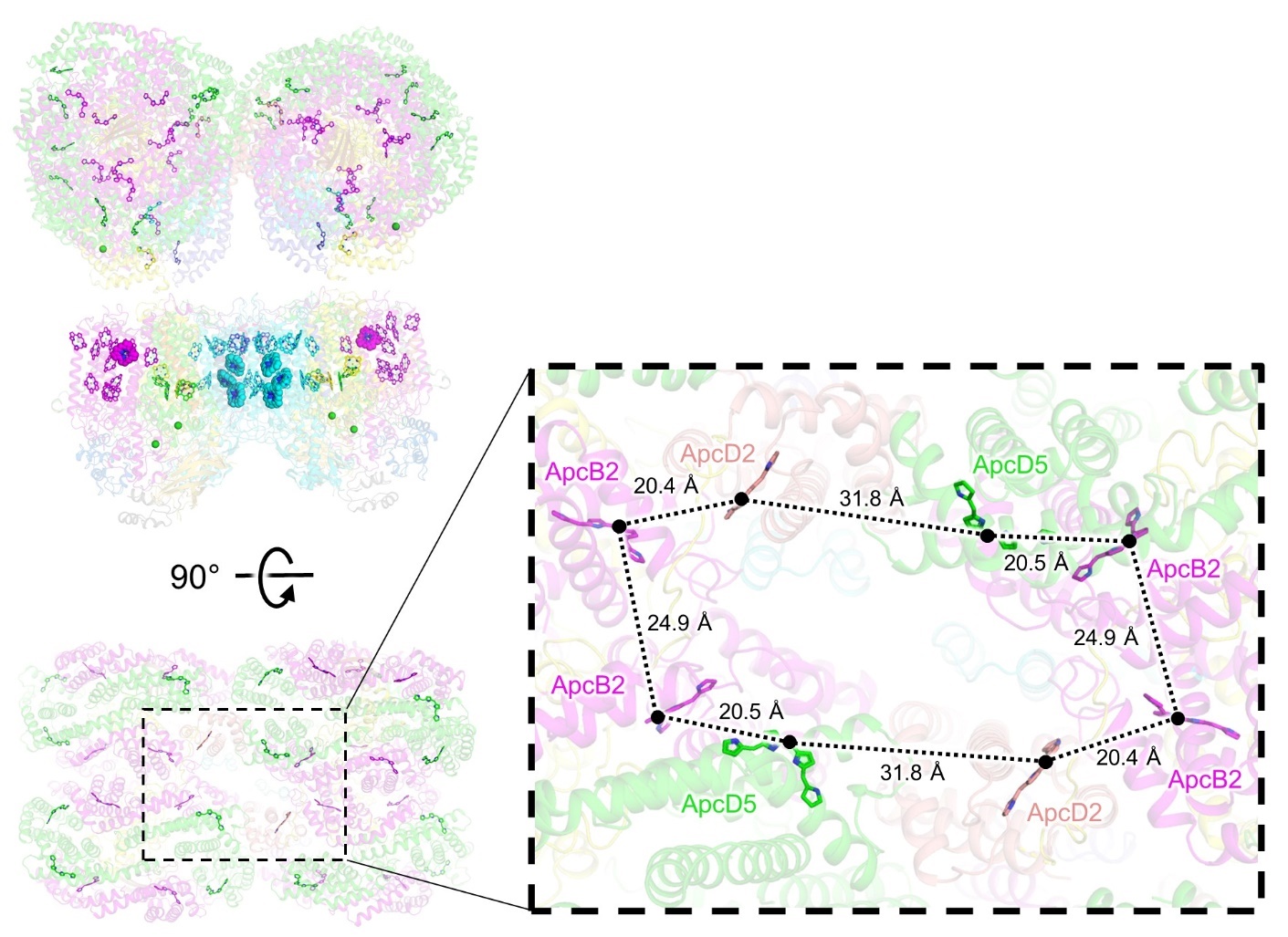


**Figure S18. Distances between chromophores near the interface of the two cylinders of the FaRLiP-AP core complex.** Colors are identical to those shown in Figure 2. Only tetrapyrrole rings are shown for clarity. In the inset, center-to-center distances are shown.


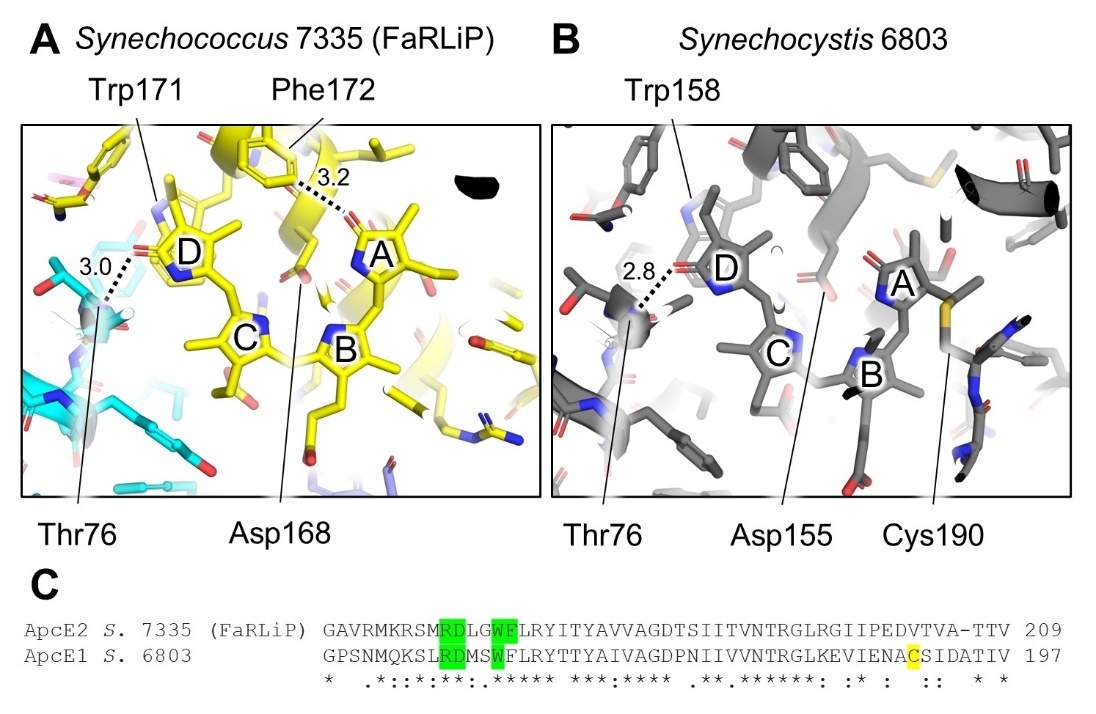


**Figure S19. Comparison of phycocyanobilin chromophores bound to ApcE2 and ApcE1.** *A*, chromophore and its environment of ApcE2. *B*, chromophore and its environment of ApcE1 (PDB 7SC9). *C*, partial sequence alignment of residues near the ApcE2/E1 chromophores. The Cys190 residue that provides a thioether linkage to the ApcE1 phycocyanobilin is highlighted in yellow. There is no covalent linkage to the ApcE2 phycocyanobilin. Other residues that interact with the phycocyanobilin are highlighted in green. These sequences are the same as those shown in **Figure S15**.

ApcB1 MQDAITAVINSADVQGKYLDGAAMDKLKSYFASGELRVRAASVISANAATIVKEAVAKSL 60

ApcB2 MQDAITTLINTSDAQGKYLDDSSLDTLQEYFRSGDLRAKAAMTISANASTIVTKTVAKSL 60

ApcB3 MKDTITSLINPADEKGSYLDAAALEQLNRYFQSGNMRVKAAKTISSSASSIISKTVAKSL 60

ApcF MRDTVTSLISNYDTTGRYLDRDAIDSLQSYFITGANRVKVAAMISANAAEISREAGKKLF 60

*:*::*::*. * * *** ::: *: ** :* *.:.* **:.*: * :: * :

ApcB1 -LYSDVTRPGGNMYTTRRYAACIRDLDYYLRYATYAMLAGDASILDERVLNGLKETYNSL 119

ApcB2 -LYTDITGPGGNMYTCRRYAACIRDMDFFLRYGTYAMLAGDASILDERVLNGLKETYNSL 119

ApcB3 -LYGDITLPGGNMYPTRRYAACLRDLTYFLRYATYAMLAADPSILDERVLQGLKETYITL 119

ApcF EVVPELIRPGGNAYTTRRYAACLRDMDYYLRYSSYALVAGNNDVLMERVLQGLRETYNSL 120

: :: **** * ******:**: ::***.:**::*.: .:* ****:**:*** :*

ApcB1 GVPISSTVQAIQAIKEVTASLVG---ADAGKEMGVYLDYICSGLS---- 161

ApcB2 GVPVGATIRAVQAMKEVVNDMLG---AEAGKEVGYYFDHICSGLS---- 161

ApcB3 GVPIDRVIQALNAMKEVLTESLD---TEASQEMAVYLDHIIAGLS---- 161

ApcF GVPIAPTVQGIQIMKEMVKERASDMGVDDTSFIDQPFDFISREVSEISV 169

***: .::.:: :**: . . .: . : :*.* :*

**Figure S20. Sequence alignment of selected AP family β-subunits for which structures are available.** Residues that interact with the phycocyanobilin on the β-subunit are highlighted in yellow. The yellow highlighted Asn (N) residues are methylated on the amido nitrogen of the sidechain, the keto-oxygen interacting with the chromophore. The red highlighted Asn residue is also methylated, but its sidechain does not interact with the chromophore. Instead, its backbone carbonyl oxygen interacts with the chromophore. The ApcB1 sequence (NCBI Reference WP_010872504.1) is the one from the structure of AP-B (PDB 4PO5). The ApcB2 sequence is the (NCBI Reference WP_006454442.1) is the one from the structure solved herein. The ApcB3 sequence (NCBI Reference WP_099812041.1) is the one from the structure of helical FRL-AP (PDB 8DDY). The ApcF sequence (NCBI Reference WP_065714071.1) is the one from the structure solved herein.


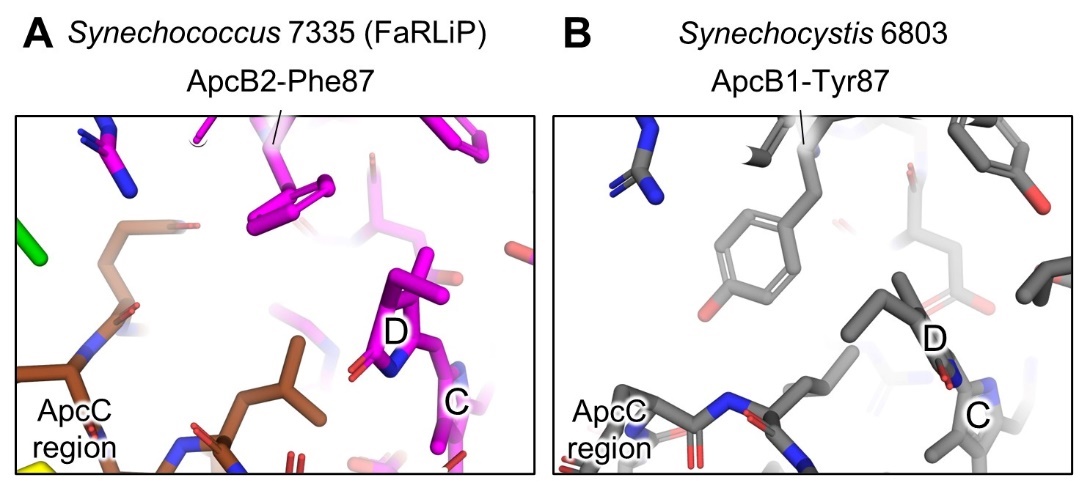


**Figure S21. Protein environment of ApcB2 chain B pyrrole ring D compared to the corresponding environment in ApcB1 pyrrole ring D in the VL-absorbing PBS structure from *Synechocystis* sp. PCC 6803.** *A*, structure of ApcB2 chain B pyrrole ring D environment from the FaRLiP-AP core. *B*, structure of the corresponding ApcB1 pyrrole ring D environment from the VL-absorbing PBS structure (PBD 7SC9).

Supplementary Tables

**Table S1. Selected phycobiliproteins identified by** **chymotryptic peptide fingerprinting by MS/MS spectrometry of the isolated FaRLiP-AP core fraction analyzed by cryo-EM in this study.** Green-shaded entries designate components of the FRL-core complex; the other phycobiliprotein subunits identified are minor contaminating proteins produced in cells grown in VL and are components of VL-PBS. Other minor contaminating proteins included subunits of ribulose-1,5-bisphosphate carboxylase/oxygenase), glutamine synthetase, chaperonin-60, ATP synthase, ferritin, and DNA polymerase.

| **Gene** | **Locus Tag** | **Sequest Score** | **Description** |
| --- | --- | --- | --- |
| *apcE2* | S7335_3294 | 132.36 | Phycobiliprotein core-membrane linker, ApcE2 |
| *apcB2* | S7335_2392 | 98.69 | Allophycocyanin, beta subunit, ApcB2 |
| *apcD5* | S7335_4229 | 75.67 | Allophycocyanin, alpha subunit, ApcD5 |
| *apcD2* | S7335_3274 | 59.67 | Allophycocyanin, alpha subunit, ApcD2 |
| *apcF* | S7335_1487 | 37.38 | Allophycocyanin, beta-18 subunit, ApcF |
| *apcD3* | S7335_4445 | 18.66 | Allophycocyanin, alpha subunit, ApcD3 |
| *apcB1* | S7335_4775 | 13.7 | Allophycocyanin, beta subunit, ApcB1 |
| *cpcA2* | S7335_2244 | 11.6 | Phycocyanin, alpha subunit, CpcA2 |
| *apcA1* | S7335_4074 | 10.49 | Allophycocyanin, alpha subunit, ApcA1 |
| *apcE1* | S7335_1839 | 9.38 | Phycobiliprotein core-membrane linker, ApcE1 |
| *cpcB2* | S7335_4452 | 8.56 | Phycocyanin, beta subunit, CpcB2 |
| *apcD1* | S7335_2013 | 8.55 | Allophycocyanin-B, alpha subunit, ApcD1 |
| *cpcH/I* | S7335_4519 | 8.47 | Peripheral rod linker, CpcH/CpcI |
| *cpcG* | S7335_2387 | 8.21 | Rod-core linker, CpcG |
| *cpeA* | S7335_2962 | 7.91 | Phycoerythrin, alpha subunit, CpeA |
| *cpcH/I* | S7335_4033 | 4.53 | Peripheral rod linker, CpcH/CpcI |
| *cpcL* | S7335_4257 | 2.29 | Rod-core linker, CpcL |
| *apcC* | S7335_3276 | 1.96 | Allophycocyanin core linker, ApcC |

**Table S2.** **Cryo-EM data statistics for the FaRLiP-AP core and Rubisco complexes.**

| **Data collection and processing** | **FaRLiP AP Core** | **Rubisco** |
| --- | --- | --- |
| Magnification | ×105,000 | |
| Voltage (kV) | 300 | |
| Electron exposure (e^-^Å^-2^) | 50.0 | |
| Defocus range (µm) | –0.8 to –2.2 | |
| Pixel size (Å) | 0.4125 | |
| Symmetry imposed | C1 | D4 |
| Initial particle images (no.) | 6,708,708 | 6,708,708 |
| Final particle images (no.) | 215,955 | 57,918 |
| Map resolution (Å) | 2.78 | 2.35 |
| FSC threshold | 0.143 | 0.143 |
| **Refinement** |  |  |
| Initial model used (PDB code) | 8DDY and 7SC7 | 2V63 and 1RSC |
| Model resolution (Å) | 3.08 | 2.45 |
| FSC threshold | 0.5 | 0.5 |
| Map resolution range (Å) | 2.55-3.55 | 1.95-3.15 |
| Map-sharpening *B* factor (Å^2^) | –58.9 | –57.4 |
| Model composition |  |  |
| Non-hydrogen atoms | 27,055 | 37,200 |
| Protein residues | 3,369 | 4,576 |
| Non-water ligands | 19 | 16 |
| Waters | 0 | 904 |
| *B* factors (Å^2^) |  |  |
| Protein | 32 | 8.6 |
| Ligands | 25 | 12.5 |
| R.m.s. deviations |  |  |
| Bond lengths (Å) | 0.010 | 0.007 |
| Bond angles (°) | 1.329 | 1.319 |
| **Validation** |  |  |
| MolProbity | 2.28 | 2.04 |
| Clashscore | 13.19 | 13.72 |
| Rotamer outliers (%) | 4.16 | 1.52 |
| Ramachandran plot |  |  |
| Favored (%) | 96.94 | 96.22 |
| Allowed (%) | 2.85 | 3.78 |
| Disallowed (%) | 0.21 | 0 |

**Table S3.** **Sequence identity comparing selected AP family α-subunits.** Values are reported in percent. This table corresponds to the multiple sequence alignment shown in **Figure S13**.

|  | **ApcA1** | **ApcD1** | **ApcD2** | **ApcD3** | **ApcD4** | **ApcD5** |
| --- | --- | --- | --- | --- | --- | --- |
| **ApcA1** |  | 50.98 | 44.00 | 34.67 | 41.61 | 42.67 |
| **ApcD1** | 50.98 |  | 41.14 | 39.24 | 45.22 | 44.30 |
| **ApcD2** | 44.00 | 41.14 |  | 40.25 | 46.50 | 48.10 |
| **ApcD3** | 34.67 | 39.24 | 40.25 |  | 44.59 | 50.00 |
| **ApcD4** | 41.61 | 45.22 | 46.50 | 44.59 |  | 49.04 |
| **ApcD5** | 42.67 | 44.30 | 48.10 | 50.00 | 49.04 |  |

**Table S4.** **C_α_ superpositions comparing selected AP family α-subunits.** Values are reported in Å.

|  | **ApcA1** | **ApcD1** | **ApcD2** | **ApcD3** | **ApcD4** | **ApcD5** |
| --- | --- | --- | --- | --- | --- | --- |
| **ApcA1** |  | 0.648 | 0.835 | 1.268 | 1.252 | 0.876 |
| **ApcD1** | 0.648 |  | 0.842 | 1.295 | 0.869 | 0.656 |
| **ApcD2** | 0.835 | 0.842 |  | 1.416 | 0.995 | 0.739 |
| **ApcD3** | 1.268 | 1.295 | 1.416 |  | 0.976 | 1.162 |
| **ApcD4** | 1.252 | 0.869 | 0.995 | 0.976 |  | 0.595 |
| **ApcD5** | 0.876 | 0.656 | 0.739 | 1.162 | 0.595 |  |

**Table S5.** **Sequence identity comparing selected AP family β-subunits.** Values are reported in percent. This table corresponds to the multiple sequence alignment shown in **Figure S16**.

|  | **ApcB1** | **ApcB2** | **ApcB3** | **ApcF** |
| --- | --- | --- | --- | --- |
| **ApcB1** |  | 70.81 | 62.11 | 51.55 |
| **ApcB2** | 70.81 |  | 62.73 | 46.58 |
| **ApcB3** | 62.11 | 62.73 |  | 44.72 |
| **ApcF** | 51.55 | 46.58 | 44.72 |  |

**Table S6. C_α_ superpositions comparing selected AP family β-subunits.** Values are reported in Å.

|  | **ApcB1** | **ApcB2** | **ApcB3** | **ApcF** |
| --- | --- | --- | --- | --- |
| **ApcB1** |  | 0.528 | 0.579 | 0.732 |
| **ApcB2** | 0.528 |  | 0.575 | 0.649 |
| **ApcB3** | 0.579 | 0.575 |  | 1.406 |
| **ApcF** | 0.732 | 0.649 | 1.406 |  |

**Table S7. Linker protein interactions with pyrrole ring D of the phycocyanobilins attached to β-subunits.**

| **Chromophore bound to β-subunit** | **Interacts primarily with residues from** |
| --- | --- |
| ApcB2 (chain B) | ApcC, ~residue 23 (chain S) |
| ApcB2 (chain D) | ApcC, ~residue 40 (chain S) |
| ApcB2 (chain F) | ApcE2, ~residue 320 (chain K) |
| ApcB2 (chain H) | ApcE2, ~residue 350 (chain K) |
| ApcF (chain J) | ApcE2, ~residue 420 (chain K) |
| ApcB2 (chain L) | ApcE2, ~residue 432 (chain K) |
| ApcB2 (chain N) | ApcE2, ~residue 468 (chain K) |
| ApcB2 (chain P) | ApcE2, ~residue 601 (chain K) |
| ApcB2 (chain R) | No obvious interactions (probably waters) |
